# Supplementary material for: Adapting the BOADICEA breast and ovarian cancer risk models for the ethnically diverse UK population
Source: Br J Cancer. 2025 Jul 17;133(6):844–55. doi: 10.1038/s41416-025-03117-y (PMC12449465; doi:10.1038/s41416-025-03117-y)
Supplement: Supplementary file 1 — Supplementary Materials [file 41416_2025_3117_MOESM1_ESM.pdf]

SUPPLEMENTARY MATERIALS

Methods

Underlying cancer models

The incidences of breast cancer (BC) and epithelial tubo-ovarian cancer (EOC) for individual i at age t, $\lambda^{(i)}(t)$, depend on the baseline incidence $\lambda_0(t)$ and on their underlying genotype:

$$\lambda^{(i)}(t) = \lambda_0(t)\exp\left(\sum_{\mu=1}^{N_{MG}+1}\left[\left(\beta_\mu(t) + \sum_\rho \boldsymbol{\beta}_{RF\rho\mu}(t)\cdot \boldsymbol{z}_{RF\rho}^{(i)}\right)\prod_{\nu=1}^{\mu-1}\left[\left(1-G_\nu^{(i)}\right)\, G_\mu^{(i)}\right]\right] + \beta_{PG}(t)\, x_P^{(i)}\right)$$

Considering N_{MG} major genes in the model, $G_\mu^{(i)}$ is the indicator variable for the presence of PV in a major gene μ ($\mu = 1,\dots,N_{MG}$), and $\beta_\mu(t)$ represent the age-specific log-relative risks (log-RRs) associated with μ relative to the baseline incidence. $G_{N_{MG}+1}^{(i)} = 1$ for non-carriers of any PV. $x_P^{(i)}$ is the polygenotype for individual i, assumed normally distributed in the general population (mean 0, standard deviation 1); $\beta_{PG}(t)$ is the age-specific log-RR per standard deviation associated with the polygene, relative to the baseline incidence.

Given a questionnaire-based risk factor (QRF) ρ and a major gene μ, $\beta_{RF\rho\mu}(t)$ is the vector of age-specific log-RRs for ρ; $z_{RF\rho}^{(i)}$ is the corresponding vector of indicator variables (0 or 1) that indicate the category of QRF ρ for individual i. The models assume that RRs associated with PVs are log-additive (multiplicative) with the RRs associated with QRFs and the polygenic component.

For a given individual, family history determines their prior probability of carrying PVs in the major genes and the polygenic component. Together with their own phenotype (personal cancer history, QRFs) and gene test results, they are used in calculating the individual's cancer risk and their posterior probability of carrying PVs in the major genes. Family history comprises breast, ovarian, pancreatic and prostate cancers because they are all associated with PVs in some of the major genes considered in the BC and EOC models (1, 2, 3).

Regarding the major genes, the BC model includes the effects of PVs in *BRCA1*, *BRCA2*, *PALB2*, *CHEK2*, *ATM*, *BARD1*, *RAD51C* and *RAD51D* (1), whereas the EOC model includes the effects of PVs in *BRCA1*, *BRCA2*, *RAD51D*, *RAD51C*, *BRIP1* and *PALB2* (3). Both models also consider the associations of PVs in *BRCA1, BRCA2,* and *PALB2* with pancreatic and prostate cancer (2, 3).

Ancestries and ethnicities considered

Genetic ancestry refers to the 'complex inheritance of one's genetic material' (4), 'an individual's biological ancestors'. All individuals have ancestors originating from multiple different locations at different time depth; so, it is more accurate to refer to proportions of genetic ancestries (5) noting that, while groupings allow analytical simplification, genetic ancestry is essentially continuous (5). Statistical methods identify groups of individuals with high (genetic) affinity, even though these

measures are sensitive to the choice and labelling of reference populations (6). This allows measuring the *genetic* similarity between populations and individuals.

Ethnicity refers to a social group identity that is based on shared characteristics, such as cultural traditions, ancestry, language, religion, or social experiences. Ethnicity may vary according to geographical regions and can traverse different social classes, but the shared social experiences of an ethnic group can have health impacts over time (5). Self-identified ethnicity could therefore influence behavioural choices and risk factors.

Datasets used in the modelling process

UK Biobank

The UK Biobank dataset (7, 8) was used to determine ancestry-specific PGS distributions and ethnicity-specific QRFs distributions. A total of 226,340 women and 200,073 men without a cancer diagnosis (self-reported or registry reported-, excluding non-melanoma skin cancers) prior to or within six months of the start of follow-up were included in the analyses. Recruitment of volunteers took place between 2006 and 2010, and the resource includes detailed questionnaire, electronic health record and genetic data (9). Genotyping and sample quality control have been described previously (10). UK Biobank samples were genotyped using Affymetrix UK BiLEVE Axiom array and Affymetrix UK Biobank Axiom array and imputed to the combined 1000 Genomes Project v.3 and UK10K reference panels using SHAPEIT3 and IMPUTE3.18. Samples were included on the basis of female sex (genetic and self-reported). Duplicates, individuals with high degree of relatedness (>10 relatives), and one of each related pair of first-degree relatives were removed. Samples were also excluded using standard quality control criteria.

Genetically inferred ancestry information in UK Biobank was derived using iAdmix (11), which estimates maximum likelihood admixture proportions for an individual's ancestry using population allele frequencies from the 1000 Genomes Project. We estimated the ancestry proportions for four global populations: African, East Asian, European, South Asian. Individuals were classified as 'Mixed' if the proportion of their main ancestry did not reach 80% (70% for South Asian).

KARMA

The KARolinska Mammography Project for Risk Prediction of Breast Cancer (KARMA) cohort is a large prospective population-based cohort (12) of Swedish women (N=66,415), age range 40-74. For each participant, it contains data first-degree cancer family history (FH) and QRFs (self-reported), mammographic density (MD) as cBIRADS (13) and cancer diagnoses/deaths (from linkages to healthcare registers). KARMA was previously used for validating BOADICEA (14); here, we used it to assess the impact of using the UK Biobank QRFs distributions on the predicted risks.

MyBrCa and SGBCC

The proportions of the overall polygenic component explained by the PGS ('statistical methods' section) in women of East and South Asian ancestry were derived using data (15, 16) from two studies, from Malaysia (MyBrCa (17)) and Singapore (SGBCC (18)), participating in the Breast Cancer Association Consortium (BCAC).

For MyBrCa, cases were a mixture of prevalent and incident BCs, identified at the Breast Cancer Clinic in University Malaya Medical Centre (from January 2003 to July 2014) and Subang Jaya Medical Centre (from September 2012 to September 2014). Controls were cancer-free individuals (37-74 years) selected from women attending mammographic screening at the same hospitals. For SGBCC, cases were a mixture of prevalent and incident BCs (in situ or invasive), diagnosed at National University Hospital between 2006 and 2013. Controls were Singaporeans or Singaporean Permanent Residents, 21 years and older; they were recruited between 2006 and 2010 through word-of-mouth, personal recommendations, 'cold-calling' and door-to-door invitations.

All samples were genotyped on the OncoArray as described previously (19). Analyses were restricted to women with age of diagnosis or last observation less than 80 years and classified into either 'East Asian' or 'South Asian' according to genetic data. Women were from three main self-reported ethnic groups: Malay, Chinese and Indian. For analyses of East Asian women, samples from Chinese and Malay women were combined, and only women classified as 'East Asian' according to genetic data were included in the analyses. The South Asian group comprised women self-reporting as Indian that were also classified as 'South Asian' according to genetic data. **Table S15** reports studies, number of participants by ethnicity, and mean/SD age at diagnosis or interview (for cases and controls, respectively).

Breast Cancer Surveillance Consortium

To estimate the distributions of breast density (using BIRADS categories) for each ethnicity, and to calculate the corresponding odds ratios (ORs) for the association with BC risk, we used the public version of the risk estimation dataset from the Breast Cancer Surveillance Consortium (BCSC dataset, 2012 version) (20). It contains information on 1,007,657 unaffected women that had mammograms between 1996 and 2002. Each entry contains data on MD (as BIRADS category), age at screening (as 5-year aggregate), 'ethnicity' (Hispanic or not), 'race' (White, Asian, Black, Native American, Other/Mixed), number of first-degree relative affected with BC, and some QRFs (BMI, age at first birth, hormone replacement therapy (HRT) use). BCSC also has data on previous surgical procedures on breast or surgical menopause. For each woman, it is recorded whether she developed invasive BC within 1 year after the mammogram; due to data aggregation and anonymisation, the exact timing of the cancer diagnosis is unknown.

Cancer incidences and tumour subtype distributions

Ethnicity-specific population cancer incidences

For each cancer type (breast, ovarian, pancreatic, prostate), we considered that

$$I_{pop} = I_{asian} * P_{asian} + I_{black} * P_{black} + I_{mixed} * P_{mixed} + I_{white} * P_{white} + I_{other} * P_{other}$$

$$= I_{white} * (R_{asian} * P_{asian} + R_{black} * P_{black} + R_{mixed} * P_{mixed} + 1 * P_{white}) + I_{pop} * P_{other}$$

where I_{pop} are the BOADICEA sex and cohort-specific population incidences and $I_{ethnicity}$ are the sex, cohort and ethnicity-specific incidences. $R_{ethnicity}$ are the published incidence rate ratios (21) ($R_{white} = 1$). Published incidence rate ratios were available for the <65 and the ≥65 years age groups. However, there was very little difference in the ratios between the two age groups for the different ethnicities, or there were very few cases in one of the two groups. Therefore, for simplicity, we assumed that the proportions were constant with age. $P_{ethnicity}$ are the ethnicity proportions obtained

from the ONS (22). We used the ethnicity proportions from the 2016 census, matching the timespan (2013-2017) covered in the paper from Delon *et al.*, 2022 (21). $R_{ethnicity}$ and $P_{ethnicity}$ can be found in **Table S1**.

We defined

$$\theta = \frac{(1 - P_{other})}{(R_{asian} * P_{asian} + R_{black} * P_{black} + R_{mixed} * P_{mixed} + 1 * P_{white})}$$

thus obtaining the cancer incidences for each sex, cohort and ethnic group:

$$I_{ethnicity} = I_{pop} * R_{ethnicity} * \theta$$

The published incidence rate ratios (21) grouped East and South Asians together due to sample size considerations, and no data were available for these groups separately in England. Consequently, they have been considered as a single category in this analysis, yielding cancer incidences for 'Asians' as a whole.

Gene- and ethnicity- specific tumour subtype distributions

The age-specific subtype proportions for each ethnicity group were based on published estimates from NCRAS (National Cancer Registration and Analysis Service) data (23, 24). Those analyses involved fitting logistic regression models to predict the probability of the breast cancer (BC) subtype, after imputing missing data. Age at diagnosis was modelled using quadratic splines with knots placed at 35, 45 and 55 for the ER-positive subtype and 35 and 45 for the TN and ER-negative non-TN subtypes. Additionally, an interaction term between the age at BC diagnosis and ethnicity was included in each model (23, 24).

Genetic components

Polygenic Scores

PGS parameters estimation for breast cancer

The retrospective likelihood approach requires overall population age-specific incidence rates to be specified. In these analyses, population age-specific incidence rates by country (Singapore or Malaysia) and ethnicity (Malay, Chinese or Indian) were derived from the Singapore Cancer Registry (2013-2017) and the Penang Cancer Registry (2012-2016). Covariates included in these analyses were the country of origin of the study and 10 PCs (25). While the α_{RL} approach explicitly models the unmeasured polygenic component, another approach to estimating α makes the simplifying assumption that the polygenic standard deviation (SD) of the known polygenic component in BOADICEA can be approximated by the marginal age-specific log-odds ratio (log-OR) per unit SD of the PGS. The α parameter obtained with this method is denoted α_{GLM}.

The mean and SD, the log-OR per unit SD of PGSs, and the parameters α_{GLM} and α_{RL} were estimated separately for East Asian and South Asian women.

For African women, there were insufficient data to estimate α directly. Therefore, α was estimated from published estimates of the OR per 1 SD, assuming that α is proportional to the OR. The ORs estimates from the three studies (26-28) were similar, with a combined estimate of 1.27 (95% CI:

1.23-1.31), based on a fixed-effect meta-analysis. Thus, using East Asians for comparisons, we calculated:

$$\alpha_{African} = \ln(1.27) * \alpha_{EastAsian} / \ln(OR)_{EastAsian}$$

East Asians were used as the reference because (a) the population is arguably more comparable to Africans than Europeans (lower risk, higher proportion of ER-negative etc.) and (b) this is a more conservative assumption. The populations differ with reference to allele frequencies and linkage disequilibrium; however, these affect the OR per 1 SD too and, in the absence of other evidence, the proportionality assumption seems reasonable.

PGS parameters estimation for ovarian cancer

For each SNP i, the ancestry-specific allele frequency f_i was estimated using Plink2 from genotyped and imputed data in UK Biobank. Assuming the log-odds ratio β_i (from OCAC estimates) associated with each SNP remained the same across ancestries, the variance explained by the PGS was calculated using the formula (3):

$$\sigma_{PGS}^2 = \sum_{i=1}^{36} \ln\left(\frac{(1-f_i)^2 + 2(1-f_i)f_i e^{2\beta_i} + f_i^2 e^{4\beta_i}}{\left((1-f_i)^2 + 2(1-f_i)f_i e^{\beta_i} + f_i^2 e^{2\beta_i}\right)^2}\right)$$

The square root of the proportion of the overall polygenic variance explained by the PGS, namely α_i for each ancestry, was calculated as:

$$\alpha = \frac{\sigma_{PGS}}{1.4156}$$

where 1.41562 is the EOC polygenic SD assumed in the model (3). Note that those σ_{PGS} differ slightly from the SDs given in **Table S13**, which are based on the distribution of the PGS in individuals without cancer in UK Biobank.

PGS for individuals of mixed genetic ancestry

Normalised polygenic scores are employed in BOADICEA; therefore, they depend on ancestry even though the raw PGS is the same. For ancestry i, we have:

$$R_{pgs} = \sum_{k=1}^{K} \beta_k g_k, \qquad Z_i = \frac{(R_{prs} - \mu_i)}{\sigma_i}$$

where

$$\mu_i = E(R_{prs}|pop\ i) = 2\sum_{k=1}^{K} w_k \rho_{ik}, \qquad \sigma_i^2 = var(pop\ i) = 2\sum_{k=1}^{K} \rho_{ik}(1-\rho_{ik}) w_k^2$$

ρ_{ik} being the ancestry-specific frequency of locus k and w_k being the weight of locus k, i.e. (usually and in this work) the fixed per-allele log-odds ratio β_k.

For 'Mixed' individuals, PGS must be normalised taking into account the proportion of each ancestry; the overall *alpha* (α) must be similarly transformed. Given P_i proportion, γ_i effect size and Z_i normalised PGS for ancestry i, we can define

$$S = \sum_{i=1}^{M} P_i \gamma_i Z_i$$

so that

$$E(S) = 0, \qquad var(S) = \left(\sum_{i=1}^{M} \frac{P_i \gamma_i}{\sigma_i}\right)^2 var(R_{prs}) \equiv \left(\sum_{i=1}^{M} \frac{P_i \gamma_i}{\sigma_i}\right)^2 \sigma^2$$

and therefore

$$Z = \frac{S - E(S)}{sd(S)} = \frac{\sum_{i=1}^{M} P_i \gamma_i Z_i}{\sigma \sum_{i=1}^{M} \frac{P_i \gamma_i}{\sigma_i}}, \qquad \alpha = \frac{\sum_{i=1}^{M} P_i \alpha_i \sigma_i}{\sigma}$$

Clearly, σ depends on the proportion of each ancestry, but it also considers that different loci could belong to different ancestries. Indeed we have

$$\sigma^2 = var(x_{prs}) = E(var(pop\ i)) + var(E(pop\ i)) = E(\sigma_i^2) + var(\mu_i)$$
$$= \sum_{i=1}^{M} P_i \sigma_i^2 + 4 \sum_{k=1}^{K} w_k^2 \left(\sum_{i=1}^{M} P_i {\rho_{ik}}^2 - \left(\sum_{i=1}^{M} P_i \rho_{ik} \right)^2 \right)$$

The full formula derivation can be found at the end of these Supplementary Materials.

Rare genetic variants

Literature search on risks associated with rare genetic variants

For the non-European populations, using the searching keywords ‘breast cancer’, ‘germline pathogenic variants’ and ‘Asian’ returned 75 articles. Using the searching keywords ‘breast cancer’, ‘germline pathogenic variants’ and ‘African’ returned 35 articles. Using the searching keywords ‘ovarian cancer’, ‘germline pathogenic variants’ and ‘Asian’ returned 30 articles. Using the search keywords ‘ovarian cancer’, ‘germline pathogenic variants’ and ‘African’ returned 9 articles.

Mammographic Density

In this analysis, we employed the public version of the BCSC dataset (2012 version), discarding data on women that had previous surgical procedures, obtaining a dataset of 718,889 entries. As ethnicities, we considered all combinations of the ‘race’ and ‘ethnicity’ categories in the dataset; however, we merged the ‘Other’ and ‘Native American’ categories, and we did not consider Hispanic women in the downstream analysis. **Table S2** shows the number of unaffected and affected women by ethnicity.

We employed Multivariate Imputation by Chained Equations (MICE, (29)) to impute missing values in the following categories: ‘ethnicity’ (Hispanic or not), body mass index (BMI), number of affected (1st degree) relatives, age at first live birth (for parous women), age at menopause and use of hormone replacement therapy (HRT) if applicable. Ten imputed datasets were generated through 20 iterations. **Table S3** shows the (average) number of unaffected and affected women by ethnicity after imputation.

We calculated ORs using logistic regression on the imputed datasets (*survival* R package, (30)), adjusting for all BOADICEA QRFs contained in the dataset and stratifying by age group (before age 50y, after age 50y). Two different methods were used for calculating ethnicity-specific ORs: analysing the entire dataset and fitting an interaction term between MD and ethnicity; and analysing each ethnic group separately. The ORs calculated from the imputed datasets were combined using the Rubin's rule; D_2 pooling method was applied to pool the statistics from likelihood-ratio tests.

In this analysis, Hispanics were considered as separate ethnic groups (White Hispanics, Black Hispanics etc.). However, since this ethnic group not considered in this work, their respective ORs were not reported in this manuscript, and the distribution of BIRADS categories was calculated for non-Hispanic women only.

Assessing risk stratification

We considered all possible combinations of risk factors in the model. For each combination, we calculated its probability as a function of the joint risk factor distributions used in the model. These probabilities can be considered as proportions of risk factors combinations in a hypothetical UK population. In formulas:

- Each model considers *N* risk factors ($R_1 \ldots R_N$), each one having a fixed number of categories considered ($\#_1 \ldots \#_N$). Continuous risk factors (e.g. height, PGS) can be discretized using a suitable number of categories (2). Therefore, *M* possible combinations exist, where

$$M = \prod_{k=1}^{N} \#_k$$

- Each combination can be written as $C = \{r_1 \ldots r_N\}, r_k \in R_k$, where r_k is the selected category for the predictor R_k. The proportion of each $r_k \in R_k$ is ethnicity- or ancestry-specific, as described in previous sections.
- The likelihood of each combination C is calculated as the product of the probabilities the categories (from each risk predictor) composing that combination, i.e.

$$P(C) = \prod_{k=1}^{N} P(r_k)$$

- The models consider risk predictors to be independent from each other, thus justifying the use of this formula for defining the reference population of each model.

Results

Cancer incidences and tumour subtype distributions

Gene- and ethnicity- specific tumour subtype distributions

In White women, the proportion of ER-positive tumours increases with age and then plateaus at approximately age 50 years. The proportion of ER-positive tumours in East- and South-Asian women also increase with age but are lower than for Whites. In Black women, the proportions of ER-positive disease are lower and varies little with age. Conversely, Black women are more likely to be diagnosed with triple negative (TN) disease, with the proportion varying little with age. In White and Asian women, the proportion of TN cases decreases with age, but is higher in Asian women than in White women.

Age-, gene- and subtype specific proportions were calculated for women of White, Black, East Asian, South Asian, and Mixed ethnicity. Proportions in the 'Population' were based on 1) non-carrier proportions published in JAMA Oncology (31) and 2) general population published estimates from NCRAS data (23, 24). Estimates for White non-carrier women in BCAC dataset (from BRIDGES) (31) are also shown for comparison with NCRAS data (smoothed using *lowess* and quadratic splines, respectively).

Genetic components

Rare genetic variants

Five large studies have examined the associations between rare PVs and BC risk in individuals from different ethnicities/ancestries. The BRIDGES study (Breast Cancer Risk after Diagnostic Gene Sequencing) analysed a 34-gene panel using data from the Breast Cancer Association Consortium (BCAC).The study included 42,060 breast cancer cases of European ancestry and 6,279 cases of Asian ancestry, along with 44,035 cancer-free controls of European ancestry and 6,047 cancer-free controls of Asian ancestry (32). The CARRIERS sequenced the same gene panel in 32,247 BC cases and 32,544 controls. The study population included 1,282 BC cases and 1,269 controls of Asian ancestry, 3,946 cases and 4,954 controls of African American ancestry, and 25,287 cases and 24,770 controls of non-Hispanic White ancestry (33). The Ghana Breast Health Study (GBHS) sequenced the same gene panel in 871 cases and 1563 controls of African ancestry (34). The African American study sequenced 37-gene panel in 5054 cases and 4993 controls of African American ancestry (35). The Japanese study sequenced 11 BC genes in 7051 BC cases and 11241 controls of Japanese ancestry (36). An additional study estimated BC and EOC relative risks associated with *BRCA1* and *BRCA2* in 572 families from Malaysia and Singapore (37).

To estimate the overall *CHEK2* PV frequency in non-Europeans, we used the frequency of other (non c.1100del) PVs in controls in the BRIDGES dataset, combined with the c.1100delC frequency in East Asians. We adjusted for an assumed sensitivity of 0.957 and a proportion (0.16) of large rearrangements that would have been being undetectable (1). We assumed this frequency applied also to the other non-European ancestries.

Mammographic Density

The previous implementation of BOADICEA (BC model v6) assumed that QRFs distributions do not vary with age; for density it uses distributions for White women younger than 50 years, published in Tice *et al.*, 2008 (38). For consistency, here we implemented the distributions from the <50 years age group for all ethnicities. This coarse simplification does not consider the relevant change in MD with age; a concurrent model extension, based on continuous MD measures, incorporates age-specific MD density distributions into BOADICEA (39) for all ethnicities.

Multifactorial models

Table S22 summarises the ethnicity and ancestry-specific data used for each model component. If known, genetic ancestry is employed for specifying the PGS model; *CHEK2 PV* frequency is always assumed from the self-reported ethnicity to allow for the fact that in practice not everyone with gene-panel sequencing will have genetic ancestry determined. When ethnicity is unknown, the default, population-wide parameters are employed. When the self-reported ethnicity is 'Other', the model assumes the default parameters apart from the *CHEK2* PV frequency, where the frequency for non-Europeans is used.

References

1. Antoniou AC, Cunningham, AP, Peto J, Evans DG, Lalloo F, Narod SA, et al. The BOADICEA model of genetic susceptibility to breast and ovarian cancers: updates and extensions. Br J Cancer. 2008;98(8):1457-66.
2. Lee A, Mavaddat N, Cunningham A, Carver T, Ficorella L, Archer S, et al. Enhancing the BOADICEA cancer risk prediction model to incorporate new data on RAD51C, RAD51D, BARD1 updates to tumour pathology and cancer incidence. J Med Genet. 2022;59(12):1206-18.
3. Lee A, Yang X, Tyrer J, Gentry-Maharaj A, Ryan A, Mavaddat N, et al. Comprehensive epithelial tubo-ovarian cancer risk prediction model incorporating genetic and epidemiological risk factors. J Med Genet. 2022;59(7):632-43.
4. Constantinescu AE, Mitchell RE, Zheng J, Bull CJ, Timpson NJ, Amulic B, et al. A framework for research into continental ancestry groups of the UK Biobank. Hum Genomics. 2022;16(1):3.
5. Caliebe A, Tekola-Ayele F, Darst BF, Wang X, Song YE, Gui J, et al. Including diverse and admixed populations in genetic epidemiology research. Genet Epidemiol. 2022;46(7):347-71.
6. Mathieson I, Scally A. What is ancestry? PLoS Genet. 2020;16(3):e1008624.
7. Sudlow C, Gallacher J, Allen N, Beral V, Burton P, Danesh J, et al. UK biobank: an open access resource for identifying the causes of a wide range of complex diseases of middle and old age. PLoS Med. 2015;12(3):e1001779.
8. UK Biobank. [03/02/2025]. Available from: https://www.ukbiobank.ac.uk/.
9. Bycroft C, Freeman C, Petkova D, Band G, Elliott LT, Sharp K, et al. The UK Biobank resource with deep phenotyping and genomic data. Nature. 2018;562(7726):203-9.
10. Mavaddat N, Michailidou K, Dennis J, Lush M, Fachal L, Lee A, et al. Polygenic Risk Scores for Prediction of Breast Cancer and Breast Cancer Subtypes. Am J Hum Genet. 2019;104(1):21-34.
11. Bansal V, Libiger O. Fast individual ancestry inference from DNA sequence data leveraging allele frequencies for multiple populations. BMC Bioinformatics. 2015;16:4.
12. Gabrielson M, Eriksson M, Hammarstrom M, Borgquist S, Leifland K, Czene K, et al. Cohort Profile: The Karolinska Mammography Project for Risk Prediction of Breast Cancer (KARMA). Int J Epidemiol. 2017;46(6):1740-1g.
13. Eriksson M, Czene K, Pawitan Y, Leifland K, Darabi H, Hall P. A clinical model for identifying the short-term risk of breast cancer. Breast Cancer Res. 2017;19(1):29.
14. Yang X, Eriksson M, Czene K, Lee A, Leslie G, Lush M, et al. Prospective validation of the BOADICEA multifactorial breast cancer risk prediction model in a large prospective cohort study. J Med Genet. 2022;59(12):1196-205.
15. Ho WK, Tai MC, Dennis J, Shu X, Li J, Ho PJ, et al. Polygenic risk scores for prediction of breast cancer risk in Asian populations. Genet Med. 2022;24(3):586-600.
16. Ho WK, Tan MM, Mavaddat N, Tai MC, Mariapun S, Li J, et al. European polygenic risk score for prediction of breast cancer shows similar performance in Asian women. Nat Commun. 2020;11(1):3833.
17. Tan MM, Ho WK, Yoon SY, Mariapun S, Hasan SN, Lee DS, et al. A case-control study of breast cancer risk factors in 7,663 women in Malaysia. PLoS One. 2018;13(9):e0203469.
18. Ho PJ, Yeoh YS, Miao H, Lim SH, Tan EY, Tan BKT, et al. Cohort profile: The Singapore Breast Cancer Cohort (SGBCC), a multi-center breast cancer cohort for evaluation of phenotypic risk factors and genetic markers. PLoS One. 2021;16(4):e0250102.
19. Michailidou K, Lindstrom S, Dennis J, Beesley J, Hui S, Kar S, et al. Association analysis identifies 65 new breast cancer risk loci. Nature. 2017;551(7678):92-4.
20. Barlow WE, White E, Ballard-Barbash R, Vacek PM, Titus-Ernstoff L, Carney PA, et al. Prospective breast cancer risk prediction model for women undergoing screening mammography. J Natl Cancer Inst. 2006;98(17):1204-14.

21. Delon C, Brown KF, Payne NWS, Kotrotsios Y, Vernon S, Shelton J. Differences in cancer incidence by broad ethnic group in England, 2013-2017. Br J Cancer. 2022;126(12):1765-73.
22. Office for National Statistics. Population estimates by ethnic group and religion, England and Wales: 2019. 2021 [26/06/2023]. Available from: https://www.ons.gov.uk/peoplepopulationandcommunity/populationandmigration/populationestimates/articles/populationestimatesbyethnicgroupandreligionenglandandwales/2019.
23. Hassan H, Allen I, Rahman T, Bacon A, Knott C, Huntley C, et al. Long-term outcomes of BSO in women with personal history of breast cancer. BMJ Oncol. 2025; 4(1):e000574.
24. Hassan H. Long-term outcomes of bilateral salpingo-oophorectomy in the general population and in women with pathogenic variants in BRCA1 and BRCA2 [Unpublished PhD thesis]: University of Cambridge; 2025.
25. Mavaddat N, Ficorella L, Carver T, Lee A, Cunningham AP, Lush M, et al. Incorporating Alternative Polygenic Risk Scores into the BOADICEA Breast Cancer Risk Prediction Model. Cancer Epidemiol Biomarkers Prev. 2023;32(3):422-7.
26. Du Z, Gao G, Adedokun B, Ahearn T, Lunetta KL, Zirpoli G, et al. Evaluating Polygenic Risk Scores for Breast Cancer in Women of African Ancestry. J Natl Cancer Inst. 2021;113(9):1168-76.
27. Liu C, Zeinomar N, Chung WK, Kiryluk K, Gharavi AG, Hripcsak G, et al. Generalizability of Polygenic Risk Scores for Breast Cancer Among Women With European, African, and Latinx Ancestry. JAMA Netw Open. 2021;4(8):e2119084.
28. Shang H, Ding Y, Venkateswaran V, Boulier K, Kathuria-Prakash N, Malidarreh PB, et al. Generalizability of PGS(313) for breast cancer risk in a Los Angeles biobank. HGG Adv. 2024;5(3):100302.
29. van Buuren S, Groothuis-Oudshoorn K. mice: Multivariate Imputation by Chained Equations in R. J Stat Softw. 2011;45(3):1-67.
30. Therneau TM. A package for survival analysis in R. 3.5-7 ed2023.
31. Breast Cancer Association Consortium, Mavaddat N, Dorling L, Carvalho S, Allen J, Gonzalez-Neira A, et al. Pathology of Tumors Associated With Pathogenic Germline Variants in 9 Breast Cancer Susceptibility Genes. JAMA Oncol. 2022;8(3):e216744.
32. Dorling L, Carvalho S, Allen J, González-Neira A, Luccarini C, Wahlström C, et al. Breast Cancer Risk Genes — Association Analysis in More than 113,000 Women. New England Journal of Medicine. 2021;384(5):428-39.
33. Hu C, Hart SN, Gnanaolivu R, Huang H, Lee KY, Na J, et al. A Population-Based Study of Genes Previously Implicated in Breast Cancer. N Engl J Med. 2021;384(5):440-51.
34. Ahearn TU, Choudhury PP, Derkach A, Wiafe-Addai B, Awuah B, Yarney J, et al. Breast Cancer Risk in Women from Ghana Carrying Rare Germline Pathogenic Mutations. Cancer Epidemiol Biomarkers Prev. 2022;31(8):1593-601.
35. Palmer JR, Polley EC, Hu C, John EM, Haiman C, Hart SN, et al. Contribution of Germline Predisposition Gene Mutations to Breast Cancer Risk in African American Women. J Natl Cancer Inst. 2020;112(12):1213-21.
36. Momozawa Y, Iwasaki Y, Parsons MT, Kamatani Y, Takahashi A, Tamura C, et al. Germline pathogenic variants of 11 breast cancer genes in 7,051 Japanese patients and 11,241 controls. Nat Commun. 2018;9(1):4083.
37. Ho WK, Hassan NT, Yoon SY, Yang X, Lim JMC, Binte Ishak ND, et al. Age-specific breast and ovarian cancer risks associated with germline BRCA1 or BRCA2 pathogenic variants - an Asian study of 572 families. Lancet Reg Health West Pac. 2024;44:101017.
38. Tice JA, Cummings SR, Smith-Bindman R, Ichikawa L, Barlow WE, Kerlikowske K. Using clinical factors and mammographic breast density to estimate breast cancer risk: development and validation of a new predictive model. Ann Intern Med. 2008;148(5):337-47.
39. Ficorella L, Eriksson M, Czene K, Leslie G, Yang X, Carver T, et al. Incorporating continuous mammographic density into the BOADICEA breast cancer risk prediction model. medRxiv. 2025;2025.02.14.25322305.

Figure and table legends

Figure S1. Ethnicity-specific cancer incidences per 100,000 people in the UK, employed in the BC and EOC models (1980-1989 birth cohort). Panel A, breast cancer (BC); panel B, ovarian cancer (OC); panel C, pancreatic cancer (PanC); panel D, prostate cancer (ProC).

Figure S2. *[in separate file]* ER-positive predicted proportions by age at cancer diagnosis and ethnicity, based on published estimates from NCRAS data. Age at diagnosis was modelled using quadratic splines with knots placed at 35, 45 and 55 years.

Figure S3. *[in separate file]* ER-negative non-TN predicted proportions by age at cancer diagnosis and ethnicity, based on published estimates from NCRAS data. Age at diagnosis was modelled using quadratic splines with knots placed at 35 and 45 years.

Figure S4. *[in separate file]* TN predicted proportions by age at cancer diagnosis and ethnicity, based on published estimates from NCRAS data. Age at diagnosis was modelled using quadratic splines with knots placed at 35 and 45 years.

Figure S5. *[in separate file]* ER-positive predicted proportions by age at cancer diagnosis and ethnicity, in PV carriers and in the general population.

Figure S6. *[in separate file]* ER-negative non-TN predicted proportions by age at cancer diagnosis and ethnicity, in PV carriers and in the general population.

Figure S7. *[in separate file]* TN predicted proportions by age at cancer diagnosis and ethnicity, in PV carriers and in the general population.

Figure S8. Standard deviations (SDs) for multiple (random) combinations of ancestry proportions, calculated using the full formula detailed in Methods and by weighted averaging the ancestry-specific SDs. Each random combination is created by successive extraction of ancestry proportions from continuous uniform distributions: the first proportion (P_1) is extracted from $U(0,1)$; the second one (P_2) from $U(0,1-P_1)$; the third one (P_3) from $U(0,1-P_1-P_2)$; the fourth one is set to $P_4 = 1 - P_1 - P_2 - P_3$.

Figure S9. Predicted breast cancer risks by age for a female born in 2000 (Mixed ethnicity, unknown family history and QRFs), based on ancestry and PGS. Ancestry-specific standardised PGS were calculated from the same raw PGS values, corresponding to 10th, 25th, 45th, 55th, 75th and 90th percentile of the standardised PGS distribution in Europeans. 'Mixed (simple)' was obtained with a simplified formula for calculating σ ($\boldsymbol{\sigma^2 = \sum_{i=1}^{M} P_i \sigma_i^2}$); 'Mixed (full)' was obtained with the complete formula for σ.

Figure S10. Comparison of 10-year breast cancer (BC) risk predictions in the KARMA cohort. X-axis shows the BC risks predicted by using the default QRFs distributions in BOADICEA whereas y-axis shows the BC risks predicted by switching to use QRFs distributions estimated in the UK Biobank White populations. The dashed line is the diagonal line with slope equal to 1 (corresponding to no difference in the risk predictions).

Figure S11. Predicted epithelial ovarian cancer risks by age for a female born in 1985 (unknown family history and QRFs) based on ethnicity and pathogenic variant carrier status (for the major genes considered in the EOC model).

Figure S12. Predicted breast cancer risks by age for a female born in 1985 (unknown family history and QRFs) based on ethnicity and pathogenic variant carrier status (for the major genes considered in the BC model).

Figure S13. Predicted lifetime (age 20-80 years) BC risks for women of different ethnicities, untested for PVs and unknown family history. Risks are based on different combinations of risk predictors, each drawn in a separate panel: (A,B) PGS only; (C,D) QRFs only; (E,F) PGS and QRFs; (G,H) PGS and QRFs and MD. (A,C,E,G) Probability density function against absolute risk; (B,D,F,H) absolute risk against cumulative distribution. BC, breast cancer; PGS, polygenic score; QRFs, questionnaire-based risk factors; MD, mammographic density. The backgrounds of the graphs are shaded to indicate three lifetime BC risk categories: less than 17% (light yellow); between 17% and 30% (yellow); above 30% (light blue).

Figure S14. Predicted 10-year (to age 50 years) BC risks for women of different ethnicities, untested for PVs and unknown family history. Risks are based on different combinations of risk predictors, each drawn in a separate panel: (A,B) PGS only; (C,D) QRFs only; (E,F) PGS and QRFs; (G,H) PGS and QRFs and MD. (A,C,E,G) Probability density function against absolute risk; (B,D,F,H) absolute risk against cumulative distribution. BC, breast cancer; PGS, polygenic score; QRFs, questionnaire-based risk factors; MD, mammographic density. The backgrounds of the graphs are shaded to indicate three 10-year BC risk categories: less than 3% (light yellow); between 3% and 8% (yellow); above 8% (light blue).

Figure S15. Predicted lifetime (age 20-80 years) EOC risks for women of different ethnicities, untested for PVs and unknown family history. Risks are based on different combinations of risk predictors, each drawn in a separate panel: (A,B) PGS only; (C,D) QRFs only; (E,F) PGS and QRFs. (A,C,E) Probability density function against absolute risk; (B,D,F) absolute risk against cumulative distribution. EOC, epithelial ovarian cancer; PGS, polygenic score; QRFs, questionnaire-based risk factors. The backgrounds of the graphs are shaded to indicate three lifetime EOC risk categories: less than 3.5% (light yellow); between 3.5% and 5% (yellow); above 5% (light blue).

Figure S16. Predicted 10-year (to age 50 years) EOC risks for women of different ethnicities, untested for PVs and unknown family history. Risks are based on different combinations of risk predictors, each drawn in a separate panel: (A,B) PGS only; (C,D) QRFs only; (E,F) PGS and QRFs. (A,C,E) Probability density function against absolute risk; (B,D,F) absolute risk against cumulative distribution. EOC, epithelial ovarian cancer; PGS, polygenic score; QRFs, questionnaire-based risk factors.

Figure S17. Predicted lifetime (age 20-80 years) BC risks for women of different ethnicities, untested for PVs and unknown family history, based on different combinations of risk predictors. Distributions for each ethnicity are drawn in separate panels: (A,B) Population/Unspecified; (C,D) White; (E,F) Black; (G,H) East Asian; (I,J) South Asian; (K,L) Mixed. The straight lines (labelled 'ethnicity only') are equivalent to the "population" risk of BC for the specific ethnicity plotted. (A,C,E,G,I,K) Probability density function against absolute risk; (B,D,F,H,J,L) absolute risk against cumulative distribution. BC, breast cancer; PGS, Polygenic Score; QRFs, questionnaire-based risk factors; MD, mammographic density. The backgrounds of the graphs are shaded to indicate three lifetime BC risk categories: less than 17% (light yellow); between 17% and 30% (yellow); above 30% (light blue).

Figure S18. Predicted 10-year (to age 50 years) BC risks for women of different ethnicities, untested for PVs and unknown family history, based on different combinations of risk predictors. Distributions for each ethnicity are drawn in separate panels: (A,B) Population/Unspecified; (C,D) White; (E,F) Black; (G,H) East Asian; (I,J) South Asian; (K,L) Mixed. The straight lines (labelled 'ethnicity only') are equivalent to the "population" risk of BC for the specific plotted. (A,C,E,G,I,K) Probability density function against absolute risk; (B,D,F,H,J,L) absolute risk against cumulative distribution. BC, breast cancer; PGS, polygenic score; QRFs, questionnaire-based risk factors; MD, mammographic density. The backgrounds of the graphs are shaded to indicate three 10-year BC risk categories: less than 3% (light yellow); between 3% and 8% (yellow); above 8% (light blue).

Figure S19. Predicted lifetime (age 20-80 years) EOC risks for women of different ethnicities, untested for PVs and unknown family history, based on different combinations of risk predictors. Distributions for each ethnicity are drawn in separate panels: (A,B) Population/Unspecified; (C,D) White; (E,F) Black; (G,H) East Asian; (I,J) South Asian; (K,L) Mixed. The straight lines (labelled 'ethnicity only') are equivalent to the "population" risk of EOC for the specific ethnicity plotted. (A,C,E,G,I,K) Probability density function against absolute risk; (B,D,F,H,J,L) absolute risk against cumulative distribution. EOC, epithelial ovarian cancer; PGS, polygenic score; QRFs, questionnaire-based risk factors. The backgrounds of the graphs are shaded to indicate three lifetime EOC risk categories: less than 3.5% (light yellow); between 3.5% and 5% (yellow); above 5% (light blue).

Figure S20. Predicted 10-year (to age 50 years) EOC risks for women of different ethnicities, untested for PVs and unknown family history, based on different combinations of risk predictors. Distributions for each ethnicity are drawn in separate panels: (A,B) Population/Unspecified; (C,D) White; (E,F) Black; (G,H) East Asian; (I,J) South Asian; (K,L) Mixed. The straight lines (labelled 'ethnicity only') are equivalent to the "population" risk of EOC for the specific ethnicity plotted. (A,C,E,G,I,K) Probability density function against absolute risk; (B,D,F,H,J,L) absolute risk against cumulative distribution. EOC, epithelial ovarian cancer; PGS, polygenic score; QRFs, questionnaire-based risk factors.

Figure S21. Predicted BC and EOC lifetime (age 20-80 years) risks for women of different ethnicities, BRCA2 PV carrier and unknown family history; risks calculated using all risk predictors: PGS, QRFs, and MD. (A,B) BC risk; backgrounds are shaded to indicate three risk categories: $<17\%$ (light yellow); 17% - 30% (yellow); $\geq 30\%$ (light blue). (C,D) EOC risk; backgrounds are shaded to indicate three risk categories: $< 3.5\%$ (light yellow); 3.5% - 5% (yellow); $\geq 5\%$ (light blue). (A,C) probability density function against absolute risk; (B,D) absolute risk against cumulative distribution. BC, breast cancer; EOC, epithelial ovarian cancer; PGS, polygenic score; QRFs, questionnaire-based risk factors; MD, mammographic density.

Table S1. Ethnicity proportions ($P_{ethnicity}$) obtained from the ONS (22) and incidence rate ratios by ethnic group and cancer type ($R_{ethnicity}$), relative to White (21). East and South Asian ethnicities were grouped in a single group due to small numbers.

Table S2. Summary of the BCSC dataset used: distribution of women across all 'races' and 'ethnicities', by age group (split age: 50 years) and by diagnosis within 1 year from the mammogram screening.

Table S3. Summary of the BCSC dataset used, after imputation: distribution of women across all 'races' and 'ethnicities', by age group (split age: 50 years) and by diagnosis within 1 year from the mammogram screening.

Table S4. *[in separate file]* ER-positive predicted proportions by age at cancer diagnosis and ethnicity, in PV carriers and in the general population.

Table S5. *[in separate file]* ER-negative non-TN predicted proportions by age at cancer diagnosis and ethnicity, in PV carriers and in the general population.

Table S6. *[in separate file]* TN predicted proportions by age at cancer diagnosis and ethnicity, in PV carriers and in the general population.

Table S7. *[in separate file]* SNPs and weights included in the construction of the PGS models. There are three surrogates correlated with original SNP from Mavaddat et al. 2019 at r2>0.9). The 307-SNP PGS does not include 22_29203724_C_T and 22_29551872_A_G; the 303-SNP PGS also excludes in addition SNPs 6_27425644_G_C, 15_46680811_C_A, 3_29294845_C_T, and 11_46318032_C_G. [a]SNP variant name; [b]Position based on build 37.

Table S8. *[in separate file]* Allele frequencies (EAFs) by genetic ancestry for SNPs included in the PGS models. EAFs were estimated in UK Biobank individuals without a diagnosis of cancer or mastectomy prior to the start of follow-up. Genetic ancestry groupings are according to iadmix, derived as described in the Methods: EUR, European; AFR, African; EAS, East Asian; SAS, South Asian. [a]SNP variant name.

Table S9. *[in separate file]* Mean and standard deviation (SD) of the 309-SNP BC PGS in UK Biobank individuals without a diagnosis of cancer or mastectomy prior to the start of follow-up. Genetic ancestry groupings are according to iadmix, derived as described in the Methods; self-reported ethnicity groupings are as described in the Methods.

Table S10. *[in separate file]* Mean and standard deviation (SD) of the 307-SNP BC PGS in UK Biobank individuals without a diagnosis of cancer or mastectomy prior to the start of follow-up. Genetic ancestry groupings are according to iadmix, derived as described in the Methods; self-reported ethnicity groupings are as described in the Methods. The 307-SNP PGS does not include 22_29203724_C_T and 22_29551872_A_G.

Table S11. *[in separate file]* Self-reported ethnicity vs genetic ancestry (iadmix) in UK Biobank for individuals without a diagnosis of cancer. Genetic ancestry groupings are according to iadmix, derived as described in the Methods: EUR, European; AFR, African; EAS, East Asian; SAS, South Asian. Self-reported ethnicity groupings are as described in the Methods. In total, 226340 women, 200073 men and 426413 men and women were included in each analysis.

Table S12. *[in separate file]* SNPs, weights and allele frequencies (EAFs) by genetic ancestry included in the construction of the PGS model. EAFs were estimated in UK Biobank individuals without a diagnosis of cancer prior to the start of follow-up. SNPs log-OR were derived from OCAC published data. Genetic ancestry groupings are according to iadmix, derived as described in the Methods. [a]SNP variant name; [b]Position based on build 37.

Table S13. *[in separate file]* Mean, standard deviation (SD) and *alpha* parameters of the 36-SNP OC PGS. Mean and SD estimated in UK Biobank individuals without a diagnosis of cancer prior to the start of follow-up. α parameter estimated using SNPs frequencies from UK Biobank and SNPs log-OR from OCAC published data. Genetic ancestry groupings are according to iadmix, derived as described in the Methods.

Table S14. *[in separate file]* Mean, standard deviation (SD) and *alpha* parameters for BC PGS models. For Asians, parameters estimated using data from the MyBrCa and SGBCC studies. For Europeans, parameters based on the prospective and validation sets from Mavaddat *et al.*, 2019 , and the published procedure from Mavaddat *et al.*, 2023. [a]Number of SNPs included in PGS models: all 313 SNPs from Mavaddat *et al.*, 2019 (313-SNP PGS), UKB SNPs (309-SNP PGS), UKB SNPs excluding two CHEK2 SNPs (307-SNP PGS), no CHEK2 variants and imputation accuracy r2>0.5 (303-SNP PGS). [b]αGLM is based on logistic regression adjusted for country in which studies were conducted and principal components. [c]αRL is based on retrospective likelihood method as outlined in the Methods. [d]Results based on 6394 controls and 6150 cases among Chinese and Malay women classified as 'East-Asian' according to genetic data, with known age and age less than 80. [e]Results based on 1000 controls and 568 cases among Indian women classified as 'South-Asian' according to genetic data, with known age and age less than 80. [f]Results based on 22767 controls and 16151 cases; European women with known age and age less than 80.

Table S15. *[in separate file]* Statistics on the MyBrCa and SGBCC studies; for cases and controls, number of individuals and mean/standard deviation (SD) age at diagnosis/interview (years).

Table S16. Summary of published associations between breast cancer and rare pathogenic variants by different ancestries. Odds ratios [95% confidence intervals] were reported.

Table S17. Summary of published pathogenic variant frequencies (%) in the population by different ancestries without large rearrangement sensitivity adjustment.

Table S18. Estimated QRFs distributions in UK Biobank and MD distribution from BCSC. QRFs: questionnaire-based risk factors; MD: mammographic density; HRT: hormone replacement therapy; OC: oral contraceptive.

Table S19. Odds ratios [95% confidence intervals] for different BIRADS categories, estimated for each ethnicity separately. BIRADS B is the reference category for individuals of age less than 50 years (upper panel); BIRADS A is the reference category for individuals of age 50 years and over (lower panel).

Table S20. Ethnicity-specific odds ratio estimates [95% confidence intervals], using entire BCSC population and considering an interaction term between MD and ethnicity. BIRADS B is the reference category for individuals of age less than 50 years (upper panel); BIRADS A is the reference category for individuals of age 50 years and over (lower panel).

Table S21. Proportion [95% confidence intervals] of BIRADS categories in different ethnic groups, for individuals of age less than 50 years (upper panel) and for individuals of age 50 years and over (lower panel).

Table S22. Source of the model parameters for each ethnic/ancestry group (as described in this manuscript). 'default' indicates parameters in previous BOADICEA models (BC v6, EOC v2).

Table S23. Proportion of women falling in different risk categories depending on their lifetime cancer risk (age 20 to 80 years) and their ethnicity, using different risk predictors. Unknown genetic background, mother with BC/EOC at 50y. First panel: lifetime BC risk; the thresholds between risk categories are 17%, between 'population' and 'moderate', and 30%, between 'moderate' and 'high' (NICE guidelines). Second panel: lifetime EOC risk, with thresholds at 3.5% and 5% (threshold for risk-reducing surgery, NICE guidelines).

Table S24. Proportion of women falling in different risk categories depending on their 10-year BC risk (age 40 to 50 years) and their ethnicity, using different risk predictors. Unknown genetic background, unknown FH. The thresholds between risk categories are 3%, between 'population' and 'moderate', and 8%, between 'moderate' and 'high' (NICE guidelines).

Table S25. Proportion of women falling in different risk categories depending on their 10-year BC risk (age 40 to 50 years) and their ethnicity, using different risk predictors. Unknown genetic background, mother with BC at 50y. The thresholds between risk categories are 3%, between 'population' and 'moderate', and 8%, between 'moderate' and 'high' (NICE guidelines).

Figures

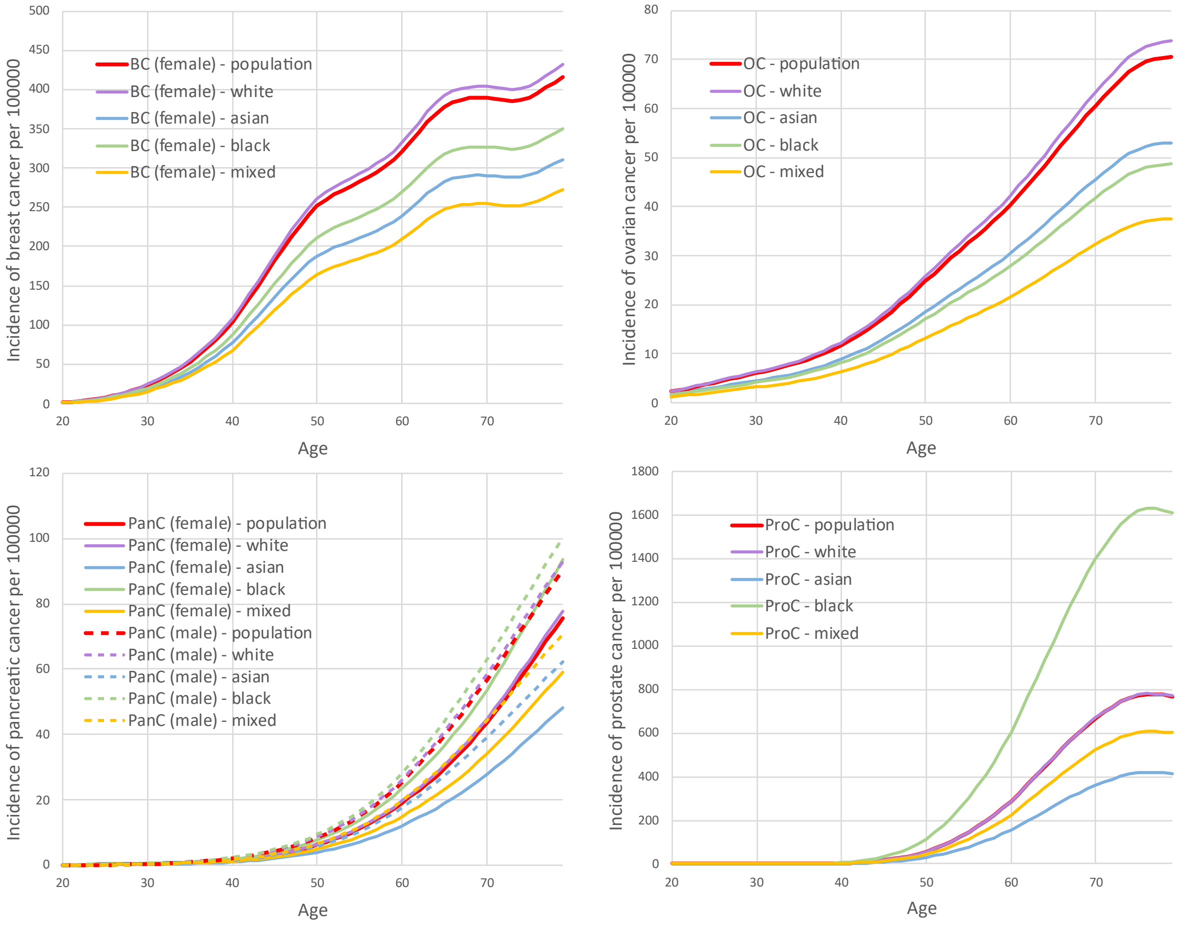

Figure S1. Ethnicity-specific cancer incidences per 100,000 people in the UK, employed in the BC and EOC models (1980-1989 birth cohort). Panel A, breast cancer (BC); panel B, ovarian cancer (OC); panel C, pancreatic cancer (PanC); panel D, prostate cancer (ProC).

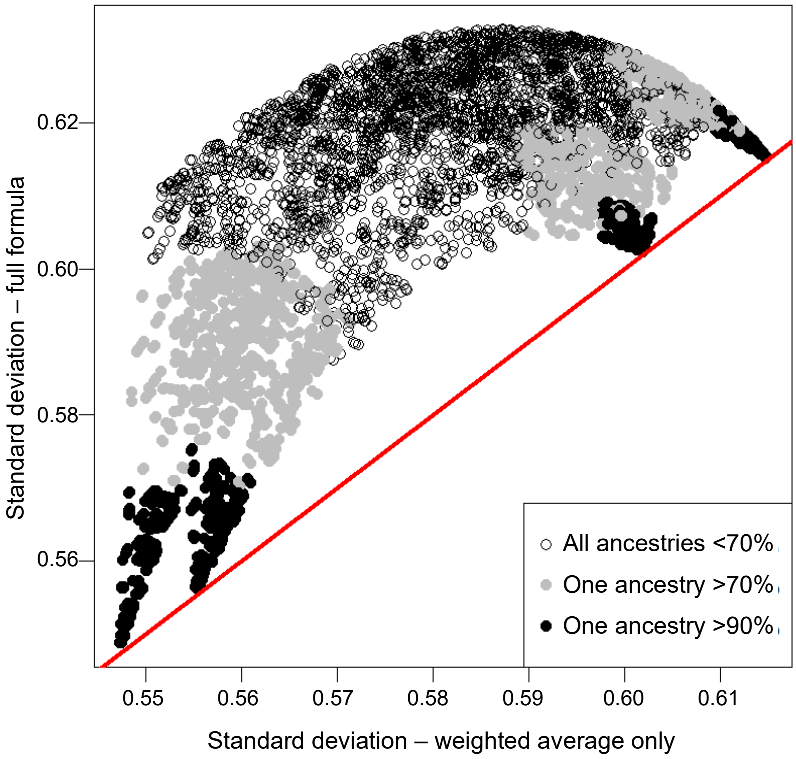

Figure S8. Standard deviations (SDs) for multiple (random) combinations of ancestry proportions, calculated using the full formula detailed in Methods and by weighted averaging the ancestry-specific SDs. Each random combination is created by successive extraction of ancestry proportions from continuous uniform distributions: the first proportion (P_1) is extracted from $U(0,1)$; the second one (P_2) from $U(0,1-P_1)$; the third one (P_3) from $U(0,1-P_1-P_2)$; the fourth one is set to $P_4 = 1 - P_1 - P_2 - P_3$

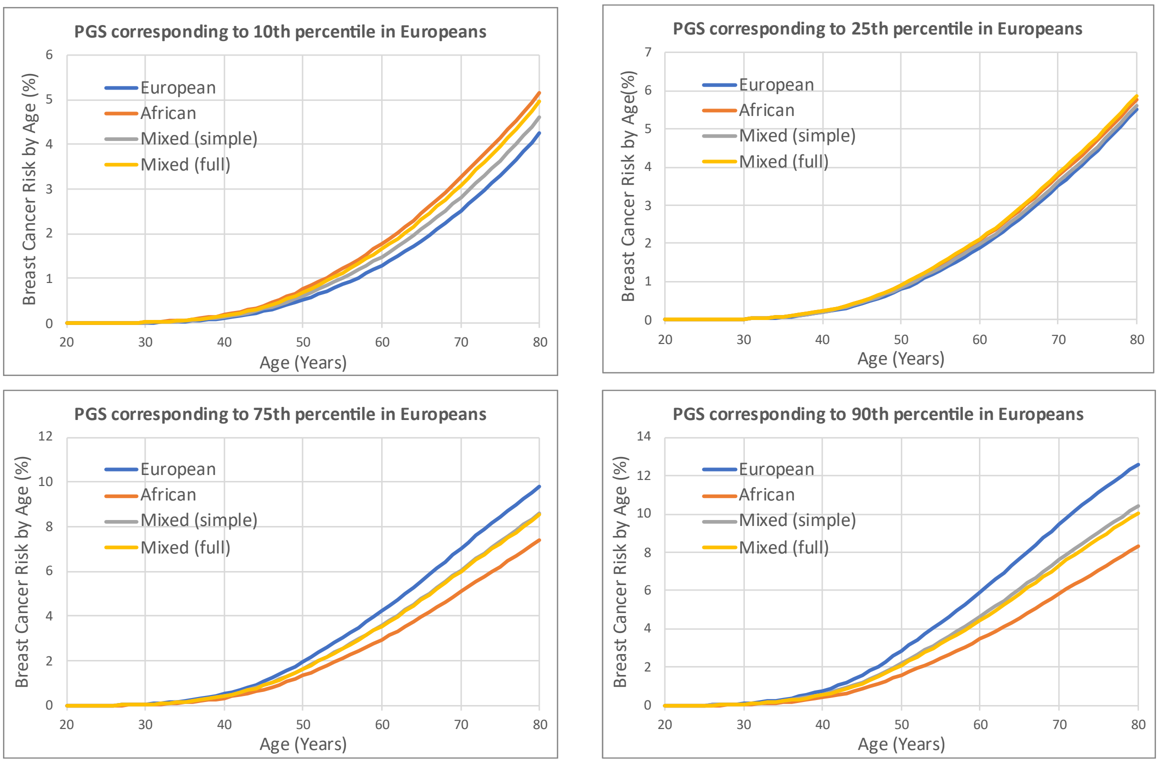

Figure S9. Predicted breast cancer risks by age for a female born in 2000 (Mixed ethnicity, unknown family history and QRFs), based on ancestry and PGS. Ancestry-specific standardised PGS were calculated from the same raw PGS values, corresponding to 10th, 25th, 45th, 55th, 75th and 90th percentile of the standardised PGS distribution in Europeans. 'Mixed (simple)' was obtained with a simplified formula for calculating σ ($\boldsymbol{\sigma^2 = \sum_{i=1}^{M} P_i \sigma_i^2}$); 'Mixed (full)' was obtained with the complete formula for σ.

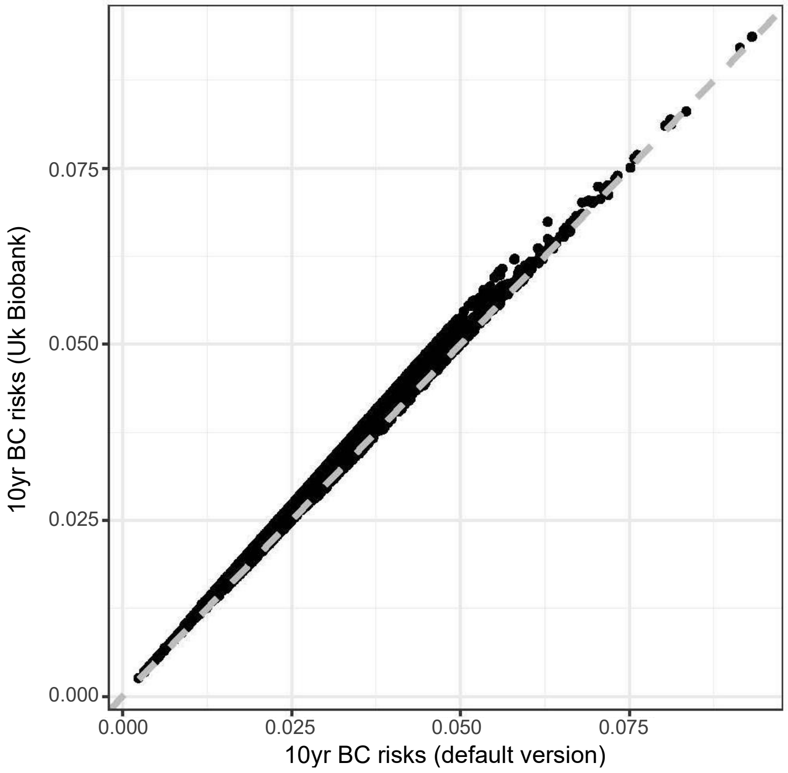

Figure S10. Comparison of 10-year breast cancer (BC) risk predictions in the KARMA cohort. X-axis shows the BC risks predicted by using the default QRFs distributions in BOADICEA whereas y-axis shows the BC risks predicted by switching to use QRFs distributions estimated in the UK Biobank White populations. The dashed line is the diagonal line with slope equal to 1 (corresponding to no difference in the risk predictions).

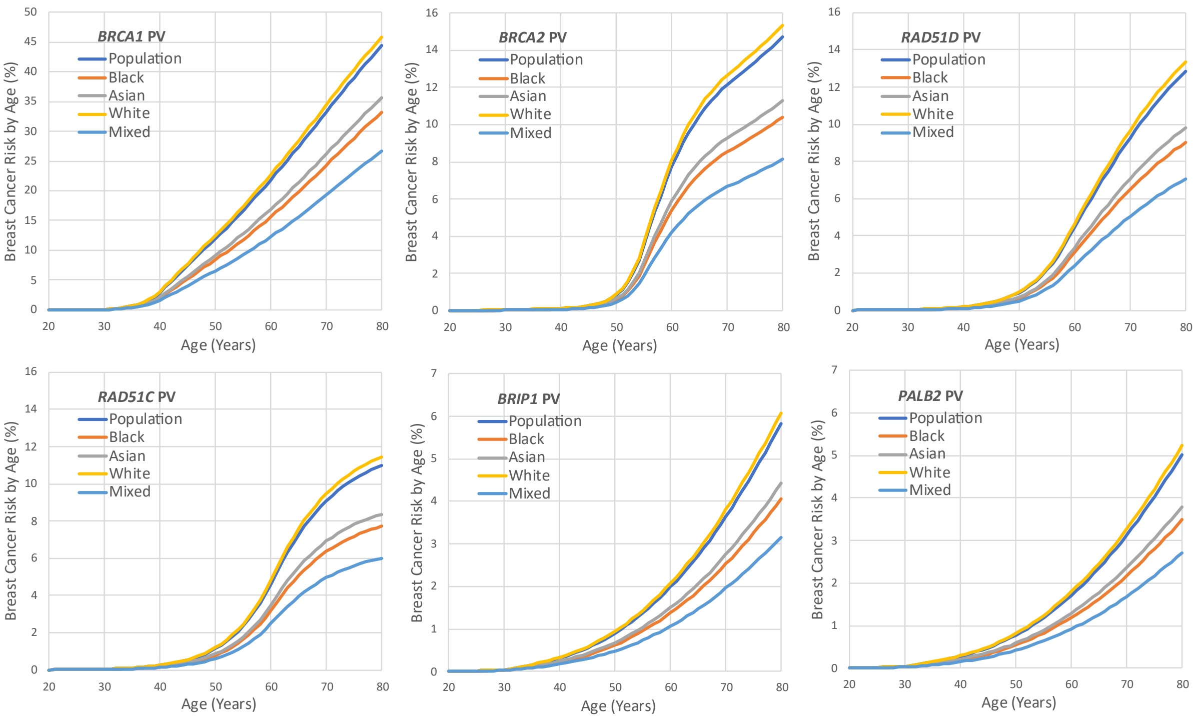

Figure S11. Predicted epithelial ovarian cancer risks by age for a female born in 1985 (unknown family history and QRFs) based on ethnicity and pathogenic variant carrier status (for the major genes considered in the EOC model).

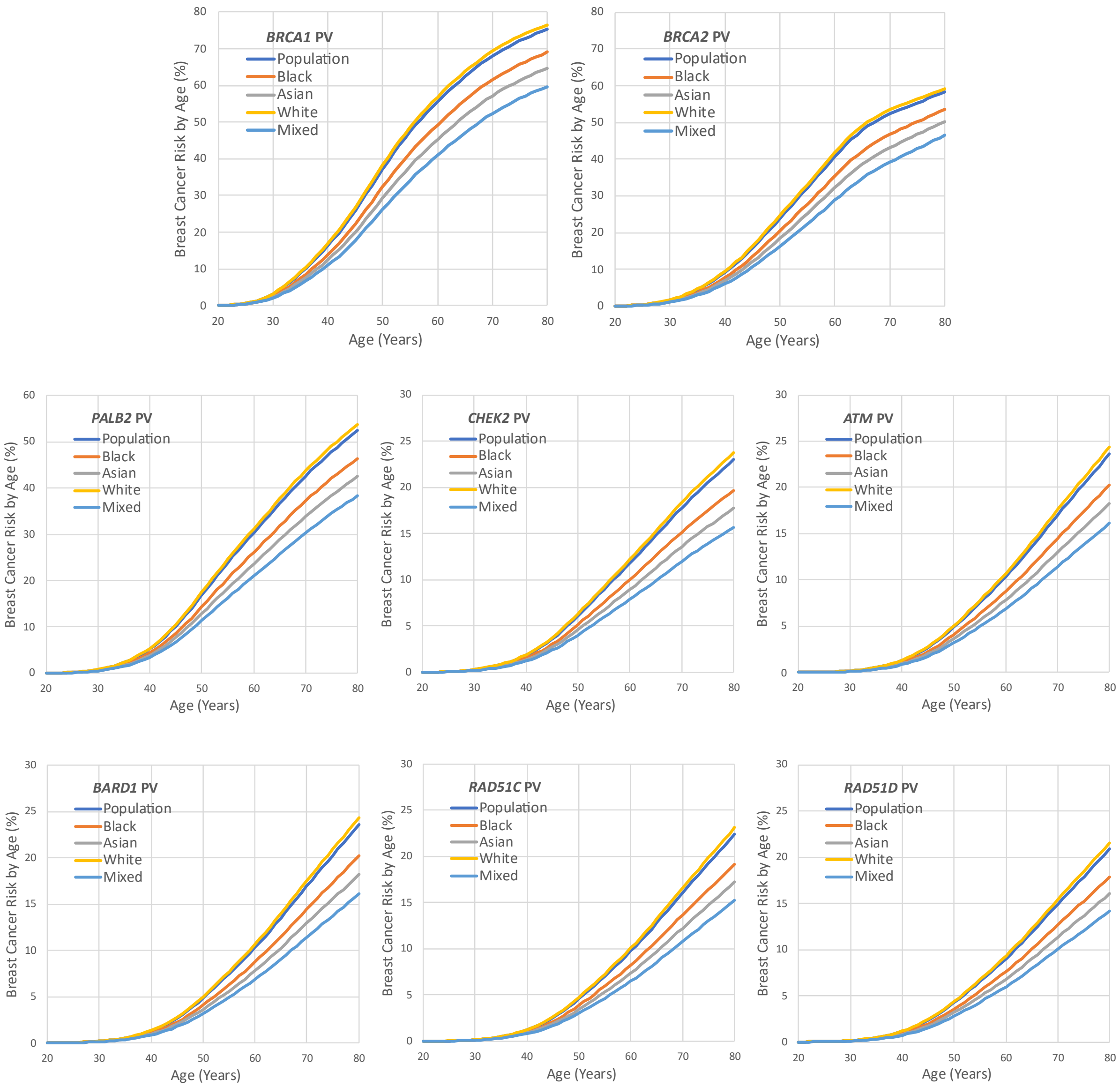

Figure S12. Predicted breast cancer risks by age for a female born in 1985 (unknown family history and QRFs) based on ethnicity and pathogenic variant carrier status (for the major genes considered in the BC model).

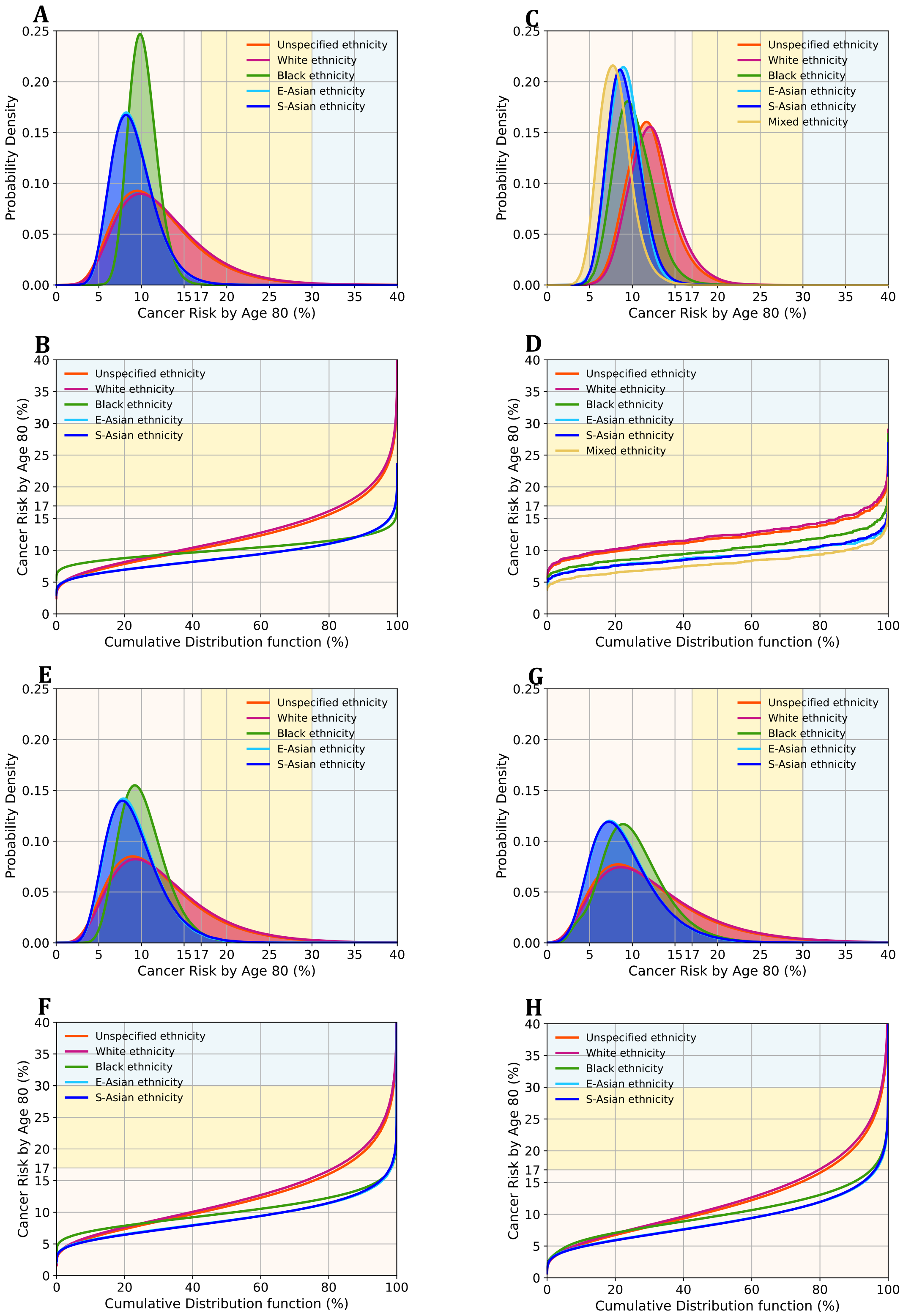

Figure S13. Predicted lifetime (age 20-80 years) BC risks for women of different ethnicities, untested for PVs and unknown family history. Risks are based on different combinations of risk predictors, each drawn in a separate panel: (A,B) PGS only; (C,D) QRFs only; (E,F) PGS and QRFs; (G,H) PGS and QRFs and MD. (A,C,E,G) Probability density function against absolute risk; (B,D,F,H) absolute risk against cumulative distribution. BC, breast cancer; PGS, polygenic score; QRFs, questionnaire-based risk factors; MD, mammographic density. The backgrounds of the graphs are shaded to indicate three lifetime BC risk categories: less than 17% (light yellow); between 17% and 30% (yellow); above 30% (light blue).

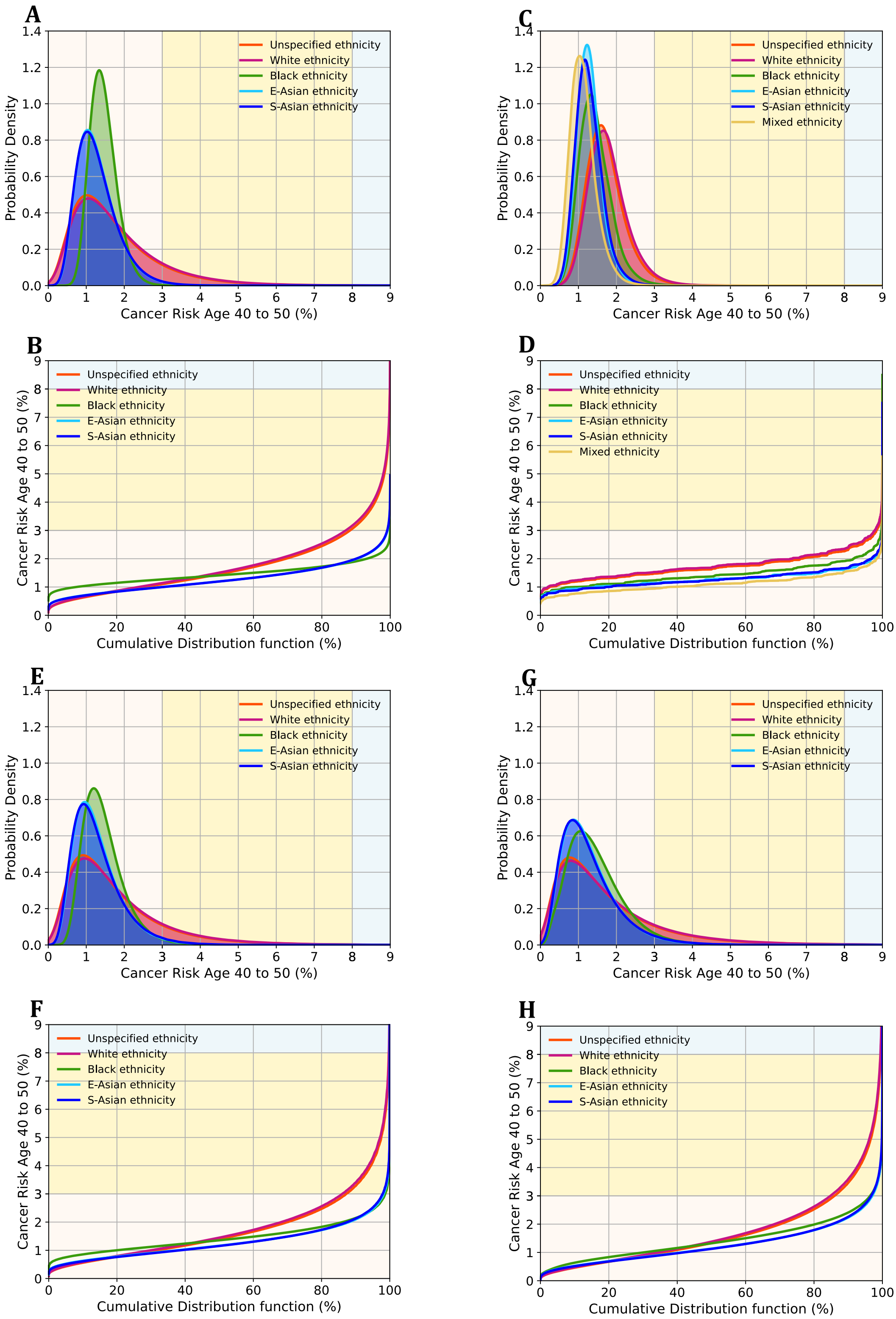

Figure S14. Predicted 10-year (to age 50 years) BC risks for women of different ethnicities, untested for PVs and unknown family history. Risks are based on different combinations of risk predictors, each drawn in a separate panel: (A,B) PGS only; (C,D) QRFs only; (E,F) PGS and QRFs; (G,H) PGS and QRFs and MD. (A,C,E,G) Probability density function against absolute risk; (B,D,F,H) absolute risk against cumulative distribution. BC, breast cancer; PGS, polygenic score; QRFs, questionnaire-based risk factors; MD, mammographic density. The backgrounds of the graphs are shaded to indicate three 10-year BC risk categories: less than 3% (light yellow); between 3% and 8% (yellow); above 8% (light blue).

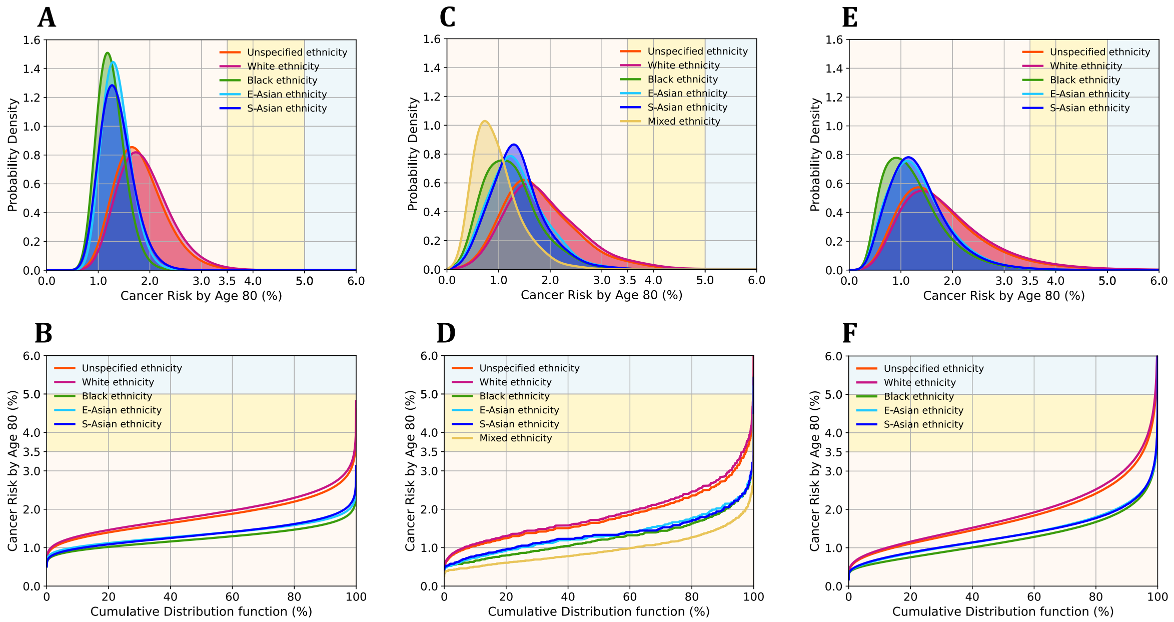

Figure S15. Predicted lifetime (age 20-80 years) EOC risks for women of different ethnicities, untested for PVs and unknown family history. Risks are based on different combinations of risk predictors, each drawn in a separate panel: (A,B) PGS only; (C,D) QRFs only; (E,F) PGS and QRFs. (A,C,E) Probability density function against absolute risk; (B,D,F) absolute risk against cumulative distribution. EOC, epithelial ovarian cancer; PGS, polygenic score; QRFs, questionnaire-based risk factors. The backgrounds of the graphs are shaded to indicate three lifetime EOC risk categories: less than 3.5% (light yellow); between 3.5% and 5% (yellow); above 5% (light blue).

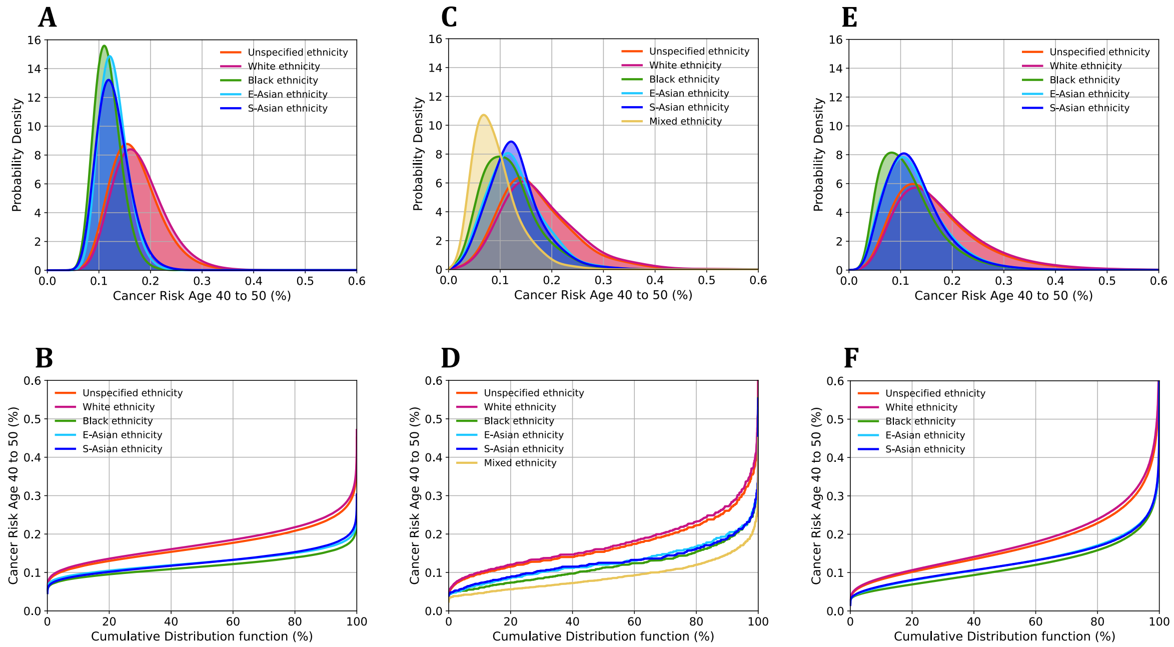

Figure S16. Predicted 10-year (to age 50 years) EOC risks for women of different ethnicities, untested for PVs and unknown family history. Risks are based on different combinations of risk predictors, each drawn in a separate panel: (A,B) PGS only; (C,D) QRFs only; (E,F) PGS and QRFs. (A,C,E) Probability density function against absolute risk; (B,D,F) absolute risk against cumulative distribution. EOC, epithelial ovarian cancer; PGS, polygenic score; QRFs, questionnaire-based risk factors.

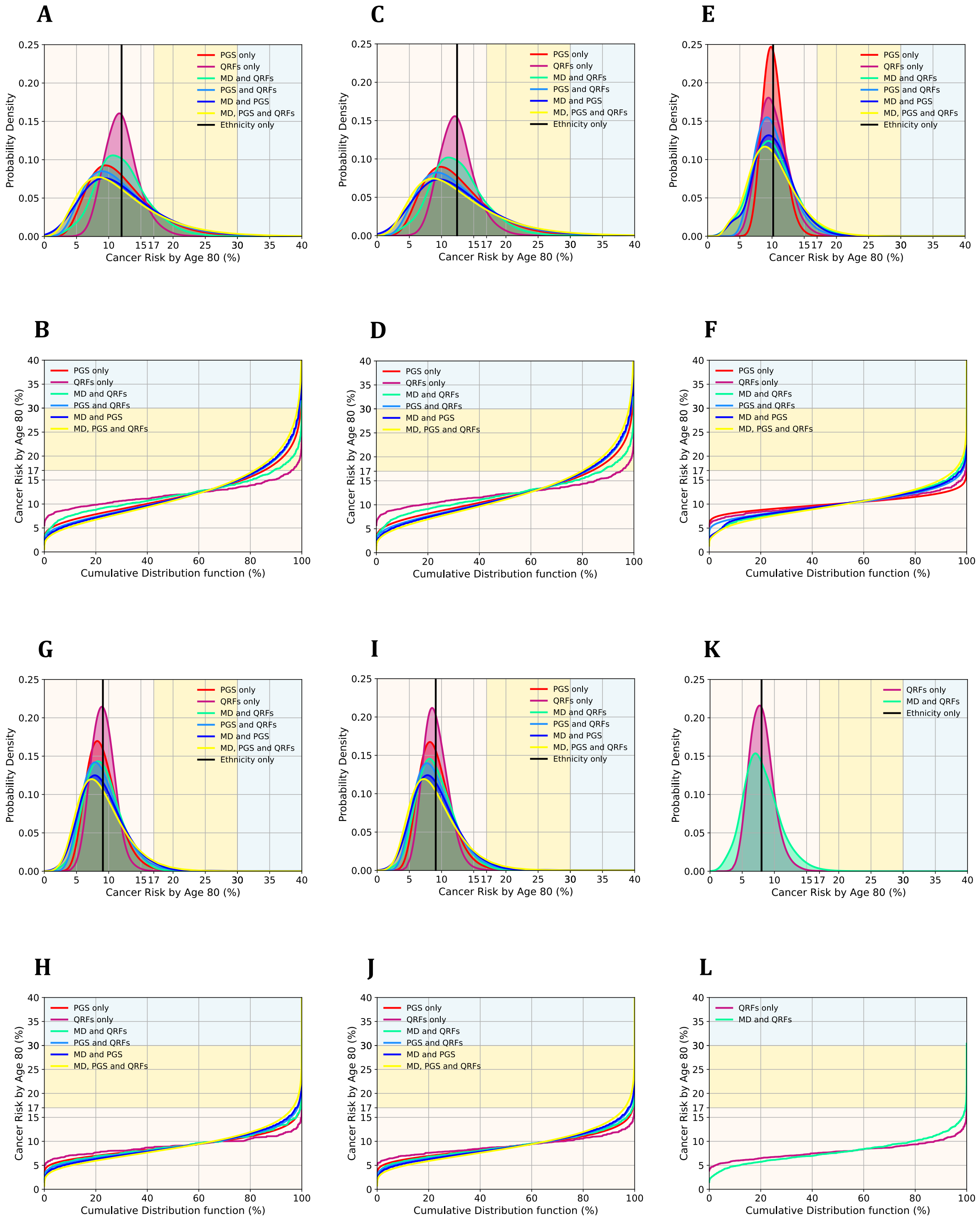

Figure S17. Predicted lifetime (age 20-80 years) BC risks for women of different ethnicities, untested for PVs and unknown family history, based on different combinations of risk predictors. Distributions for each ethnicity are drawn in separate panels: (A,B) Population/Unspecified; (C,D) White; (E,F) Black; (G,H) East Asian; (I,J) South Asian; (K,L) Mixed. The straight lines (labelled 'ethnicity only') are equivalent to the "population" risk of BC for the specific ethnicity plotted. (A,C,E,G,I,K) Probability density function against absolute risk; (B,D,F,H,J,L) absolute risk against cumulative distribution. BC, breast cancer; PGS, Polygenic Score; QRFs, questionnaire-based risk factors; MD, mammographic density. The backgrounds of the graphs are shaded to indicate three lifetime BC risk categories: less than 17% (light yellow); between 17% and 30% (yellow); above 30% (light blue).

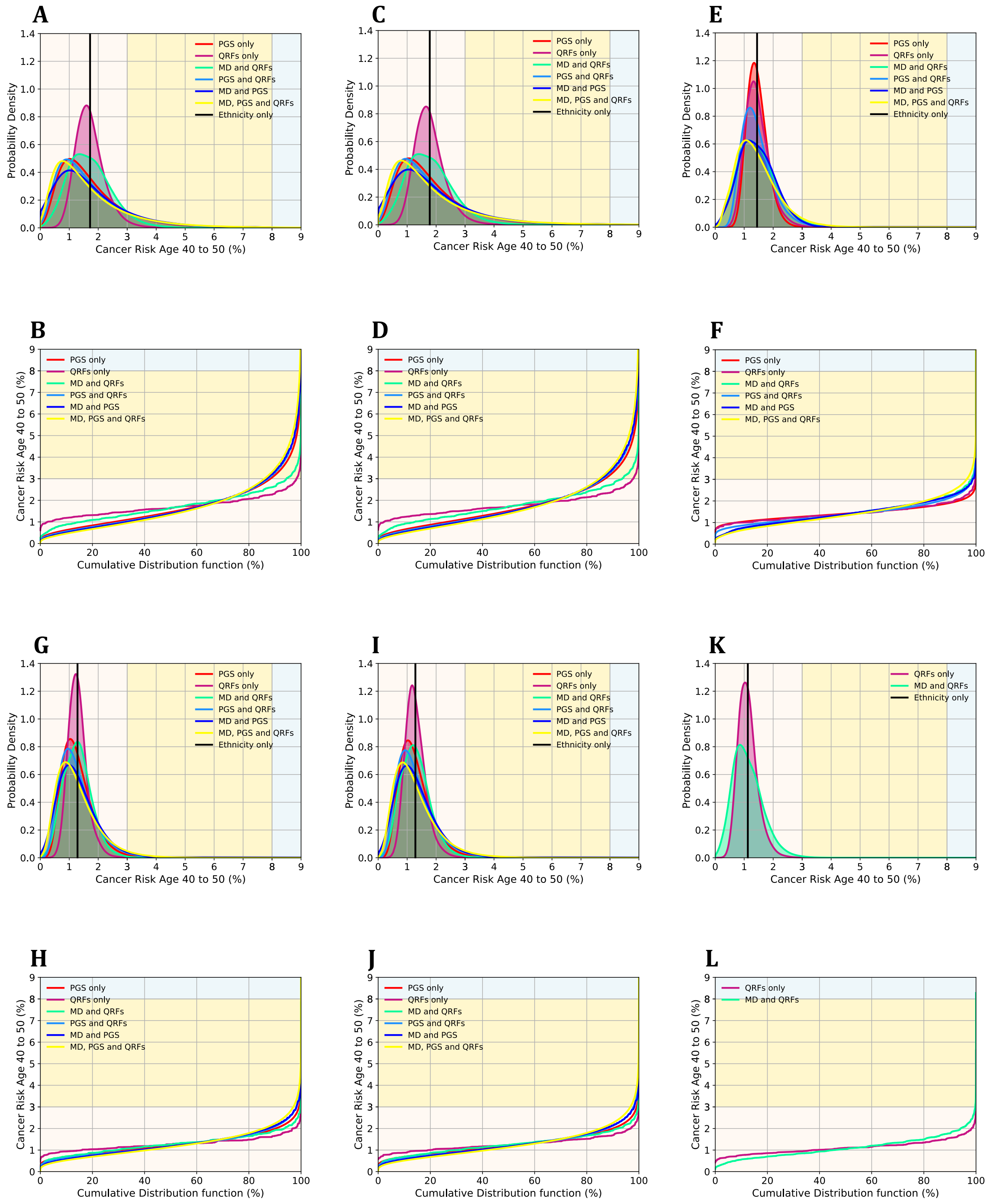

Figure S18. Predicted 10-year (to age 50 years) BC risks for women of different ethnicities, untested for PVs and unknown family history, based on different combinations of risk predictors. Distributions for each ethnicity are drawn in separate panels: (A,B) Population/Unspecified; (C,D) White; (E,F) Black; (G,H) East Asian; (I,J) South Asian; (K,L) Mixed. The straight lines (labelled 'ethnicity only') are equivalent to the "population" risk of BC for the specific plotted. (A,C,E,G,I,K) Probability density function against absolute risk; (B,D,F,H,J,L) absolute risk against cumulative distribution. BC, breast cancer; PGS, polygenic score; QRFs, questionnaire-based risk factors; MD, mammographic density. The backgrounds of the graphs are shaded to indicate three 10-year BC risk categories: less than 3% (light yellow); between 3% and 8% (yellow); above 8% (light blue).

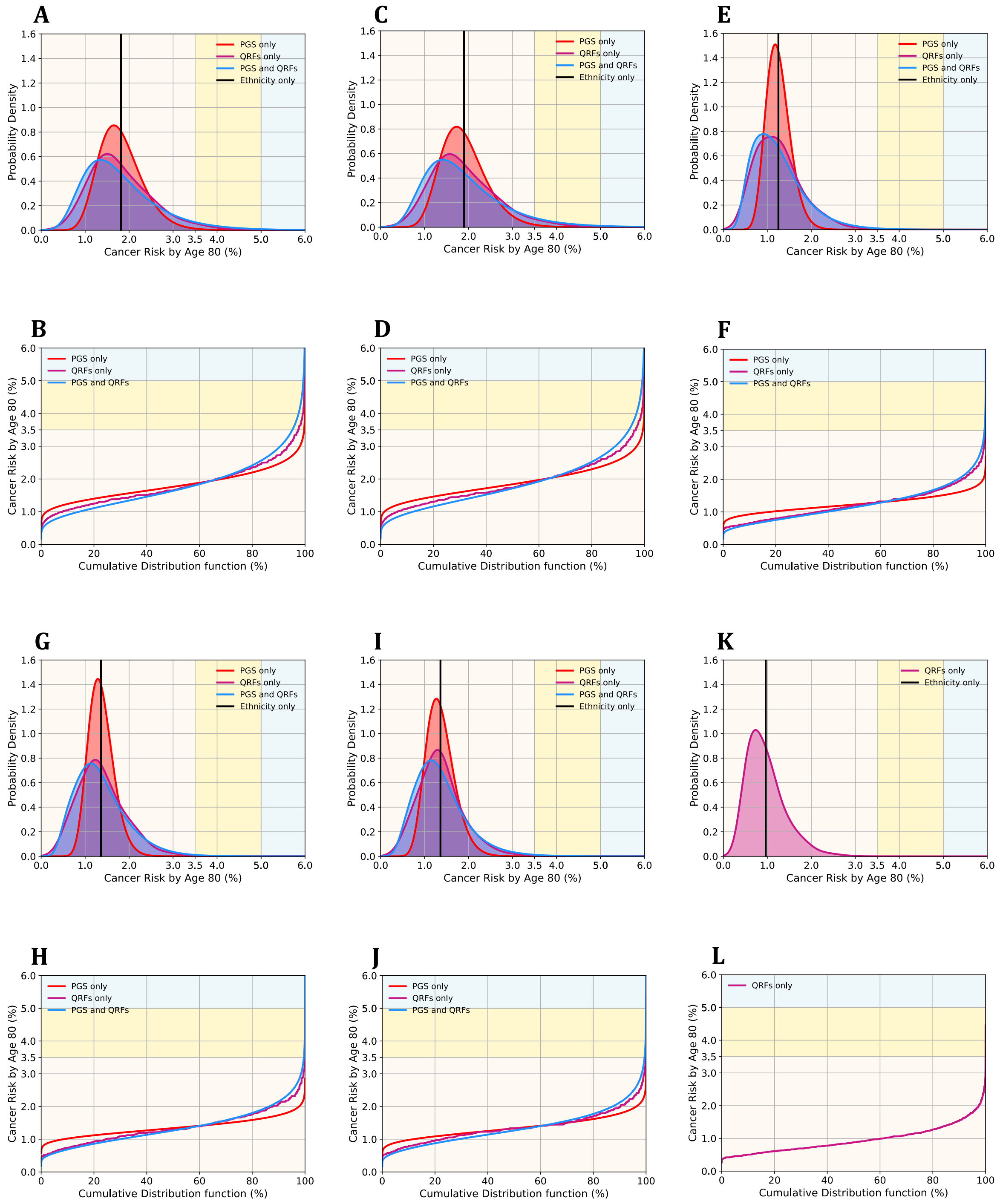

Figure S19. Predicted lifetime (age 20-80 years) EOC risks for women of different ethnicities, untested for PVs and unknown family history, based on different combinations of risk predictors. Distributions for each ethnicity are drawn in separate panels: (A,B) Population/Unspecified; (C,D) White; (E,F) Black; (G,H) East Asian; (I,J) South Asian; (K,L) Mixed. The straight lines (labelled 'ethnicity only') are equivalent to the "population" risk of EOC for the specific ethnicity plotted. (A,C,E,G,I,K) Probability density function against absolute risk; (B,D,F,H,J,L) absolute risk against cumulative distribution. EOC, epithelial ovarian cancer; PGS, polygenic score; QRFs, questionnaire-based risk factors. The backgrounds of the graphs are shaded to indicate three lifetime EOC risk categories: less than 3.5% (light yellow); between 3.5% and 5% (yellow); above 5% (light blue).

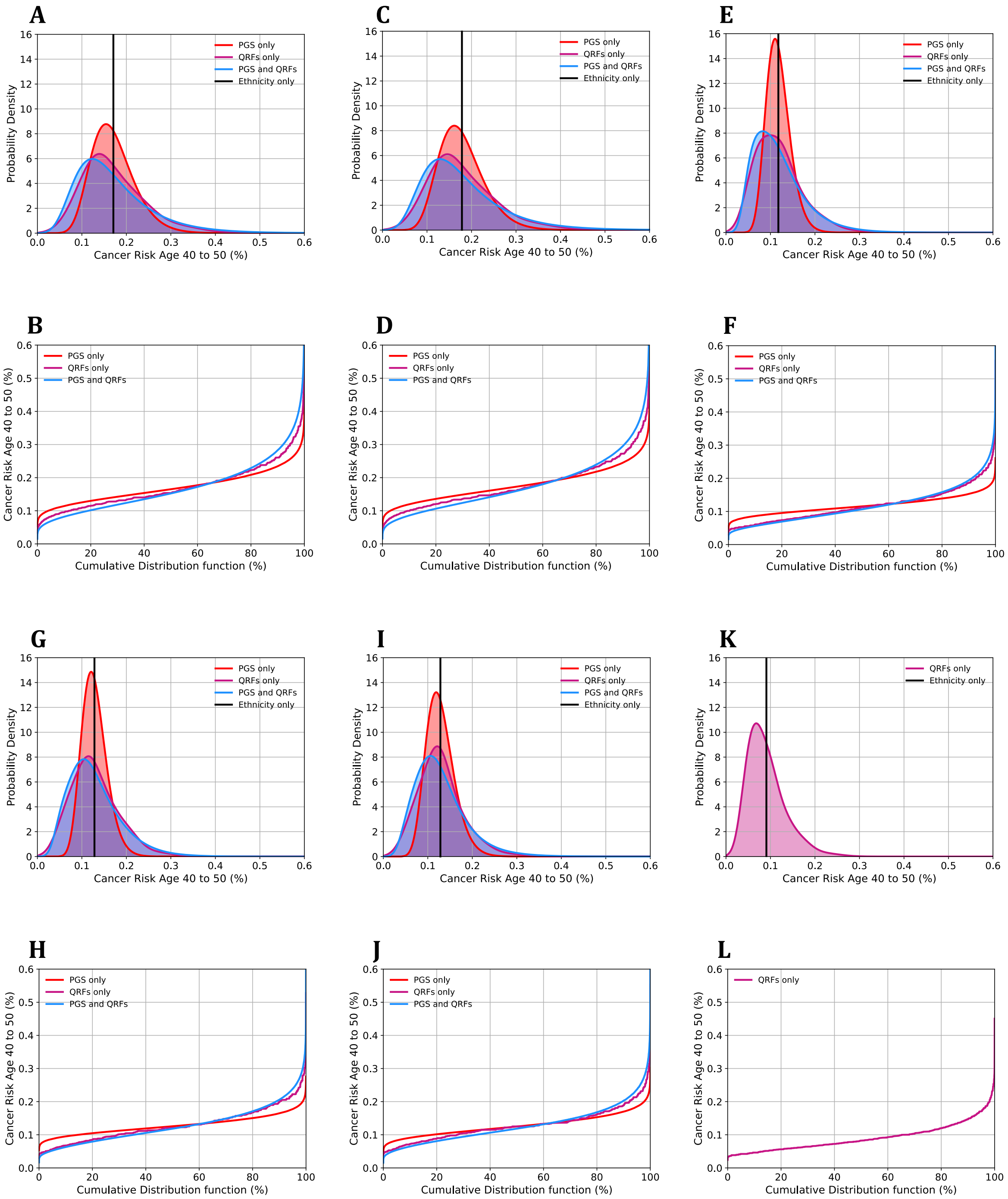

Figure S20. Predicted 10-year (to age 50 years) EOC risks for women of different ethnicities, untested for PVs and unknown family history, based on different combinations of risk predictors. Distributions for each ethnicity are drawn in separate panels: (A,B) Population/Unspecified; (C,D) White; (E,F) Black; (G,H) East Asian; (I,J) South Asian; (K,L) Mixed. The straight lines (labelled 'ethnicity only') are equivalent to the "population" risk of EOC for the specific ethnicity plotted. (A,C,E,G,I,K) Probability density function against absolute risk; (B,D,F,H,J,L) absolute risk against cumulative distribution. EOC, epithelial ovarian cancer; PGS, polygenic score; QRFs, questionnaire-based risk factors.

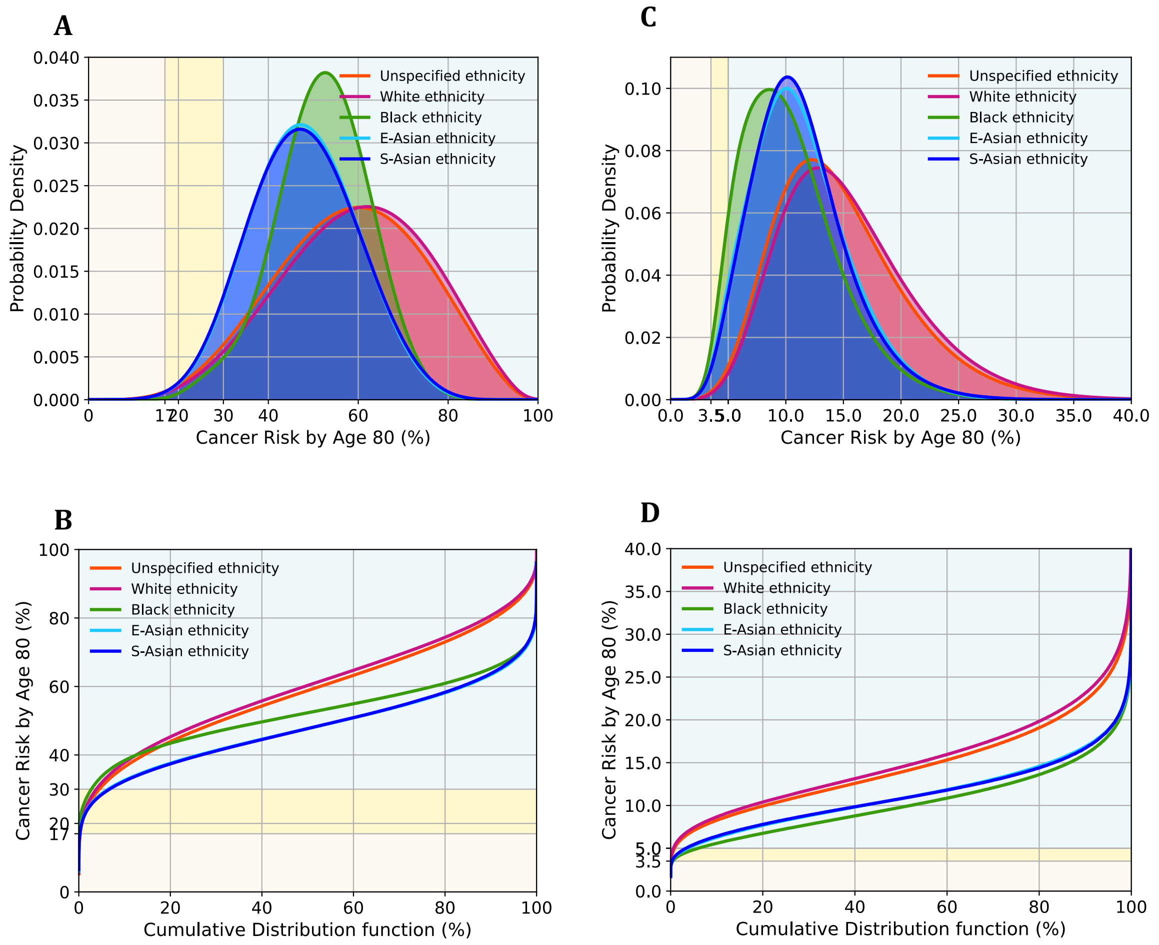

Figure S21. Predicted BC and EOC lifetime (age 20-80 years) risks for women of different ethnicities, *BRCA2* PV carrier and unknown family history; risks calculated using all risk predictors: PGS, QRFs, and MD. (A,B) BC risk; backgrounds are shaded to indicate three risk categories: <17% (light yellow); 17% - 30% (yellow); ≥ 30% (light blue). (C,D) EOC risk; backgrounds are shaded to indicate three risk categories: < 3.5% (light yellow); 3.5% - 5% (yellow); ≥ 5% (light blue). (A,C) probability density function against absolute risk; (B,D) absolute risk against cumulative distribution. BC, breast cancer; EOC, epithelial ovarian cancer; PGS, polygenic score; QRFs, questionnaire-based risk factors; MD, mammographic density.

Tables

Table S1. Ethnicity proportions ($\boldsymbol{P_{ethnicity}}$) obtained from the ONS (22) and incidence rate ratios by ethnic group and cancer type ($\boldsymbol{R_{ethnicity}}$), relative to White (21). East and South Asian ethnicities were grouped in a single group due to small numbers.

**Ethnicities**	*White*	*Black*	*Asian*	*Mixed*	*Other*
Proportion (2016 census)	0.851	0.035	0.078	0.0176	0.018
Breast cancer rate ratio (women)	1.00	0.81	0.72	0.63	NA
Ovarian cancer rate ratio	1.00	0.66	0.72	0.51	NA
Prostate cancer rate ratio	1.00	2.10	0.54	0.78	NA
Pancreatic cancer rate ratio (women)	1.00	1.20	0.62	0.76	NA
Pancreatic cancer rate ratio (men)	1.00	1.09	0.67	0.76	NA

Table S2. Summary of the BCSC dataset used: distribution of women across all 'races' and 'ethnicities', by age group (split age: 50 years) and by diagnosis within 1 year from the mammogram screening.

**'Race', 'ethnicity'**	**Age <50 years**		**Age >=50 years**	
	All women	*BC within 1y*	*All women*	*BC within 1y*
White, non-Hispanic	170520	523	240195	1913
Black, non-Hispanic	15310	37	15861	103
Asian, non-Hispanic	11648	20	16180	61
Mixed, non-Hispanic	4413	5	6950	26
Unknown, non-Hispanic	7342	23	11294	60
White, Hispanic	18103	54	29456	156
Black, Hispanic	89	0	74	1
Asian, Hispanic	167	0	241	3
Mixed, Hispanic	1470	4	1704	5
Unknown, Hispanic	4174	10	4515	19
White, unknown	15971	33	21222	144
Black, unknown	472	0	886	6
Asian, unknown	2259	8	5674	27
Mixed, unknown	2154	5	4415	6
Unknown, unknown	39425	120	66705	449

Table S3. Summary of the BCSC dataset used, after imputation: distribution of women across all 'races' and 'ethnicities', by age group (split age: 50 years) and by diagnosis within 1 year from the mammogram screening.

'Race', 'ethnicity'	Age <50 years		Age >=50 years	
	All women	*BC within 1y*	*All women*	*BC within 1y*
White, non-Hispanic	184793	553	258990	2041
Black, non-Hispanic	15732	37	16649	109
Asian, non-Hispanic	13680	27	21215	85
Mixed, non-Hispanic	6319	10	10837	32
Unknown, non-Hispanic	42602	130	70544	461
White, Hispanic	19801	57	31883	172
Black, Hispanic	139	0	172	1
Asian, Hispanic	394	1	880	6
Mixed, Hispanic	1718	4	2232	6
Unknown, Hispanic	8339	23	11970	67

Table S16. Summary of published associations between breast cancer and rare pathogenic variants by different ancestries. Odds ratios [95% confidence intervals] were reported.

	**European ancestry**	**Asian ancestry**			**African ancestry**	
Data source	*BRIDGES (31)*	*BRIDGES (31)*	*Japanese study (35)*	*Asian families (36)*	*GBHS (33)*	*Afr. Am. study (34)*
***Cases* #**	42060	6279	7051	572 families	871	5054
***Controls* #**	44035	6047	11241		1563	4993
BRCA1	9.3 [7.0-12.4]	22.1 [6.9-70.5]	33.0 [13.7-103.8]	15.6 [12.6-19.4]	13.7 [4.0-46.5]	47.6 [10.4-100]
BRCA2	5.4 [4.4-6.6]	8.2 [4.9-13.6]	16.4 [10.2-28.0]	10.3 [8.3-12.7]	7.0 [3.2-15.5]	7.3 [4.1-14.1]
PALB2	5.0 [3.6-6.9]	4.5 [2.0-10.2]	9.0 [3.4-29.7]	NA	17.3 [2.2-138.1]	8.5 [3.7-25.0]
ATM	2.1 [1.7-2.6]	2.3 [1.2-4.8]	2.1 [1.0-4.1]	NA	1.6 [0.4-6.1]	1.8 [1.0-3.4]
CHEK2	2.6 [2.2-3.0]	1.5 [0.6-3.8]	3.2 [1.6-6.8]	NA	NA	NA
BARD1	2.0 [1.3-3.2]	2.5 [0.8-8.0]	NA	NA	NA	0.8 [0.3-2.3]
RAD51C	1.9 [1.1-3.3]	2.0 [0.8-5.0]	NA	NA	2.8 [0.2-45.7]	3.0 [0.9-13.9]
RAD51D	1.7 [0.9-2.9]	2.3 [0.9-5.9]	NA	NA	NA	2.9 [0.7-19.9]

Table S17. Summary of published pathogenic variant frequencies (%) in the population by different ancestries without large rearrangement sensitivity adjustment.

	European ancestry		Asian ancestry			African ancestry	
Data source	*BRIDGES (31)*	*CARRIERS (32)*	*BRIDGES (31)*	*CARRIERS (32)*	*Japanese (35)*	*GBHS (33)*	*CARRIERS (32)*
Sample size	44035	24770	6047	1269	11241	1563	4954
BRCA1	0.06	0.14	0.02	0	0.04	0.19	0.02
BRCA2	0.13	0.23	0.14	0.39	0.17	0.51	0.24
PALB2	0.05	0.13	0.06	0.18	0.04	0.06	0.10
ATM	0.16	0.42	0.09	0.39	0.15	0.32	0.34
CHEK2	0.35	0.50	0.07	0.24	0.12	0	0.14
BARD1	0.03	0.10	0.03	0.08	NA	0	0.16
RAD51C	0.02	0.10	0.06	0.16	NA	0.06	0.08
RAD51D	0.02	0.04	0.05	0	NA	0	0.06

Table S18. Estimated QRFs distributions in UK Biobank and MD distribution from BCSC. QRFs: questionnaire-based risk factors; MD: mammographic density; HRT: hormone replacement therapy; OC: oral contraceptive.

Risk factors	Category	White	Black	East Asian	South Asian	Mixed	BOADICEA (BC v6, EOC v2)
**Risk factors used in both breast and ovarian cancer risk prediction**							
**HRT use**	*Current C-type*	0.189	0.081	0.091	0.09	0.134	0.076
	Current E-type	0.001	0.001	0.001	0	0	0.011
	Former	0.203	0.09	0.094	0.111	0.113	0.913
	Never	0.607	0.828	0.814	0.799	0.752	
**Height**	*[0,153)*	0.05	0.059	0.197	0.242	0.071	0.0625
	[153,160)	0.258	0.271	0.442	0.438	0.28	0.2500
	[160,166)	0.376	0.373	0.279	0.25	0.366	0.3750
	[166,173)	0.258	0.242	0.077	0.065	0.23	0.2500
	>=173	0.058	0.056	0.004	0.005	0.052	0.0625
	Mean	162.63	162.36	157.49	156.71	161.97	162.81
	SD	6.24	6.30	5.59	5.93	6.39	6.45
**Parity**	*0*	0.188	0.167	0.222	0.119	0.243	0.28
	1	0.133	0.175	0.247	0.108	0.173	0.16
	2	0.445	0.263	0.375	0.377	0.34	0.33
	>=3	0.235	0.395	0.156	0.395	0.244	0.23
**Risk factors used in breast cancer risk prediction**							
**Age at menarche**	*<11*	0.046	0.058	0.038	0.049	0.068	0.122
	11	0.156	0.118	0.11	0.116	0.157	0.216
	12	0.190	0.183	0.252	0.163	0.193	0.268
	13	0.245	0.211	0.227	0.237	0.236	0.215
	14	0.198	0.169	0.185	0.193	0.177	0.115
	15	0.109	0.136	0.103	0.129	0.104	0.044
	>15	0.056	0.124	0.085	0.112	0.065	0.019
**Age at menopause**	*[0,40)*	0.04	0.062	0.027	0.045	0.058	0.022
	[40,45)	0.09	0.131	0.07	0.128	0.102	0.070
	[45,50)	0.236	0.339	0.24	0.338	0.301	0.234
	[50,55)	0.481	0.343	0.539	0.377	0.435	0.533
	>=55	0.152	0.126	0.124	0.112	0.104	0.14
**Age at first live birth**	*[0,20)*	0.097	0.292	0.041	0.115	0.209	0.1667
	[20,25)	0.347	0.344	0.222	0.422	0.308	0.3194
	[25,30)	0.379	0.243	0.406	0.321	0.29	0.3194
	>=30	0.177	0.122	0.331	0.143	0.193	0.1944
**MD (BIRADS 4th), age <50y**	*A*	0.044	0.057	*0.014*	*0.014*	0.049	0.041
	B	0.351	0.367	*0.226*	*0.226*	0.394	0.352
	C	0.466	0.447	*0.518*	*0.518*	0.445	0.470
	D	0.139	0.099	*0.242*	*0.242*	0.113	0.137
**MD (BIRADS 4th), age >=50y**	*A*	0.097	0.105	*0.108*	*0.108*	0.125	0.041
	B	0.491	0.502	*0.434*	*0.434*	0.542	0.352
	C	0.359	0.355	*0.368*	*0.368*	0.296	0.470
	D	0.053	0.038	*0.090*	*0.090*	0.037	0.137

**BMI (kg/m2)**	*[0,18.5)*	0.008	0.002	0.03	0.007	0.009	0.033
	[18.5,25)	0.394	0.182	0.687	0.319	0.405	0.357
	[25,30)	0.367	0.346	0.242	0.414	0.343	0.358
	>=30	0.231	0.47	0.041	0.26	0.243	0.252
**OC use**	*Current*	0.038	0.05	0.043	0.031	0.073	0.131
	Former	0.783	0.677	0.512	0.484	0.772	0.684
	Never	0.179	0.273	0.445	0.485	0.155	0.185
**Alcohol (g/day)**	*0*	0.102	0.335	0.488	0.672	0.170	0.106
	(0,5)	0.207	0.372	0.318	0.200	0.276	0.307
	[5,15)	0.400	0.205	0.134	0.093	0.320	0.276
	[15,25)	0.179	0.054	0.040	0.019	0.138	0.133
	[25,35)	0.067	0.021	0.013	0.008	0.050	0.080
	[35,45)	0.025	0.006	0.003	0.004	0.015	0.036
	>=45	0.020	0.008	0.003	0.003	0.031	0.061
**Risk factors used in ovarian cancer risk prediction**							
**Endometriosis**	*No*	0.985	0.988	0.990	0.991	0.980	0.90
	Yes	0.015	0.012	0.010	0.009	0.020	0.10
**Tubal ligation**	*No*	0.994	0.991	0.999	0.996	0.994	0.77
	Yes	0.006	0.009	0.001	0.004	0.006	0.23
**OC use duration**	*Never, <1*	0.234	0.367	0.532	0.572	0.222	0.710
	1-4	0.170	0.148	0.158	0.149	0.156	0.138
	5-9	0.200	0.149	0.109	0.114	0.189	0.086
	10-14	0.172	0.129	0.081	0.077	0.149	0.046
	≥15	0.224	0.208	0.121	0.088	0.284	0.020
**BMI**	*<22.5*	0.172	0.059	0.433	0.128	0.190	0.325
	22.5-<30	0.598	0.470	0.526	0.612	0.567	0.529
	≥30	0.231	0.470	0.041	0.260	0.243	0.146

Table S19. Odds ratios [95% confidence intervals] for different BIRADS categories, estimated for each ethnicity separately. BIRADS B is the reference category for individuals of age less than 50 years (upper panel); BIRADS A is the reference category for individuals of age 50 years and over (lower panel).

ORs	Category A	Category B	Category C	Category D
White, <50	0.48 [0.19-1.24]	1	1.23 [0.98-1.54]	1.45 [1.04-2.01]
Black, <50	0.75 [0.10-5.68]	1	1.17 [0.51-2.65]	1.76 [0.58-5.37]
Asian, <50	1.76 [0.27-11.40]	1	1.24 [0.31-5.01]	1.03 [0.19-5.58]
Mixed, <50	NA	1	1.06 [0.18-6.32]	1.35 [0.21-8.66]
White, >=50	1	1.39 [1.11-1.74]	1.76 [1.39-2.22]	1.79 [1.33-2.40]
Black, >=50	1	1.68 [0.65-4.37]	1.92 [0.640-5.735]	1.756 [0.47-6.59]
Asian, >=50	1	1.41 [0.53-3.74]	1.52 [0.444-5.220]	1.214 [0.21-6.98]
Mixed, >=50	1	1.35 [0.34-5.36]	1.51 [0.372-6.102]	NA

Table S20. Ethnicity-specific odds ratio estimates [95% confidence intervals], using entire BCSC population and considering an interaction term between MD and ethnicity. BIRADS B is the reference category for individuals of age less than 50 years (upper panel); BIRADS A is the reference category for individuals of age 50 years and over (lower panel).

ORs	Category A	Category B	Category C	Category D
White, <50	0.45 [0.19-1.35]	1	1.24 [0.99-1.54]	1.46 [1.03-2.04]
Black, <50	1.67 [0.18-17.22]	1	1.13 [0.33-4.03]	0.95 [0.20-4.55]
Asian, <50	1.12 [0.30-4.23]	1	1.79 [0.98-3.16]	2.08 [0.97-4.26]
Mixed, <50	0.67 [0.08-6.44]	1	1.16 [0.40-3.14]	1.62 [0.47-5.69]
White, >=50	1	1.38 [1.13-1.67]	1.72 [1.39-2.13]	1.75 [1.32-2.32]
Black, >=50	1	1.50 [0.50-4.50]	1.48 [0.48-4.61]	1.28 [0.24-6.76]
Asian, >=50	1	1.93 [1.13-3.31]	2.15 [1.25-3.72]	3.80 [1.98-7.27]
Mixed, >=50	1	1.62 [0.57-4.61]	1.82 [0.61-5.41]	1.83 [0.41-8.11]

Table S21. Proportion [95% confidence intervals] of BIRADS categories in different ethnic groups, for individuals of age less than 50 years (upper panel) and for individuals of age 50 years and over (lower panel).

Proportions	Category A	Category B	Category C	Category D
White, <50	0.044 [0.043-0.045]	0.351 [0.348-0.353]	0.466 [0.464-0.468]	0.139 [0.137-0.141]
Asian, <50	0.014 [0.011-0.017]	0.226 [0.216-0.236]	0.518 [0.506-0.529]	0.242 [0.232-0.252]
Black, <50	0.057 [0.053-0.060]	0.367 [0.360-0.374]	0.477 [0.470-0.484]	0.099 [0.095-0.104]
Mixed, <50	0.049 [0.042-0.056]	0.394 [0.377-0.410]	0.445 [0.428-0.461]	0.113 [0.102-0.123]
White, >=50	0.097 [0.096-0.098]	0.491 [0.489-0.493]	0.359 [0.357-0.360]	0.053 [0.052-0.054]
Asian, >=50	0.108 [0.103-0.113]	0.434 [0.426-0.442]	0.368 [0.361-0.376]	0.090 [0.085-0.094]
Black, >=50	0.105 [0.101-0.109]	0.502 [0.496-0.508]	0.355 [0.349-0.361]	0.038 [0.036-0.041]
Mixed, >=50	0.125 [0.117-0.134]	0.542 [0.530-0.555]	0.296 [0.285-0.307]	0.037 [0.032-0.041]

Table S22. Source of the model parameters for each ethnic/ancestry group (as described in this manuscript). *'default'* indicates parameters in previous BOADICEA models (BC v6, EOC v2).

**Ethnicity**	**CHEK2 PV frequencies**	**PGS parameters**	**MD distribution**	**Other QRFs distribution**	**ER and TN status**	**Incidences**
Source (section)	'Rare genetic variants'	Table 2	Table S18	Table S18	Tables S4-S6	Table 1
Unspecified	*default*	*default*	*default*	*default*	*default*	*default*
White	*default*	European	White	*default*	White	White
Black	non-European	African	Black	Black	Black	Black
East Asian	non-European	East Asian	Asian	East Asian	East Asian	Asian
South Asian	non-European	South Asian	Asian	South Asian	South Asian	Asian
Mixed	non-European	*(derived)*	Mixed	Mixed	Mixed	Mixed
Other	non-European	*default*	*default*	*default*	*default*	*default*

Table S23. Proportion of women falling in different risk categories depending on their lifetime cancer risk (age 20 to 80 years) and their ethnicity, using different risk predictors. Unknown genetic background, mother with BC/EOC at 50y. First panel: lifetime BC risk; the thresholds between risk categories are 17%, between 'population' and 'moderate', and 30%, between 'moderate' and 'high' (NICE guidelines). Second panel: lifetime EOC risk, with thresholds at 3.5% and 5% (threshold for risk-reducing surgery, NICE guidelines).

Predictors	Risk categories	Proportions in each category based on lifetime BC risk					
		UK pop	*White*	*Black*	*East Asian*	*South Asian*	*Mixed*
PGS only	*Population*	0.519	0.483	0.635	0.797	0.795	---
	Moderate	0.446	0.473	0.365	0.203	0.205	---
	High	0.036	0.044	0.000	0.001	0.000	---
QRFs only	*Population*	0.272	0.241	0.571	0.790	0.776	0.911
	Moderate	0.721	0.750	0.429	0.210	0.224	0.089
	High	0.006	0.009	0.001	0.000	0.000	0.000
PGS+QRFs	*Population*	0.532	0.500	0.604	0.757	0.755	---
	Moderate	0.413	0.435	0.394	0.241	0.243	---
	High	0.055	0.065	0.002	0.001	0.002	---
PGS+MD	*Population*	0.535	0.503	0.571	0.741	0.741	---
	Moderate	0.402	0.424	0.427	0.258	0.258	---
	High	0.063	0.072	0.001	0.001	0.001	---
QRFs+MD	*Population*	0.376	0.332	0.560	0.700	0.695	0.813
	Moderate	0.587	0.616	0.429	0.300	0.303	0.186
	High	0.037	0.052	0.010	0.001	0.001	0.001
PGS+QRFs+MD	*Population*	0.542	0.515	0.585	0.724	0.724	---
	Moderate	0.380	0.395	0.400	0.268	0.266	---
	High	0.078	0.089	0.015	0.008	0.009	---

Predictors	Risk categories	Proportions in each category based on lifetime EOC risk					
		UK pop	*White*	*Black*	*East Asian*	*South Asian*	*Mixed*
PGS only	*<3.5%*	0.038	0.026	0.348	0.157	0.199	---
	3.5-5%	0.503	0.444	0.634	0.782	0.721	---
	≥5%	0.459	0.530	0.018	0.060	0.080	---
QRFs only	*<3.5%*	0.148	0.122	0.447	0.310	0.285	0.689
	3.5-5%	0.405	0.395	0.389	0.462	0.516	0.240
	≥5%	0.447	0.482	0.164	0.228	0.200	0.071
PGS+QRFs	*<3.5%*	0.186	0.161	0.486	0.375	0.356	---
	3.5-5%	0.379	0.366	0.345	0.395	0.436	---
	≥5%	0.435	0.472	0.169	0.229	0.208	---

Table S24. Proportion of women falling in different risk categories depending on their 10-year BC risk (age 40 to 50 years) and their ethnicity, using different risk predictors. Unknown genetic background, unknown FH. The thresholds between risk categories are 3%, between 'population' and 'moderate', and 8%, between 'moderate' and 'high' (NICE guidelines).

**Predictors**	**Risk categories**	**Proportions in each category based on 10-year BC risk**					
		UK pop	*White*	*Black*	*East Asian*	*South Asian*	*Mixed*
PGS only	*Population*	0.882	0.870	1.000	0.992	0.991	---
	Moderate	0.117	0.129	0.000	0.008	0.009	---
	High	0.001	0.001	0.000	0.000	0.000	---
QRFs only	*Population*	0.982	0.978	0.997	1.000	1.000	1.000
	Moderate	0.018	0.022	0.003	0.000	0.000	0.000
	High	0.000	0.000	0.000	0.000	0.000	0.000
PGS+QRFs	*Population*	0.873	0.861	0.987	0.982	0.979	---
	Moderate	0.125	0.137	0.013	0.018	0.021	---
	High	0.002	0.003	0.000	0.000	0.000	---
PGS+MD	*Population*	0.869	0.854	0.987	0.982	0.982	---
	Moderate	0.130	0.146	0.013	0.018	0.018	---
	High	0.001	0.001	0.000	0.000	0.000	---
QRFs+MD	*Population*	0.944	0.936	0.984	0.998	0.996	0.997
	Moderate	0.056	0.064	0.016	0.002	0.004	0.003
	High	0.000	0.000	0.000	0.000	0.000	0.000
PGS+QRFs+MD	*Population*	0.860	0.848	0.964	0.971	0.967	---
	Moderate	0.135	0.146	0.036	0.029	0.033	---
	High	0.005	0.006	0.000	0.000	0.000	---

Table S25. Proportion of women falling in different risk categories depending on their 10-year BC risk (age 40 to 50 years) and their ethnicity, using different risk predictors. Unknown genetic background, mother with BC at 50y. The thresholds between risk categories are 3%, between 'population' and 'moderate', and 8%, between 'moderate' and 'high' (NICE guidelines).

Predictors	Risk categories	Proportions in each category based on 10-year BC risk					
		UK pop	*White*	*Black*	*East Asian*	*South Asian*	*Mixed*
PGS only	*Population*	0.576	0.552	0.609	0.756	0.755	---
	Moderate	0.414	0.436	0.391	0.244	0.245	---
	High	0.010	0.012	0.000	0.000	0.000	---
QRFs only	*Population*	0.344	0.285	0.601	0.792	0.722	0.852
	Moderate	0.655	0.714	0.399	0.208	0.278	0.148
	High	0.001	0.001	0.000	0.000	0.000	0.000
PGS+QRFs	*Population*	0.593	0.571	0.610	0.739	0.737	---
	Moderate	0.387	0.406	0.389	0.260	0.262	---
	High	0.020	0.023	0.000	0.000	0.001	---
PGS+MD	*Population*	0.587	0.569	0.550	0.714	0.716	---
	Moderate	0.393	0.406	0.450	0.286	0.284	---
	High	0.019	0.026	0.000	0.000	0.000	---
QRFs+MD	*Population*	0.417	0.388	0.545	0.655	0.673	0.750
	Moderate	0.578	0.606	0.454	0.345	0.326	0.250
	High	0.005	0.006	0.001	0.000	0.000	0.000
PGS+QRFs+MD	*Population*	0.603	0.584	0.589	0.714	0.714	---
	Moderate	0.366	0.381	0.408	0.285	0.284	---
	High	0.031	0.035	0.003	0.002	0.002	---

Formula derivation - PGS for women of mixed genetic ancestry

Preliminary considerations

In BOADICEA, we assume that the PGS explains a proportion of the overall polygenic component α_i^2 in ancestry i, that is the polygenic component is of the form:

$$\tau(t)\left(\alpha_i Z_i + \sqrt{1-\alpha_i^2} Y_i\right)$$

where Y_i is the corresponding unknown polygenic component (also normalised) and $\tau(t)$ is the age-specific standard deviation of the polygenic component.

Assuming populations of similar structure, α_i is also proportional to the effect size per 1 SD, say γ_i. For mixed ancestry samples, we assume we have estimates P_i of the proportion of each ancestry i.

For each individual, given a PGS model, the same raw PGS (R_{pgs}) is computed regardless of their ancestry. R_{pgs} is a weighted sum of SNPs-specific genotypes:

$$R_{pgs} = \sum_{k=1}^{K} w_k g_k$$

where g_k is the genotype for SNP k, w_k is the corresponding weight and K is the total number of SNPs. The weights w_k are typically the log-odds ratio (natural logarithm, i.e. $\ln(OR)$) per allele, but this may not be true in general.

The raw PGS (R_{pgs}) is then normalised separately for each ancestry; so, the normalised PGS Z_i differ by ancestry:

$$Z_i = \frac{\left(R_{pgs} - \mu_i\right)}{\sigma_i}, \qquad \mu_i = E\left(R_{pgs} \middle| pop\ i\right), \qquad \sigma_i^2 = var(R_{pgs}|pop\ i)$$

Calculating *alpha* and *Z-score*

The question is how to combine appropriately the ancestry-specific Z-scores for a woman of mixed ancestry. Since the key aim is to define an appropriate risk level, the natural combined score is of the form:

$$S = \sum_{i=1}^{M} P_i \gamma_i Z_i$$

where P_i is the proportion of an individual's genome derived from population i (which we assume can be estimated from ancestry informative markers) and M is the number of populations (ancestries) considered. The weighting by the effect size γ_i (or, equivalently, by the α_i) makes sense because the ancestry component in which the effect size is larger will make a greater contribution to the risk.

Since the component Z-scores Z_i have mean zero, S will also have mean zero.

Instead, the variance of S is given by:

$$var(S) = \sum_{i=1}^{M}\sum_{j=1}^{M} cov\left(P_i\gamma_i\frac{R_{pgs}-\mu_i}{\sigma_i}, P_j\gamma_j\frac{R_{pgs}-\mu_j}{\sigma_j}\right)$$

$$= \sum_{i=1}^{M}\sum_{j=1}^{M}\frac{P_i\gamma_i}{\sigma_i}\frac{P_j\gamma_j}{\sigma_j}cov\left(R_{pgs}-\mu_i, R_{pgs}-\mu_j\right) = \sum_{i=1}^{M}\sum_{j=1}^{M}\frac{P_i\gamma_i}{\sigma_i}\frac{P_j\gamma_j}{\sigma_j}var\left(R_{pgs}\right)$$

$$= \left(\sum_{i=1}^{M}\frac{P_i\gamma_i}{\sigma_i}\right)^2 var\left(R_{pgs}\right) \equiv \left(\sum_{i=1}^{M}\frac{P_i\gamma_i}{\sigma_i}\right)^2 \sigma^2$$

where $\sigma^2$is the variance of the raw PGS in the admixed population.

Therefore, the final normalised Z-score is:

$$Z = \frac{S}{sd(S)} = \frac{\sum_{i=1}^{M} P_i\gamma_i Z_i}{\sigma\sum_{i=1}^{M}\frac{P_i\gamma_i}{\sigma_i}}$$

The (raw) PGS effect size in population i, defined as the mean difference in the raw PGS between cases and controls is:

$$2\sum_{k=1}^{K} q_{ik}(1-q_{ik})\beta_{ik} = \gamma_i\sigma_i$$

where $\beta_{ik}$is the per-allele effect size for SNP k in population i and $q_{ik}$is the corresponding allele frequency. For the PGS models presented in this manuscript, β_{ik} does not depend on the ancestry; therefore $w_k = \beta_{ik}\forall\, i$, and w_k can be employed in all downstream formulas.

Therefore, the overall normalised effect size per SD for an admixed population is:

$$\gamma = \frac{\sum_{i=1}^{M} P_i\gamma_i\sigma_i}{\sigma}$$

and hence the corresponding α, which is proportional to γ, is:

$$\alpha = \frac{\sum_{i=1}^{M} P_i\alpha_i\sigma_i}{\sigma}$$

Derivation of σ

We note that the variance of the raw PGS (σ) in the admixed population will depend not only on the variance within the populations but also in the variation in genotype frequencies among the populations and the extent to which ancestry at different loci is correlated. To derive σ, we consider three possible models for the population structure:

Model A

An individual in an admixed population is from a given (but unknown) population i, with probability P_i (i.e. the population from which the genotypes are drawn is the same for each locus).

In this case:

$$\sigma^2 = var(R_{pgs}) = E(var(R_{pgs}|pop\ i)) + var\left(E(R_{pgs}|pop\ i)\right) = E(\sigma_i^2) + var(\mu_i)$$

$$= \sum_{i=1}^{M} P_i\sigma_i^2 + \sum_{i=1}^{M} P_i(\mu_i - \mu)^2$$

where $\mu = \sum_{i=1}^{M} P_i\mu_i$ is the overall mean of the raw PGS in the admixed population.

Model B

The genotypes for each SNP are independently drawn from the M populations with probability P_i. Thus, P_i represents the proportion of loci for which the genotypes are drawn from population i, with all SNPs in the PGS being independent (we make the simplifying assumption here that the population distribution for SNPs in the PGS is likely to be similar to that for SNPs defining the ancestry proportions).

In this case:

$$\sigma_i^2 = var(R_{pgs}|pop\ i) = 2\sum_{k=1}^{K} q_{ik}(1 - q_{ik})w_k^2$$

$$\sigma^2 = var(R_{pgs}) = var\left(\sum_{k=1}^{K} w_k g_k\right) = \sum_{k=1}^{K} w_k^2 var(g_k)$$

$$= \sum_{k=1}^{K} w_k^2 \left(E(var(g_k|pop\ i)) + var(E(g_k|pop\ i))\right)$$

$$= \sum_{k=1}^{K} w_k^2 \cdot E(2q_{ik}(1 - q_{ik})) + \sum_{k=1}^{K} w_k^2 \cdot var(2q_{ik})$$

$$= E\left(2\sum_{k=1}^{K} q_{ik}(1 - q_{ik})w_k^2\right) + 4\sum_{k=1}^{K} w_k^2 \cdot var(q_{ik})$$

$$= E(\sigma_i^2) + 4\sum_{k=1}^{K} w_k^2(E(q_{ik}{}^2) - E(q_{ik})^2)$$

$$= \sum_{i=1}^{M} P_i\sigma_i^2 + 4\sum_{k=1}^{K} w_k^2\left(\sum_{i=1}^{M} P_i q_{ik}{}^2 - \left(\sum_{i=1}^{M} P_i q_{ik}\right)^2\right)$$

Model C

As in model B, but allowing for some SNPs to be correlated, i.e. they are in linkage disequilibrium (LD). In this case we write the PGS a sum of contributions from *J* independent blocks of SNPs:

$$R_{pgs} = \sum_{j=1}^{J}\sum_{l} w_{jl} g_{jl}$$

where $g_{jl}$is the genotype for SNP *l* in block j and w_{jl} is the corresponding weight.

We then define the block- and population-specific means, and the block-specific means, as:

$$\mu_{ij} = 2\sum_{l} w_{jl} q_{ijl}\,, \qquad \mu_j = \sum_{i=1}^{M} P_i \mu_{ij}$$

So, the (conditional) mean of R_{prs} in population i is

$$\mu_i = E\left(R_{pgs}\middle|pop\ i\right) = 2\sum_{j=1}^{J}\sum_{l} w_{jl} q_{ijl} = \sum_{j=1}^{J} 2\sum_{l} w_{jl} q_{ijl} = \sum_{j=1}^{J} \mu_{ij}$$

If we make the simplifying assumption that genotypes in the same LD block are drawn from the same population, the formula for model B can be straightforwardly generalised to:

$$\sigma^2 = var\left(R_{pgs}\right) = var\left(\sum_{j=1}^{J}\sum_{l} w_{jl} g_{jl}\right) = \sum_{j=1}^{J}\sum_{l} w_{jl}^2 var\left(g_{jl}\right)$$

$$= \sum_{j=1}^{J}\sum_{l} w_{jl}^2 \left(E\left(var\left(g_{jl}|pop\ i\right)\right) + var\left(E\left(g_{jl}|pop\ i\right)\right)\right)$$

$$= \sum_{j=1}^{J}\sum_{l} w_{jl}^2 \cdot E\left(2q_{ijl}\left(1 - q_{ijl}\right)\right) + \sum_{j=1}^{J}\sum_{l} w_{jl}^2 \cdot var\left(2q_{ijl}\right)$$

$$= E\left(2\sum_{j=1}^{J}\sum_{l} q_{ijl}\left(1 - q_{ijl}\right) w_{jl}^2\right) + \sum_{j=1}^{J} var\left(2\sum_{l} w_{jl} q_{ijl}\right)$$

$$= E\left(\sigma_i^2\right) + \sum_{j=1}^{J} var\left(\mu_{ij}\right)$$

$$= \sum_{i=1}^{M} P_i \sigma_i^2 + \sum_{i=1}^{M} P_i \sum_{j=1}^{J}\left(\mu_{ij} - \mu_j\right)^2$$

That is, the SNP-specific means are replaced by block-specific means.

Final remarks

In practice, most SNPs in the PGS are uncorrelated and there is very little difference between models B and C. Model B is somewhat simpler to use in practice since it only requires the SNP allele frequencies by population. The results in the manuscript assume model B, and the script provided assumes this.

Model C can also be implemented straightforwardly, simply by adding the allele frequencies for SNPs considered in the same block. We also note, however, that model C is still a simplification since genotypes from the same block are assumed to come from a single population.

Model A is the simplest to implement since it only requires the PGS mean by population. However, model B is likely to be the more realistic model. The adjustment to σ may be larger or smaller under model A than model B (or C), depending on the pattern of variation of allele frequencies.
