# Supplementary material for: Adapting the BOADICEA breast and ovarian cancer risk models for the ethnically diverse UK population
Source: Br J Cancer. 2025 Jul 17;133(6):844–55. doi: 10.1038/s41416-025-03117-y (PMC12449465; doi:10.1038/s41416-025-03117-y)
Supplement: Supplementary file 3 — Supplementary Materials - tables S4-S6 [file 41416_2025_3117_MOESM3_ESM.pdf]

Table S4. ER-positive predicted proportions by age at cancer diagnosis and ethnicity, in PV carriers and in the general population. BCAC data from BRIDGES

	ATM						PALB2						CHEK2					
Age at diagnosis	BCAC	White	Black	East Asian	South Asian	Mixed	BCAC	White	Black	East Asian	South Asian	Mixed	BCAC	White	Black	East Asian	South Asian	Mixed
30	0.791	0.832	0.846	0.868	0.782	0.813	0.518	0.581	0.604	0.638	0.495	0.543	0.746	0.815	0.829	0.886	0.782	0.818
31	0.798	0.826	0.833	0.858	0.792	0.789	0.530	0.569	0.581	0.621	0.512	0.508	0.753	0.808	0.813	0.864	0.781	0.779
32	0.805	0.824	0.824	0.840	0.800	0.770	0.541	0.565	0.567	0.593	0.527	0.483	0.761	0.806	0.802	0.826	0.781	0.746
33	0.813	0.826	0.820	0.820	0.809	0.762	0.553	0.570	0.561	0.564	0.542	0.474	0.769	0.808	0.797	0.782	0.783	0.726
34	0.820	0.834	0.822	0.812	0.818	0.768	0.564	0.583	0.564	0.553	0.558	0.483	0.776	0.815	0.799	0.758	0.790	0.727
35	0.826	0.845	0.830	0.824	0.829	0.789	0.576	0.604	0.576	0.575	0.576	0.514	0.783	0.826	0.808	0.770	0.801	0.749
36	0.833	0.858	0.839	0.845	0.839	0.814	0.588	0.627	0.592	0.612	0.594	0.553	0.790	0.839	0.818	0.797	0.813	0.778
37	0.840	0.869	0.847	0.863	0.848	0.834	0.600	0.649	0.606	0.644	0.610	0.586	0.798	0.849	0.828	0.819	0.824	0.801
38	0.846	0.878	0.854	0.877	0.856	0.850	0.611	0.668	0.618	0.672	0.624	0.615	0.805	0.859	0.835	0.837	0.833	0.820
39	0.853	0.886	0.859	0.888	0.862	0.863	0.623	0.685	0.629	0.694	0.636	0.640	0.812	0.867	0.841	0.851	0.840	0.835
40	0.859	0.893	0.864	0.896	0.868	0.873	0.636	0.700	0.638	0.713	0.647	0.660	0.819	0.873	0.846	0.863	0.846	0.847
41	0.866	0.899	0.867	0.903	0.872	0.881	0.648	0.713	0.645	0.727	0.656	0.676	0.826	0.879	0.850	0.872	0.851	0.856
42	0.872	0.904	0.870	0.908	0.876	0.888	0.660	0.724	0.650	0.738	0.663	0.689	0.833	0.884	0.852	0.878	0.854	0.863
43	0.876	0.908	0.872	0.911	0.879	0.892	0.669	0.734	0.654	0.746	0.670	0.700	0.838	0.888	0.854	0.883	0.857	0.868
44	0.880	0.911	0.873	0.913	0.881	0.896	0.678	0.742	0.656	0.751	0.674	0.707	0.843	0.891	0.854	0.886	0.859	0.872
45	0.885	0.914	0.873	0.914	0.883	0.898	0.687	0.748	0.657	0.752	0.678	0.711	0.848	0.894	0.854	0.887	0.860	0.874
46	0.889	0.916	0.873	0.914	0.884	0.899	0.696	0.753	0.657	0.752	0.681	0.714	0.853	0.896	0.853	0.887	0.860	0.875
47	0.893	0.918	0.873	0.914	0.885	0.900	0.705	0.758	0.656	0.751	0.684	0.717	0.858	0.898	0.851	0.887	0.861	0.876
48	0.896	0.920	0.873	0.913	0.887	0.901	0.711	0.763	0.656	0.750	0.687	0.719	0.861	0.900	0.850	0.886	0.862	0.877
49	0.898	0.921	0.872	0.913	0.888	0.902	0.716	0.767	0.655	0.748	0.690	0.722	0.864	0.901	0.849	0.886	0.862	0.878
50	0.901	0.923	0.872	0.912	0.889	0.903	0.722	0.771	0.655	0.746	0.692	0.724	0.868	0.903	0.848	0.885	0.863	0.879
51	0.904	0.924	0.872	0.911	0.891	0.904	0.728	0.774	0.653	0.744	0.695	0.726	0.871	0.904	0.846	0.884	0.864	0.879
52	0.906	0.926	0.871	0.910	0.892	0.905	0.733	0.777	0.652	0.741	0.698	0.727	0.874	0.905	0.845	0.882	0.865	0.880
53	0.908	0.927	0.870	0.909	0.893	0.905	0.737	0.780	0.651	0.738	0.701	0.729	0.876	0.906	0.843	0.880	0.865	0.880
54	0.910	0.928	0.869	0.907	0.894	0.906	0.741	0.782	0.649	0.734	0.704	0.730	0.878	0.907	0.842	0.878	0.866	0.881
55	0.911	0.928	0.869	0.906	0.896	0.907	0.745	0.785	0.648	0.730	0.706	0.731	0.880	0.908	0.840	0.876	0.867	0.881
56	0.913	0.929	0.868	0.904	0.897	0.907	0.749	0.787	0.646	0.726	0.709	0.732	0.882	0.909	0.838	0.874	0.868	0.881
57	0.915	0.930	0.867	0.902	0.898	0.907	0.752	0.788	0.644	0.722	0.711	0.733	0.884	0.909	0.837	0.871	0.869	0.881
58	0.916	0.931	0.866	0.900	0.899	0.907	0.755	0.790	0.642	0.717	0.714	0.733	0.886	0.909	0.835	0.869	0.870	0.881
59	0.917	0.931	0.865	0.898	0.900	0.908	0.758	0.791	0.640	0.713	0.716	0.733	0.887	0.910	0.834	0.866	0.871	0.881
60	0.918	0.931	0.864	0.896	0.901	0.908	0.761	0.792	0.639	0.709	0.718	0.733	0.888	0.910	0.832	0.863	0.872	0.881
61	0.919	0.932	0.864	0.894	0.902	0.907	0.763	0.793	0.637	0.704	0.721	0.733	0.890	0.910	0.831	0.861	0.872	0.880
62	0.920	0.932	0.863	0.893	0.903	0.907	0.766	0.794	0.636	0.700	0.722	0.732	0.891	0.910	0.830	0.858	0.873	0.880
63	0.921	0.932	0.862	0.891	0.904	0.907	0.769	0.794	0.634	0.696	0.724	0.731	0.892	0.910	0.828	0.855	0.874	0.879
64	0.922	0.933	0.862	0.889	0.905	0.907	0.771	0.794	0.633	0.692	0.726	0.730	0.893	0.910	0.827	0.853	0.875	0.879
65	0.923	0.933	0.861	0.887	0.905	0.906	0.773	0.795	0.632	0.688	0.728	0.729	0.894	0.910	0.826	0.850	0.875	0.878
66	0.924	0.933	0.861	0.885	0.906	0.905	0.776	0.794	0.631	0.684	0.729	0.727	0.895	0.910	0.825	0.847	0.876	0.876
67	0.925	0.932	0.860	0.884	0.907	0.905	0.778	0.794	0.629	0.681	0.731	0.725	0.896	0.909	0.824	0.845	0.876	0.875
68	0.926	0.932	0.860	0.882	0.907	0.904	0.781	0.794	0.628	0.677	0.732	0.723	0.897	0.909	0.823	0.842	0.877	0.874
69	0.927	0.932	0.859	0.881	0.908	0.903	0.783	0.793	0.627	0.674	0.733	0.721	0.898	0.908	0.822	0.840	0.877	0.872
70	0.928	0.932	0.859	0.879	0.908	0.902	0.786	0.792	0.626	0.671	0.734	0.718	0.899	0.908	0.821	0.837	0.878	0.870
71	0.930	0.931	0.858	0.878	0.909	0.900	0.789	0.791	0.625	0.667	0.735	0.715	0.900	0.907	0.820	0.834	0.878	0.869
72	0.931	0.931	0.858	0.876	0.909	0.899	0.791	0.790	0.624	0.664	0.735	0.711	0.901	0.906	0.819	0.832	0.878	0.866
73	0.932	0.930	0.857	0.875	0.909	0.897	0.794	0.788	0.623	0.662	0.736	0.707	0.902	0.905	0.818	0.830	0.878	0.864
74	0.933	0.930	0.857	0.873	0.910	0.895	0.796	0.786	0.622	0.659	0.736	0.703	0.903	0.904	0.817	0.827	0.879	0.861
75	0.933	0.929	0.857	0.872	0.910	0.893	0.799	0.784	0.621	0.656	0.737	0.699	0.904	0.903	0.816	0.825	0.879	0.858
76	0.934	0.928	0.856	0.871	0.910	0.891	0.801	0.782	0.620	0.654	0.737	0.694	0.905	0.901	0.815	0.822	0.878	0.855
77	0.935	0.927	0.856	0.870	0.910	0.889	0.803	0.779	0.619	0.651	0.737	0.688	0.906	0.900	0.814	0.820	0.878	0.852
78	0.936	0.926	0.856	0.868	0.910	0.886	0.806	0.777	0.618	0.649	0.736	0.683	0.907	0.898	0.813	0.817	0.878	0.848
79	0.937	0.925	0.855	0.867	0.910	0.884	0.808	0.774	0.617	0.646	0.736	0.676	0.908	0.896	0.812	0.815	0.878	0.843

	RAD51C						RAD51D						BARD1					
Age at diagnosis	BCAC	White	Black	East Asian	South Asian	Mixed	BCAC	White	Black	East Asian	South Asian	Mixed	BCAC	White	Black	East Asian	South Asian	Mixed
30	0.305	0.341	0.364	0.365	0.256	0.291	0.411	0.446	0.471	0.466	0.346	0.386	0.271	0.305	0.327	0.330	0.227	0.259
31	0.315	0.329	0.343	0.359	0.275	0.271	0.422	0.433	0.449	0.462	0.369	0.365	0.281	0.294	0.308	0.324	0.244	0.241
32	0.326	0.325	0.330	0.349	0.292	0.260	0.434	0.429	0.435	0.455	0.391	0.353	0.290	0.291	0.296	0.314	0.260	0.230
33	0.336	0.330	0.325	0.342	0.309	0.258	0.446	0.433	0.429	0.451	0.412	0.353	0.300	0.295	0.291	0.306	0.276	0.228
34	0.347	0.342	0.327	0.345	0.326	0.270	0.458	0.447	0.432	0.458	0.431	0.368	0.310	0.306	0.293	0.308	0.291	0.239
35	0.358	0.362	0.338	0.369	0.342	0.295	0.470	0.469	0.443	0.485	0.449	0.397	0.321	0.326	0.302	0.331	0.307	0.262
36	0.369	0.386	0.351	0.404	0.358	0.327	0.482	0.495	0.457	0.522	0.466	0.434	0.332	0.349	0.315	0.365	0.322	0.292
37	0.381	0.409	0.364	0.436	0.372	0.357	0.495	0.519	0.470	0.554	0.481	0.466	0.343	0.371	0.327	0.396	0.335	0.320
38	0.393	0.431	0.375	0.465	0.385	0.384	0.507	0.542	0.482	0.582	0.495	0.495	0.354	0.392	0.338	0.424	0.348	0.346
39	0.406	0.451	0.384	0.490	0.397	0.408	0.521	0.562	0.492	0.605	0.507	0.520	0.366	0.411	0.347	0.448	0.359	0.369
40	0.419	0.469	0.393	0.510	0.408	0.429	0.534	0.580	0.501	0.625	0.518	0.541	0.379	0.429	0.355	0.469	0.369	0.390
41	0.432	0.486	0.400	0.527	0.417	0.447	0.548	0.596	0.508	0.640	0.527	0.559	0.392	0.445	0.362	0.485	0.378	0.407
42	0.446	0.500	0.405	0.539	0.425	0.462	0.562	0.610	0.514	0.651	0.536	0.573	0.405	0.460	0.367	0.498	0.386	0.421
43	0.455	0.513	0.409	0.548	0.432	0.473	0.571	0.622	0.518	0.659	0.543	0.585	0.415	0.472	0.371	0.507	0.393	0.433
44	0.465	0.523	0.412	0.553	0.438	0.481	0.581	0.632	0.520	0.662	0.549	0.593	0.424	0.482	0.373	0.511	0.399	0.441
45	0.475	0.532	0.413	0.553	0.442	0.487	0.591	0.640	0.522	0.663	0.553	0.598	0.434	0.491	0.374	0.512	0.403	0.446
46	0.486	0.539	0.413	0.552	0.446	0.490	0.601	0.647	0.522	0.661	0.557	0.601	0.445	0.498	0.375	0.511	0.407	0.450
47	0.496	0.546	0.413	0.550	0.450	0.493	0.611	0.653	0.522	0.659	0.561	0.604	0.455	0.505	0.375	0.509	0.410	0.453
48	0.503	0.552	0.413	0.547	0.454	0.496	0.617	0.659	0.522	0.656	0.565	0.607	0.461	0.512	0.374	0.506	0.414	0.456
49	0.509	0.558	0.412	0.544	0.458	0.499	0.622	0.664	0.521	0.653	0.569	0.609	0.468	0.517	0.374	0.503	0.418	0.458
50	0.515	0.563	0.411	0.540	0.461	0.501	0.628	0.669	0.520	0.650	0.572	0.611	0.474	0.523	0.373	0.499	0.421	0.461
51	0.522	0.568	0.410	0.536	0.465	0.503	0.634	0.673	0.519	0.646	0.576	0.613	0.481	0.528	0.372	0.495	0.425	0.463
52	0.529	0.572	0.409	0.532	0.468	0.505	0.640	0.677	0.518	0.642	0.579	0.615	0.487	0.532	0.371	0.491	0.428	0.464
53	0.533	0.576	0.407	0.527	0.472	0.507	0.644	0.680	0.517	0.637	0.583	0.616	0.491	0.536	0.369	0.486	0.432	0.466
54	0.537	0.579	0.406	0.521	0.475	0.508	0.648	0.683	0.515	0.632	0.586	0.617	0.496	0.539	0.368	0.480	0.435	0.467
55	0.541	0.582	0.404	0.515	0.478	0.509	0.651	0.686	0.513	0.626	0.589	0.618	0.500	0.542	0.366	0.474	0.438	0.468
56	0.545	0.585	0.402	0.509	0.482	0.509	0.655	0.688	0.510	0.620	0.592	0.619	0.504	0.545	0.364	0.468	0.441	0.469
57	0.550	0.587	0.399	0.503	0.485	0.510	0.659	0.690	0.508	0.614	0.595	0.619	0.509	0.547	0.362	0.462	0.444	0.469
58	0.553	0.589	0.397	0.497	0.487	0.510	0.662	0.692	0.506	0.608	0.598	0.619	0.512	0.549	0.360	0.456	0.447	0.469
59	0.556	0.591	0.395	0.491	0.490	0.509	0.664	0.693	0.504	0.602	0.600	0.618	0.515	0.551	0.358	0.450	0.450	0.469
60	0.559	0.592	0.393	0.485	0.492	0.508	0.667	0.694	0.501	0.596	0.603	0.617	0.518	0.552	0.356	0.444	0.452	0.468
61	0.562	0.593	0.391	0.479	0.495	0.507	0.670	0.695	0.499	0.591	0.605	0.616	0.522	0.553	0.354	0.439	0.454	0.467
62	0.566	0.594	0.389	0.474	0.497	0.506	0.673	0.695	0.497	0.585	0.606	0.615	0.525	0.554	0.352	0.433	0.456	0.466
63	0.569	0.594	0.387	0.468	0.498	0.504	0.675	0.695	0.495	0.580	0.608	0.613	0.528	0.554	0.350	0.428	0.458	0.464
64	0.572	0.594	0.385	0.463	0.500	0.502	0.678	0.695	0.492	0.575	0.609	0.611	0.531	0.554	0.348	0.423	0.460	0.462
65	0.575	0.594	0.383	0.458	0.501	0.500	0.681	0.695	0.490	0.570	0.611	0.608	0.534	0.554	0.346	0.418	0.461	0.459
66	0.578	0.593	0.381	0.453	0.503	0.497	0.684	0.694	0.488	0.565	0.612	0.606	0.538	0.553	0.344	0.414	0.462	0.457
67	0.581	0.592	0.379	0.449	0.504	0.494	0.686	0.693	0.486	0.560	0.613	0.603	0.541	0.552	0.343	0.409	0.463	0.454
68	0.585	0.591	0.378	0.444	0.504	0.490	0.689	0.692	0.484	0.556	0.613	0.599	0.544	0.551	0.341	0.405	0.464	0.450
69	0.588	0.589	0.376	0.440	0.505	0.486	0.692	0.691	0.482	0.552	0.614	0.595	0.548	0.549	0.339	0.401	0.465	0.446
70	0.592	0.587	0.374	0.436	0.505	0.482	0.695	0.689	0.480	0.548	0.614	0.591	0.551	0.547	0.337	0.397	0.465	0.442
71	0.595	0.585	0.372	0.433	0.506	0.478	0.698	0.687	0.478	0.544	0.614	0.586	0.555	0.545	0.336	0.393	0.465	0.438
72	0.599	0.582	0.370	0.429	0.506	0.473	0.701	0.684	0.476	0.540	0.614	0.581	0.559	0.542	0.334	0.390	0.465	0.433
73	0.602	0.579	0.369	0.426	0.505	0.467	0.704	0.681	0.474	0.537	0.613	0.576	0.562	0.539	0.332	0.387	0.465	0.428
74	0.605	0.576	0.367	0.423	0.505	0.461	0.707	0.678	0.471	0.534	0.612	0.570	0.566	0.536	0.331	0.384	0.465	0.422
75	0.609	0.572	0.365	0.420	0.504	0.455	0.710	0.675	0.469	0.530	0.612	0.564	0.569	0.532	0.329	0.381	0.464	0.416
76	0.612	0.568	0.363	0.417	0.503	0.449	0.713	0.671	0.467	0.528	0.610	0.557	0.573	0.528	0.327	0.378	0.463	0.410
77	0.616	0.564	0.362	0.414	0.502	0.442	0.716	0.667	0.465	0.525	0.609	0.550	0.576	0.524	0.326	0.376	0.462	0.403
78	0.619	0.559	0.360	0.412	0.500	0.435	0.719	0.663	0.463	0.522	0.607	0.543	0.580	0.519	0.324	0.373	0.461	0.396
79	0.623	0.554	0.358	0.409	0.499	0.427	0.722	0.658	0.461	0.520	0.606	0.535	0.584	0.514	0.323	0.371	0.459	0.388

	BRCA1					BRCA2					Population						
Age at diagnosis	White	Black	East Asian	South Asian	Mixed	White	Black	East Asian	South Asian	Mixed	BCAC	White	Black	East Asian	South Asian	Mixed	NA
30	0.275	0.296	0.293	0.200	0.229	0.885	0.894	0.902	0.841	0.864	0.626	0.690	0.710	0.750	0.618	0.663	1.000
31	0.259	0.272	0.282	0.212	0.209	0.876	0.882	0.895	0.848	0.846	0.636	0.680	0.690	0.732	0.631	0.627	1.000
32	0.247	0.252	0.268	0.220	0.193	0.870	0.872	0.882	0.852	0.829	0.647	0.677	0.676	0.702	0.642	0.600	1.000
33	0.241	0.238	0.254	0.225	0.185	0.866	0.863	0.868	0.854	0.818	0.657	0.681	0.671	0.669	0.653	0.588	1.000
34	0.240	0.229	0.249	0.228	0.185	0.866	0.857	0.859	0.855	0.816	0.668	0.692	0.674	0.655	0.667	0.595	1.000
35	0.245	0.226	0.259	0.231	0.196	0.868	0.855	0.863	0.857	0.825	0.678	0.710	0.685	0.673	0.683	0.624	1.000
36	0.256	0.228	0.278	0.234	0.212	0.872	0.854	0.873	0.857	0.837	0.688	0.730	0.700	0.706	0.699	0.660	1.000
37	0.267	0.230	0.297	0.238	0.228	0.873	0.851	0.878	0.854	0.844	0.699	0.747	0.712	0.735	0.714	0.691	1.000
38	0.278	0.233	0.314	0.242	0.243	0.872	0.845	0.881	0.849	0.847	0.709	0.763	0.723	0.758	0.726	0.716	1.000
39	0.290	0.236	0.331	0.247	0.257	0.870	0.837	0.882	0.843	0.847	0.719	0.776	0.732	0.777	0.737	0.737	1.000
40	0.302	0.239	0.345	0.252	0.271	0.868	0.829	0.881	0.837	0.847	0.730	0.788	0.740	0.792	0.746	0.754	1.000
41	0.315	0.243	0.359	0.258	0.284	0.866	0.821	0.880	0.831	0.846	0.740	0.798	0.746	0.804	0.753	0.768	1.000
42	0.326	0.246	0.369	0.263	0.294	0.863	0.813	0.877	0.824	0.843	0.750	0.807	0.750	0.813	0.759	0.779	1.000
43	0.335	0.247	0.374	0.266	0.302	0.860	0.803	0.873	0.817	0.839	0.758	0.814	0.753	0.820	0.764	0.787	1.000
44	0.341	0.245	0.374	0.267	0.305	0.856	0.793	0.867	0.809	0.834	0.765	0.820	0.755	0.823	0.768	0.793	1.000
45	0.344	0.242	0.369	0.267	0.305	0.852	0.783	0.860	0.802	0.828	0.772	0.825	0.756	0.825	0.771	0.796	1.000
46	0.346	0.239	0.363	0.266	0.303	0.849	0.774	0.853	0.796	0.822	0.780	0.829	0.755	0.825	0.773	0.799	1.000
47	0.348	0.236	0.356	0.266	0.303	0.845	0.764	0.845	0.789	0.816	0.787	0.833	0.755	0.824	0.775	0.801	1.000
48	0.352	0.234	0.351	0.267	0.303	0.840	0.752	0.835	0.781	0.808	0.792	0.836	0.755	0.824	0.778	0.803	1.000
49	0.356	0.232	0.347	0.269	0.304	0.834	0.739	0.824	0.771	0.799	0.796	0.839	0.754	0.823	0.780	0.805	1.000
50	0.361	0.232	0.343	0.272	0.306	0.828	0.726	0.813	0.763	0.790	0.801	0.842	0.753	0.821	0.782	0.806	1.000
51	0.367	0.232	0.340	0.276	0.309	0.823	0.714	0.802	0.755	0.782	0.806	0.845	0.752	0.820	0.784	0.808	1.000
52	0.373	0.233	0.338	0.281	0.312	0.818	0.701	0.790	0.747	0.774	0.810	0.847	0.751	0.818	0.786	0.809	1.000
53	0.378	0.233	0.335	0.285	0.315	0.813	0.689	0.779	0.741	0.766	0.814	0.849	0.750	0.816	0.788	0.810	1.000
54	0.382	0.232	0.331	0.289	0.316	0.808	0.679	0.767	0.735	0.759	0.817	0.851	0.749	0.813	0.791	0.811	1.000
55	0.386	0.231	0.326	0.292	0.318	0.805	0.669	0.757	0.731	0.754	0.820	0.853	0.748	0.810	0.793	0.812	1.000
56	0.391	0.232	0.322	0.297	0.320	0.802	0.661	0.747	0.728	0.749	0.823	0.854	0.746	0.807	0.795	0.813	1.000
57	0.399	0.234	0.322	0.305	0.326	0.800	0.654	0.738	0.725	0.745	0.826	0.855	0.745	0.804	0.797	0.814	1.000
58	0.409	0.239	0.324	0.315	0.334	0.796	0.645	0.728	0.721	0.739	0.828	0.857	0.743	0.800	0.799	0.814	1.000
59	0.421	0.246	0.329	0.326	0.343	0.792	0.635	0.716	0.717	0.733	0.830	0.858	0.742	0.797	0.801	0.814	1.000
60	0.434	0.253	0.334	0.339	0.353	0.787	0.626	0.705	0.712	0.726	0.833	0.859	0.741	0.794	0.803	0.814	1.000
61	0.446	0.259	0.338	0.351	0.362	0.782	0.615	0.693	0.707	0.718	0.835	0.859	0.740	0.791	0.804	0.814	1.000
62	0.456	0.265	0.342	0.361	0.369	0.776	0.604	0.680	0.701	0.709	0.837	0.860	0.739	0.787	0.806	0.814	1.000
63	0.464	0.270	0.344	0.370	0.375	0.769	0.593	0.667	0.694	0.699	0.839	0.860	0.738	0.784	0.807	0.813	1.000
64	0.469	0.272	0.344	0.377	0.378	0.762	0.582	0.653	0.687	0.690	0.841	0.861	0.737	0.781	0.809	0.813	1.000
65	0.471	0.272	0.341	0.380	0.377	0.756	0.572	0.642	0.682	0.681	0.843	0.861	0.736	0.778	0.810	0.812	1.000
66	0.469	0.270	0.336	0.380	0.374	0.752	0.565	0.632	0.678	0.674	0.844	0.861	0.735	0.775	0.812	0.811	1.000
67	0.465	0.266	0.329	0.378	0.368	0.748	0.559	0.624	0.676	0.668	0.846	0.861	0.734	0.772	0.813	0.810	1.000
68	0.461	0.262	0.323	0.376	0.362	0.745	0.555	0.618	0.674	0.662	0.848	0.860	0.734	0.770	0.814	0.808	1.000
69	0.457	0.259	0.318	0.375	0.356	0.743	0.552	0.612	0.673	0.658	0.850	0.860	0.733	0.767	0.815	0.806	1.000
70	0.454	0.256	0.313	0.374	0.351	0.741	0.549	0.607	0.673	0.653	0.852	0.859	0.732	0.764	0.816	0.804	1.000
71	0.450	0.254	0.309	0.373	0.346	0.738	0.546	0.603	0.672	0.648	0.854	0.859	0.732	0.762	0.817	0.802	1.000
72	0.446	0.251	0.305	0.371	0.340	0.735	0.543	0.598	0.672	0.643	0.856	0.858	0.731	0.759	0.818	0.800	1.000
73	0.441	0.248	0.300	0.368	0.333	0.731	0.539	0.593	0.670	0.636	0.858	0.857	0.731	0.757	0.818	0.797	1.000
74	0.434	0.244	0.295	0.365	0.325	0.726	0.535	0.587	0.667	0.628	0.860	0.856	0.730	0.755	0.819	0.794	1.000
75	0.426	0.240	0.289	0.360	0.316	0.720	0.529	0.580	0.663	0.619	0.862	0.854	0.730	0.753	0.819	0.790	1.000
76	0.417	0.234	0.283	0.354	0.306	0.713	0.523	0.573	0.658	0.608	0.864	0.853	0.729	0.751	0.820	0.787	1.000
77	0.407	0.228	0.275	0.348	0.295	0.705	0.515	0.564	0.652	0.595	0.866	0.851	0.729	0.749	0.820	0.783	1.000
78	0.396	0.222	0.268	0.340	0.283	0.696	0.507	0.555	0.645	0.582	0.867	0.849	0.728	0.747	0.820	0.778	1.000
79	0.385	0.217	0.262	0.333	0.272	0.687	0.500	0.547	0.638	0.569	0.869	0.847	0.728	0.745	0.820	0.774	1.000

Table S5. ER-negative non-TN predicted proportions by age at cancer diagnosis and ethnicity, in PV carriers and in the general population. BCAC data from BRIDGES

	ATM						PALB2						CHEK2					
Age at diagnosis	BCAC	White	Black	East Asian	South Asian	Mixed	BCAC	White	Black	East Asian	South Asian	Mixed	BCAC	White	Black	East Asian	South Asian	Mixed
30	0.088	0.049	0.046	0.008	0.041	0.031	0.183	0.108	0.104	0.020	0.083	0.066	0.167	0.096	0.090	0.017	0.083	0.063
31	0.085	0.050	0.051	0.021	0.050	0.049	0.179	0.109	0.113	0.048	0.102	0.101	0.162	0.098	0.100	0.042	0.098	0.098
32	0.083	0.050	0.054	0.043	0.056	0.068	0.176	0.108	0.118	0.097	0.118	0.136	0.157	0.098	0.106	0.085	0.111	0.133
33	0.080	0.049	0.056	0.071	0.060	0.082	0.172	0.107	0.120	0.155	0.128	0.162	0.152	0.096	0.109	0.137	0.118	0.158
34	0.077	0.047	0.054	0.090	0.061	0.087	0.168	0.104	0.118	0.194	0.131	0.172	0.147	0.093	0.106	0.169	0.118	0.165
35	0.074	0.045	0.051	0.088	0.057	0.079	0.164	0.101	0.112	0.194	0.125	0.164	0.142	0.088	0.099	0.165	0.111	0.152
36	0.072	0.042	0.047	0.076	0.052	0.069	0.160	0.097	0.104	0.173	0.117	0.148	0.137	0.082	0.092	0.143	0.102	0.132
37	0.069	0.039	0.043	0.066	0.048	0.060	0.156	0.093	0.098	0.156	0.110	0.134	0.132	0.077	0.085	0.126	0.094	0.116
38	0.066	0.037	0.040	0.058	0.045	0.054	0.152	0.090	0.093	0.142	0.104	0.123	0.127	0.073	0.080	0.112	0.088	0.104
39	0.064	0.035	0.038	0.052	0.042	0.048	0.148	0.087	0.089	0.129	0.099	0.113	0.122	0.070	0.075	0.101	0.083	0.094
40	0.061	0.034	0.037	0.047	0.040	0.044	0.144	0.084	0.085	0.119	0.095	0.106	0.118	0.066	0.072	0.092	0.079	0.086
41	0.059	0.032	0.035	0.044	0.039	0.041	0.139	0.081	0.083	0.111	0.092	0.100	0.113	0.064	0.069	0.085	0.076	0.080
42	0.056	0.031	0.034	0.041	0.037	0.039	0.135	0.079	0.081	0.105	0.090	0.095	0.108	0.061	0.068	0.079	0.073	0.075
43	0.054	0.030	0.034	0.039	0.037	0.037	0.131	0.077	0.080	0.100	0.088	0.091	0.105	0.059	0.066	0.075	0.072	0.072
44	0.052	0.029	0.034	0.037	0.036	0.035	0.128	0.075	0.080	0.097	0.087	0.089	0.101	0.057	0.066	0.072	0.071	0.069
45	0.050	0.028	0.034	0.036	0.036	0.035	0.124	0.074	0.080	0.094	0.087	0.087	0.097	0.056	0.066	0.071	0.070	0.068
46	0.048	0.028	0.034	0.036	0.036	0.034	0.120	0.072	0.081	0.093	0.088	0.086	0.094	0.055	0.067	0.070	0.070	0.067
47	0.047	0.027	0.034	0.035	0.036	0.034	0.116	0.071	0.081	0.092	0.088	0.085	0.090	0.053	0.067	0.069	0.070	0.066
48	0.045	0.027	0.034	0.035	0.036	0.033	0.113	0.070	0.082	0.091	0.088	0.084	0.087	0.052	0.068	0.068	0.070	0.065
49	0.044	0.026	0.035	0.035	0.036	0.033	0.110	0.069	0.083	0.090	0.088	0.083	0.084	0.051	0.068	0.068	0.070	0.064
50	0.042	0.026	0.035	0.034	0.035	0.032	0.107	0.068	0.083	0.089	0.087	0.082	0.082	0.050	0.068	0.067	0.069	0.063
51	0.041	0.025	0.035	0.034	0.035	0.032	0.104	0.067	0.083	0.089	0.087	0.081	0.079	0.049	0.069	0.067	0.069	0.062
52	0.039	0.025	0.035	0.034	0.035	0.031	0.101	0.066	0.084	0.088	0.087	0.080	0.076	0.049	0.069	0.067	0.068	0.061
53	0.038	0.024	0.035	0.034	0.035	0.031	0.098	0.065	0.084	0.088	0.086	0.079	0.074	0.048	0.069	0.067	0.068	0.060
54	0.037	0.024	0.036	0.034	0.034	0.031	0.096	0.064	0.084	0.088	0.085	0.078	0.072	0.047	0.069	0.067	0.067	0.060
55	0.036	0.024	0.036	0.035	0.034	0.030	0.094	0.063	0.084	0.088	0.085	0.077	0.070	0.046	0.070	0.067	0.066	0.059
56	0.035	0.023	0.036	0.035	0.034	0.030	0.091	0.062	0.084	0.089	0.084	0.076	0.068	0.046	0.070	0.068	0.065	0.058
57	0.034	0.023	0.036	0.035	0.033	0.029	0.089	0.062	0.084	0.089	0.083	0.075	0.066	0.045	0.070	0.068	0.064	0.057
58	0.033	0.023	0.036	0.035	0.033	0.029	0.087	0.061	0.084	0.089	0.082	0.074	0.065	0.044	0.069	0.069	0.063	0.057
59	0.033	0.022	0.036	0.036	0.032	0.029	0.086	0.060	0.084	0.090	0.081	0.073	0.064	0.044	0.069	0.069	0.063	0.056
60	0.032	0.022	0.036	0.036	0.032	0.028	0.084	0.059	0.083	0.090	0.080	0.072	0.062	0.043	0.069	0.070	0.062	0.055
61	0.031	0.022	0.035	0.036	0.031	0.028	0.082	0.059	0.083	0.090	0.079	0.072	0.061	0.043	0.068	0.070	0.061	0.055
62	0.031	0.022	0.035	0.036	0.031	0.028	0.081	0.058	0.082	0.091	0.077	0.071	0.060	0.042	0.068	0.071	0.059	0.054
63	0.030	0.021	0.035	0.037	0.030	0.027	0.079	0.057	0.081	0.091	0.076	0.070	0.059	0.042	0.067	0.071	0.058	0.053
64	0.030	0.021	0.034	0.037	0.029	0.027	0.078	0.057	0.080	0.091	0.075	0.069	0.058	0.041	0.066	0.072	0.057	0.053
65	0.029	0.021	0.034	0.037	0.029	0.027	0.077	0.056	0.079	0.092	0.073	0.068	0.057	0.041	0.066	0.072	0.056	0.052
66	0.029	0.021	0.034	0.038	0.028	0.027	0.076	0.056	0.078	0.092	0.072	0.068	0.056	0.040	0.065	0.073	0.055	0.052
67	0.028	0.020	0.033	0.038	0.028	0.026	0.075	0.055	0.076	0.093	0.071	0.067	0.055	0.040	0.064	0.073	0.054	0.051
68	0.028	0.020	0.032	0.038	0.027	0.026	0.074	0.054	0.075	0.093	0.069	0.066	0.054	0.040	0.062	0.073	0.053	0.051
69	0.027	0.020	0.032	0.039	0.026	0.026	0.073	0.054	0.073	0.093	0.067	0.066	0.053	0.039	0.061	0.074	0.051	0.050
70	0.027	0.020	0.031	0.039	0.026	0.026	0.072	0.053	0.072	0.094	0.066	0.065	0.052	0.039	0.060	0.074	0.050	0.050
71	0.026	0.020	0.030	0.039	0.025	0.026	0.071	0.053	0.070	0.094	0.064	0.064	0.051	0.039	0.059	0.075	0.049	0.050
72	0.026	0.020	0.030	0.039	0.024	0.025	0.069	0.052	0.068	0.095	0.063	0.064	0.050	0.038	0.057	0.075	0.048	0.049
73	0.025	0.019	0.029	0.040	0.024	0.025	0.068	0.052	0.067	0.095	0.061	0.063	0.049	0.038	0.056	0.076	0.046	0.049
74	0.025	0.019	0.028	0.040	0.023	0.025	0.067	0.051	0.065	0.095	0.059	0.063	0.048	0.038	0.054	0.076	0.045	0.049
75	0.024	0.019	0.027	0.040	0.022	0.025	0.066	0.051	0.063	0.096	0.058	0.062	0.048	0.037	0.052	0.077	0.044	0.049
76	0.024	0.019	0.027	0.041	0.022	0.025	0.065	0.051	0.061	0.097	0.056	0.062	0.047	0.037	0.051	0.077	0.042	0.049
77	0.024	0.019	0.026	0.041	0.021	0.025	0.064	0.050	0.059	0.097	0.054	0.062	0.046	0.037	0.049	0.078	0.041	0.049
78	0.023	0.019	0.025	0.041	0.021	0.025	0.063	0.050	0.057	0.098	0.053	0.062	0.045	0.037	0.047	0.078	0.040	0.049
79	0.023	0.019	0.024	0.042	0.020	0.025	0.062	0.049	0.055	0.098	0.051	0.061	0.044	0.036	0.046	0.079	0.039	0.049

	RAD51C						RAD51D						BARD1					
Age at diagnosis	BCAC	White	Black	East Asian	South Asian	Mixed	BCAC	White	Black	East Asian	South Asian	Mixed	BCAC	White	Black	East Asian	South Asian	Mixed
30	0.139	0.081	0.080	0.015	0.055	0.046	0.094	0.053	0.052	0.009	0.037	0.030	0.152	0.090	0.089	0.016	0.060	0.050
31	0.137	0.081	0.086	0.035	0.070	0.069	0.092	0.053	0.056	0.023	0.047	0.047	0.151	0.089	0.094	0.039	0.077	0.076
32	0.136	0.080	0.089	0.073	0.084	0.094	0.091	0.053	0.059	0.048	0.056	0.064	0.149	0.088	0.098	0.081	0.092	0.102
33	0.134	0.079	0.090	0.121	0.094	0.114	0.089	0.052	0.059	0.080	0.063	0.078	0.148	0.088	0.099	0.133	0.103	0.124
34	0.133	0.079	0.088	0.155	0.098	0.124	0.088	0.051	0.058	0.103	0.065	0.084	0.146	0.087	0.097	0.171	0.108	0.135
35	0.131	0.078	0.084	0.160	0.096	0.121	0.086	0.050	0.055	0.105	0.063	0.082	0.145	0.086	0.093	0.176	0.106	0.132
36	0.129	0.077	0.079	0.147	0.091	0.112	0.085	0.049	0.052	0.095	0.059	0.075	0.143	0.085	0.088	0.164	0.100	0.124
37	0.128	0.076	0.076	0.136	0.086	0.105	0.083	0.048	0.049	0.087	0.056	0.069	0.141	0.084	0.084	0.152	0.095	0.116
38	0.126	0.074	0.072	0.126	0.082	0.098	0.081	0.047	0.047	0.079	0.053	0.064	0.139	0.083	0.080	0.141	0.091	0.109
39	0.124	0.073	0.070	0.117	0.079	0.093	0.080	0.046	0.045	0.073	0.051	0.059	0.138	0.082	0.077	0.132	0.088	0.104
40	0.122	0.072	0.068	0.110	0.077	0.088	0.078	0.045	0.043	0.067	0.049	0.056	0.136	0.081	0.075	0.124	0.086	0.099
41	0.119	0.071	0.066	0.104	0.075	0.085	0.076	0.044	0.042	0.063	0.048	0.053	0.133	0.080	0.074	0.118	0.084	0.095
42	0.117	0.070	0.065	0.099	0.074	0.082	0.074	0.043	0.041	0.060	0.047	0.051	0.131	0.079	0.073	0.112	0.083	0.092
43	0.115	0.069	0.064	0.094	0.073	0.079	0.072	0.042	0.041	0.057	0.046	0.049	0.129	0.078	0.072	0.108	0.082	0.089
44	0.113	0.068	0.064	0.091	0.073	0.078	0.070	0.041	0.041	0.055	0.046	0.048	0.127	0.077	0.072	0.104	0.082	0.087
45	0.110	0.067	0.065	0.089	0.073	0.076	0.069	0.041	0.041	0.054	0.046	0.047	0.124	0.077	0.072	0.102	0.082	0.086
46	0.108	0.067	0.065	0.088	0.074	0.076	0.067	0.040	0.041	0.053	0.046	0.047	0.121	0.076	0.073	0.100	0.083	0.086
47	0.105	0.066	0.066	0.086	0.074	0.075	0.065	0.039	0.042	0.052	0.046	0.046	0.119	0.075	0.074	0.098	0.083	0.085
48	0.103	0.065	0.066	0.085	0.075	0.074	0.063	0.039	0.042	0.051	0.046	0.045	0.116	0.074	0.074	0.097	0.084	0.084
49	0.101	0.064	0.067	0.084	0.075	0.073	0.062	0.038	0.042	0.051	0.047	0.045	0.114	0.073	0.075	0.096	0.084	0.083
50	0.098	0.064	0.067	0.083	0.075	0.073	0.060	0.038	0.043	0.050	0.047	0.044	0.111	0.073	0.075	0.094	0.084	0.082
51	0.096	0.063	0.067	0.082	0.075	0.072	0.058	0.037	0.043	0.050	0.046	0.044	0.109	0.072	0.075	0.093	0.084	0.081
52	0.093	0.062	0.068	0.081	0.075	0.071	0.057	0.037	0.043	0.049	0.046	0.043	0.106	0.071	0.075	0.093	0.084	0.081
53	0.091	0.062	0.068	0.081	0.075	0.070	0.055	0.036	0.043	0.049	0.046	0.043	0.104	0.070	0.075	0.092	0.084	0.080
54	0.089	0.061	0.068	0.080	0.074	0.070	0.054	0.036	0.043	0.049	0.046	0.042	0.102	0.070	0.076	0.091	0.084	0.079
55	0.087	0.060	0.068	0.080	0.074	0.069	0.053	0.036	0.043	0.049	0.046	0.042	0.100	0.069	0.075	0.091	0.083	0.078
56	0.085	0.060	0.067	0.080	0.073	0.068	0.051	0.035	0.043	0.049	0.045	0.041	0.097	0.068	0.075	0.090	0.083	0.077
57	0.083	0.059	0.067	0.080	0.073	0.067	0.050	0.035	0.043	0.049	0.045	0.041	0.095	0.068	0.075	0.090	0.082	0.076
58	0.082	0.058	0.067	0.079	0.072	0.066	0.049	0.034	0.043	0.049	0.044	0.040	0.094	0.067	0.075	0.090	0.081	0.075
59	0.081	0.058	0.066	0.079	0.071	0.065	0.048	0.034	0.042	0.049	0.044	0.040	0.092	0.066	0.074	0.090	0.080	0.074
60	0.079	0.057	0.066	0.079	0.070	0.065	0.047	0.034	0.042	0.049	0.043	0.039	0.091	0.066	0.073	0.089	0.080	0.073
61	0.078	0.056	0.065	0.079	0.069	0.064	0.046	0.033	0.042	0.049	0.043	0.039	0.089	0.065	0.073	0.089	0.079	0.072
62	0.076	0.056	0.064	0.079	0.068	0.063	0.046	0.033	0.041	0.049	0.042	0.038	0.087	0.064	0.072	0.089	0.077	0.071
63	0.076	0.055	0.064	0.079	0.067	0.062	0.045	0.032	0.041	0.049	0.041	0.038	0.086	0.063	0.071	0.089	0.076	0.070
64	0.075	0.055	0.063	0.079	0.066	0.061	0.044	0.032	0.040	0.049	0.040	0.037	0.086	0.063	0.070	0.088	0.075	0.069
65	0.074	0.054	0.062	0.079	0.065	0.060	0.044	0.032	0.039	0.049	0.040	0.037	0.085	0.062	0.069	0.088	0.074	0.068
66	0.073	0.053	0.060	0.079	0.064	0.059	0.043	0.031	0.039	0.049	0.039	0.036	0.084	0.061	0.067	0.088	0.072	0.067
67	0.072	0.053	0.059	0.078	0.063	0.059	0.043	0.031	0.038	0.049	0.038	0.036	0.083	0.061	0.066	0.088	0.071	0.066
68	0.071	0.052	0.058	0.078	0.061	0.058	0.042	0.031	0.037	0.049	0.037	0.035	0.082	0.060	0.064	0.088	0.069	0.065
69	0.070	0.052	0.057	0.078	0.060	0.057	0.041	0.030	0.036	0.049	0.036	0.035	0.081	0.059	0.063	0.088	0.068	0.064
70	0.069	0.051	0.055	0.078	0.058	0.056	0.041	0.030	0.035	0.049	0.036	0.034	0.080	0.058	0.061	0.088	0.066	0.063
71	0.068	0.050	0.054	0.078	0.057	0.055	0.040	0.030	0.035	0.049	0.035	0.034	0.079	0.058	0.060	0.088	0.064	0.062
72	0.067	0.050	0.052	0.079	0.055	0.055	0.040	0.029	0.034	0.050	0.034	0.034	0.078	0.057	0.058	0.088	0.063	0.062
73	0.067	0.049	0.051	0.079	0.054	0.054	0.039	0.029	0.033	0.050	0.033	0.033	0.077	0.056	0.056	0.088	0.061	0.061
74	0.066	0.048	0.049	0.079	0.052	0.053	0.038	0.029	0.032	0.050	0.032	0.033	0.076	0.056	0.055	0.088	0.059	0.060
75	0.065	0.048	0.047	0.079	0.051	0.052	0.038	0.028	0.031	0.050	0.031	0.032	0.075	0.055	0.053	0.088	0.058	0.059
76	0.064	0.047	0.046	0.079	0.049	0.052	0.037	0.028	0.030	0.050	0.030	0.032	0.074	0.054	0.051	0.088	0.056	0.058
77	0.063	0.047	0.044	0.079	0.048	0.051	0.037	0.028	0.028	0.050	0.029	0.032	0.073	0.053	0.049	0.089	0.054	0.057
78	0.062	0.046	0.042	0.080	0.046	0.050	0.036	0.027	0.027	0.051	0.028	0.032	0.072	0.053	0.047	0.089	0.052	0.057
79	0.061	0.045	0.041	0.080	0.044	0.050	0.036	0.027	0.026	0.051	0.027	0.031	0.071	0.052	0.045	0.089	0.050	0.056

	BRCA1					BRCA2					Population						
Age at diagnosis	White	Black	East Asian	South Asian	Mixed	White	Black	East Asian	South Asian	Mixed	BCAC	White	Black	East Asian	South Asian	Mixed	NA
30	0.071	0.071	0.013	0.046	0.039	0.023	0.022	0.004	0.020	0.015	0.166	0.096	0.091	0.017	0.077	0.061	1.000
31	0.070	0.074	0.030	0.059	0.058	0.024	0.025	0.010	0.024	0.024	0.162	0.097	0.100	0.042	0.094	0.093	1.000
32	0.068	0.076	0.063	0.071	0.078	0.025	0.027	0.021	0.028	0.034	0.158	0.097	0.106	0.086	0.108	0.127	1.000
33	0.067	0.076	0.104	0.080	0.095	0.025	0.028	0.036	0.031	0.043	0.153	0.096	0.108	0.138	0.116	0.151	1.000
34	0.067	0.075	0.137	0.084	0.103	0.025	0.029	0.048	0.032	0.047	0.149	0.093	0.106	0.172	0.117	0.159	1.000
35	0.067	0.072	0.143	0.083	0.102	0.024	0.028	0.049	0.031	0.044	0.145	0.089	0.100	0.170	0.112	0.149	1.000
36	0.067	0.068	0.135	0.079	0.097	0.024	0.027	0.044	0.030	0.040	0.141	0.085	0.092	0.150	0.103	0.132	1.000
37	0.067	0.065	0.126	0.075	0.091	0.024	0.027	0.041	0.030	0.037	0.137	0.081	0.086	0.134	0.096	0.118	1.000
38	0.067	0.063	0.118	0.072	0.087	0.025	0.027	0.039	0.030	0.035	0.132	0.077	0.081	0.120	0.091	0.107	1.000
39	0.066	0.060	0.111	0.069	0.082	0.025	0.027	0.037	0.030	0.034	0.128	0.074	0.077	0.109	0.086	0.098	1.000
40	0.065	0.058	0.104	0.066	0.078	0.026	0.027	0.036	0.030	0.033	0.124	0.071	0.074	0.100	0.082	0.091	1.000
41	0.064	0.056	0.098	0.064	0.074	0.026	0.028	0.035	0.031	0.033	0.120	0.068	0.072	0.092	0.079	0.085	1.000
42	0.063	0.054	0.092	0.062	0.071	0.026	0.028	0.035	0.031	0.033	0.115	0.066	0.070	0.087	0.077	0.080	1.000
43	0.061	0.053	0.088	0.061	0.069	0.027	0.029	0.035	0.032	0.033	0.112	0.064	0.069	0.083	0.075	0.077	1.000
44	0.061	0.052	0.084	0.061	0.067	0.028	0.031	0.035	0.033	0.033	0.108	0.062	0.069	0.080	0.075	0.075	1.000
45	0.060	0.052	0.082	0.061	0.066	0.028	0.032	0.036	0.035	0.034	0.105	0.061	0.069	0.078	0.075	0.073	1.000
46	0.060	0.053	0.081	0.062	0.066	0.029	0.034	0.037	0.036	0.035	0.101	0.060	0.070	0.077	0.075	0.072	1.000
47	0.060	0.054	0.080	0.063	0.066	0.030	0.035	0.038	0.038	0.036	0.097	0.059	0.070	0.076	0.075	0.071	1.000
48	0.060	0.054	0.079	0.063	0.065	0.031	0.037	0.040	0.040	0.037	0.095	0.058	0.071	0.075	0.075	0.070	1.000
49	0.059	0.055	0.077	0.064	0.065	0.032	0.040	0.042	0.042	0.039	0.092	0.056	0.071	0.074	0.074	0.069	1.000
50	0.059	0.055	0.076	0.064	0.064	0.033	0.042	0.044	0.044	0.040	0.089	0.056	0.072	0.074	0.074	0.068	1.000
51	0.058	0.055	0.075	0.064	0.063	0.034	0.044	0.046	0.045	0.042	0.086	0.055	0.072	0.073	0.074	0.067	1.000
52	0.058	0.055	0.074	0.064	0.063	0.035	0.045	0.048	0.047	0.043	0.084	0.054	0.072	0.073	0.073	0.067	1.000
53	0.057	0.055	0.073	0.064	0.062	0.036	0.047	0.049	0.048	0.044	0.081	0.053	0.073	0.073	0.073	0.066	1.000
54	0.057	0.055	0.072	0.064	0.061	0.037	0.049	0.051	0.049	0.045	0.079	0.052	0.073	0.073	0.072	0.065	1.000
55	0.056	0.055	0.071	0.063	0.060	0.037	0.050	0.053	0.050	0.045	0.077	0.051	0.073	0.074	0.071	0.064	1.000
56	0.055	0.054	0.070	0.063	0.060	0.037	0.051	0.054	0.051	0.046	0.075	0.051	0.073	0.074	0.071	0.063	1.000
57	0.054	0.054	0.069	0.062	0.058	0.038	0.052	0.055	0.051	0.046	0.073	0.050	0.073	0.074	0.070	0.063	1.000
58	0.053	0.053	0.068	0.061	0.057	0.038	0.053	0.056	0.052	0.047	0.072	0.050	0.073	0.075	0.069	0.062	1.000
59	0.052	0.052	0.067	0.059	0.055	0.039	0.053	0.058	0.052	0.047	0.070	0.049	0.073	0.075	0.068	0.061	1.000
60	0.050	0.051	0.065	0.058	0.054	0.039	0.054	0.060	0.053	0.048	0.069	0.048	0.072	0.076	0.067	0.060	1.000
61	0.049	0.049	0.064	0.056	0.052	0.040	0.055	0.061	0.053	0.048	0.067	0.048	0.072	0.076	0.066	0.060	1.000
62	0.047	0.048	0.063	0.055	0.051	0.041	0.056	0.063	0.054	0.049	0.066	0.047	0.071	0.077	0.065	0.059	1.000
63	0.046	0.047	0.062	0.053	0.049	0.041	0.056	0.065	0.054	0.050	0.065	0.047	0.071	0.077	0.064	0.058	1.000
64	0.045	0.046	0.061	0.052	0.048	0.042	0.057	0.067	0.055	0.051	0.064	0.046	0.070	0.077	0.063	0.058	1.000
65	0.044	0.045	0.061	0.051	0.047	0.043	0.057	0.068	0.055	0.051	0.063	0.046	0.069	0.078	0.061	0.057	1.000
66	0.044	0.044	0.060	0.050	0.046	0.043	0.057	0.070	0.055	0.051	0.062	0.045	0.068	0.078	0.060	0.057	1.000
67	0.044	0.044	0.060	0.049	0.046	0.043	0.056	0.071	0.054	0.051	0.061	0.045	0.067	0.079	0.059	0.056	1.000
68	0.043	0.043	0.061	0.048	0.045	0.043	0.056	0.071	0.053	0.051	0.060	0.044	0.066	0.079	0.058	0.056	1.000
69	0.043	0.042	0.061	0.047	0.045	0.043	0.055	0.072	0.052	0.051	0.059	0.044	0.064	0.080	0.056	0.055	1.000
70	0.042	0.041	0.060	0.046	0.044	0.042	0.054	0.072	0.051	0.050	0.058	0.044	0.063	0.080	0.055	0.055	1.000
71	0.042	0.040	0.060	0.045	0.043	0.042	0.052	0.073	0.050	0.050	0.057	0.043	0.062	0.081	0.054	0.054	1.000
72	0.041	0.038	0.060	0.044	0.043	0.042	0.051	0.073	0.049	0.049	0.056	0.043	0.060	0.081	0.052	0.054	1.000
73	0.041	0.037	0.061	0.043	0.042	0.042	0.050	0.074	0.048	0.049	0.055	0.042	0.059	0.082	0.051	0.054	1.000
74	0.040	0.036	0.061	0.042	0.041	0.042	0.049	0.075	0.047	0.049	0.055	0.042	0.057	0.082	0.050	0.053	1.000
75	0.040	0.035	0.061	0.041	0.041	0.042	0.048	0.075	0.046	0.049	0.054	0.042	0.055	0.083	0.048	0.053	1.000
76	0.040	0.034	0.062	0.040	0.040	0.042	0.046	0.077	0.045	0.049	0.053	0.041	0.054	0.083	0.047	0.053	1.000
77	0.040	0.033	0.062	0.039	0.040	0.042	0.045	0.078	0.045	0.050	0.052	0.041	0.052	0.084	0.045	0.053	1.000
78	0.039	0.032	0.063	0.038	0.040	0.042	0.044	0.080	0.044	0.050	0.051	0.041	0.050	0.084	0.044	0.053	1.000
79	0.039	0.030	0.063	0.037	0.039	0.043	0.043	0.081	0.043	0.050	0.050	0.041	0.048	0.085	0.043	0.053	1.000

Table S6. TN predicted proportions by age at cancer diagnosis and ethnicity, in PV carriers and in the general population. BCAC data from BRIDGES

	ATM						PALB2						CHEK2					
Age at diagnosis	BCAC	White	Black	East Asian	South Asian	Mixed	BCAC	White	Black	East Asian	South Asian	Mixed	BCAC	White	Black	East Asian	South Asian	Mixed
30	0.121	0.119	0.109	0.124	0.177	0.156	0.298	0.312	0.292	0.342	0.422	0.391	0.087	0.088	0.081	0.096	0.135	0.119
31	0.117	0.125	0.117	0.122	0.159	0.161	0.291	0.323	0.306	0.331	0.386	0.391	0.084	0.094	0.087	0.094	0.120	0.122
32	0.112	0.127	0.122	0.117	0.143	0.161	0.283	0.326	0.315	0.310	0.355	0.381	0.082	0.096	0.092	0.089	0.109	0.121
33	0.108	0.125	0.124	0.109	0.131	0.156	0.275	0.323	0.319	0.281	0.330	0.364	0.080	0.095	0.095	0.081	0.099	0.116
34	0.103	0.119	0.124	0.099	0.122	0.145	0.268	0.313	0.318	0.253	0.311	0.344	0.077	0.092	0.095	0.073	0.093	0.109
35	0.099	0.110	0.120	0.088	0.114	0.131	0.260	0.295	0.312	0.232	0.299	0.322	0.075	0.086	0.093	0.066	0.088	0.100
36	0.095	0.100	0.114	0.079	0.109	0.117	0.252	0.276	0.304	0.215	0.289	0.300	0.073	0.079	0.090	0.060	0.085	0.090
37	0.091	0.092	0.110	0.071	0.104	0.106	0.244	0.258	0.296	0.200	0.281	0.280	0.071	0.073	0.087	0.055	0.082	0.083
38	0.087	0.085	0.106	0.065	0.099	0.096	0.237	0.242	0.289	0.187	0.272	0.262	0.068	0.068	0.085	0.051	0.080	0.076
39	0.083	0.079	0.103	0.060	0.096	0.089	0.229	0.228	0.282	0.176	0.265	0.247	0.066	0.064	0.084	0.048	0.077	0.071
40	0.079	0.073	0.100	0.056	0.092	0.083	0.221	0.216	0.277	0.168	0.258	0.234	0.064	0.060	0.082	0.045	0.075	0.067
41	0.076	0.069	0.098	0.053	0.089	0.078	0.213	0.206	0.273	0.161	0.252	0.224	0.061	0.057	0.081	0.044	0.074	0.064
42	0.072	0.065	0.096	0.051	0.087	0.074	0.205	0.197	0.269	0.157	0.247	0.216	0.059	0.055	0.080	0.042	0.072	0.062
43	0.070	0.062	0.094	0.050	0.085	0.071	0.199	0.189	0.266	0.154	0.242	0.209	0.058	0.053	0.080	0.042	0.071	0.060
44	0.067	0.060	0.094	0.050	0.083	0.069	0.194	0.183	0.264	0.153	0.238	0.205	0.056	0.051	0.080	0.042	0.071	0.059
45	0.065	0.058	0.093	0.050	0.081	0.068	0.189	0.178	0.263	0.154	0.235	0.202	0.055	0.050	0.080	0.043	0.070	0.058
46	0.063	0.056	0.093	0.050	0.080	0.067	0.184	0.174	0.262	0.155	0.232	0.200	0.054	0.049	0.081	0.043	0.069	0.058
47	0.060	0.055	0.093	0.051	0.079	0.066	0.179	0.171	0.262	0.157	0.229	0.198	0.052	0.048	0.081	0.044	0.069	0.058
48	0.059	0.054	0.093	0.052	0.078	0.066	0.176	0.167	0.262	0.160	0.226	0.197	0.052	0.048	0.082	0.046	0.069	0.058
49	0.058	0.053	0.093	0.053	0.076	0.065	0.174	0.164	0.262	0.162	0.223	0.196	0.051	0.047	0.083	0.047	0.068	0.058
50	0.057	0.052	0.093	0.054	0.075	0.065	0.171	0.162	0.262	0.165	0.220	0.195	0.051	0.047	0.084	0.048	0.068	0.058
51	0.056	0.051	0.093	0.055	0.074	0.064	0.168	0.159	0.263	0.167	0.218	0.194	0.050	0.046	0.085	0.050	0.067	0.059
52	0.055	0.050	0.094	0.056	0.073	0.064	0.166	0.157	0.264	0.171	0.215	0.193	0.050	0.046	0.086	0.051	0.067	0.059
53	0.054	0.049	0.094	0.057	0.072	0.064	0.164	0.155	0.265	0.174	0.213	0.192	0.050	0.046	0.087	0.053	0.067	0.059
54	0.053	0.048	0.095	0.058	0.071	0.063	0.163	0.154	0.266	0.177	0.211	0.192	0.050	0.046	0.089	0.055	0.067	0.060
55	0.053	0.048	0.096	0.060	0.071	0.063	0.162	0.152	0.268	0.181	0.209	0.192	0.050	0.046	0.090	0.056	0.067	0.060
56	0.052	0.048	0.097	0.061	0.070	0.063	0.160	0.151	0.270	0.185	0.207	0.192	0.049	0.046	0.092	0.058	0.067	0.061
57	0.051	0.047	0.097	0.063	0.069	0.063	0.159	0.150	0.272	0.189	0.206	0.192	0.049	0.046	0.094	0.061	0.066	0.061
58	0.051	0.047	0.098	0.065	0.068	0.064	0.158	0.149	0.274	0.194	0.204	0.193	0.049	0.046	0.095	0.063	0.067	0.062
59	0.050	0.047	0.099	0.066	0.068	0.064	0.157	0.149	0.276	0.197	0.203	0.194	0.049	0.046	0.097	0.065	0.067	0.063
60	0.050	0.046	0.100	0.068	0.067	0.064	0.156	0.148	0.278	0.201	0.202	0.195	0.050	0.047	0.099	0.067	0.067	0.064
61	0.049	0.046	0.101	0.069	0.067	0.065	0.154	0.148	0.280	0.205	0.201	0.196	0.050	0.047	0.101	0.069	0.067	0.065
62	0.049	0.046	0.102	0.071	0.067	0.065	0.153	0.148	0.282	0.209	0.200	0.197	0.050	0.047	0.103	0.071	0.067	0.066
63	0.049	0.046	0.103	0.072	0.066	0.066	0.152	0.148	0.285	0.213	0.200	0.199	0.050	0.048	0.104	0.074	0.068	0.067
64	0.048	0.046	0.104	0.074	0.066	0.066	0.151	0.149	0.287	0.216	0.199	0.201	0.050	0.048	0.106	0.076	0.068	0.069
65	0.047	0.047	0.105	0.075	0.066	0.067	0.149	0.149	0.289	0.220	0.199	0.203	0.050	0.049	0.108	0.078	0.069	0.070
66	0.047	0.047	0.106	0.077	0.066	0.068	0.148	0.150	0.292	0.223	0.199	0.205	0.049	0.050	0.111	0.080	0.069	0.072
67	0.046	0.047	0.107	0.078	0.066	0.069	0.147	0.151	0.294	0.227	0.199	0.208	0.049	0.051	0.113	0.082	0.070	0.073
68	0.046	0.047	0.108	0.080	0.066	0.070	0.145	0.152	0.297	0.230	0.199	0.211	0.049	0.051	0.115	0.084	0.071	0.075
69	0.045	0.048	0.109	0.081	0.066	0.071	0.144	0.153	0.299	0.233	0.200	0.214	0.049	0.052	0.117	0.087	0.071	0.077
70	0.045	0.048	0.110	0.082	0.066	0.073	0.142	0.155	0.302	0.236	0.200	0.217	0.049	0.053	0.119	0.089	0.072	0.079
71	0.044	0.049	0.111	0.083	0.066	0.074	0.141	0.156	0.305	0.238	0.201	0.221	0.049	0.055	0.122	0.091	0.073	0.082
72	0.044	0.050	0.112	0.085	0.066	0.076	0.140	0.158	0.308	0.241	0.202	0.225	0.049	0.056	0.124	0.093	0.074	0.084
73	0.043	0.050	0.114	0.086	0.067	0.077	0.138	0.160	0.310	0.243	0.203	0.229	0.049	0.057	0.127	0.095	0.075	0.087
74	0.043	0.051	0.115	0.087	0.067	0.079	0.137	0.162	0.313	0.246	0.204	0.234	0.049	0.059	0.129	0.097	0.076	0.090
75	0.042	0.052	0.116	0.088	0.068	0.081	0.135	0.165	0.316	0.248	0.206	0.239	0.049	0.060	0.132	0.099	0.078	0.093
76	0.042	0.053	0.117	0.089	0.068	0.083	0.134	0.167	0.319	0.250	0.207	0.244	0.048	0.062	0.134	0.101	0.079	0.096
77	0.041	0.054	0.118	0.089	0.069	0.086	0.132	0.170	0.322	0.252	0.209	0.250	0.048	0.064	0.137	0.102	0.081	0.100
78	0.041	0.055	0.120	0.090	0.069	0.088	0.131	0.174	0.325	0.254	0.211	0.256	0.048	0.066	0.139	0.104	0.082	0.104
79	0.040	0.056	0.121	0.091	0.070	0.091	0.130	0.177	0.328	0.255	0.213	0.262	0.048	0.068	0.142	0.106	0.084	0.108

	RAD51C						RAD51D						BARD1					
Age at diagnosis	BCAC	White	Black	East Asian	South Asian	Mixed	BCAC	White	Black	East Asian	South Asian	Mixed	BCAC	White	Black	East Asian	South Asian	Mixed
30	0.556	0.578	0.556	0.620	0.689	0.664	0.495	0.501	0.477	0.524	0.617	0.583	0.577	0.605	0.584	0.654	0.713	0.691
31	0.547	0.590	0.571	0.606	0.655	0.660	0.485	0.514	0.495	0.516	0.583	0.588	0.569	0.616	0.598	0.637	0.679	0.684
32	0.538	0.594	0.581	0.577	0.624	0.647	0.475	0.518	0.506	0.498	0.552	0.583	0.560	0.621	0.607	0.605	0.648	0.668
33	0.530	0.591	0.585	0.538	0.597	0.628	0.465	0.514	0.512	0.470	0.526	0.569	0.552	0.617	0.611	0.562	0.621	0.648
34	0.521	0.580	0.585	0.499	0.576	0.607	0.455	0.502	0.510	0.439	0.504	0.548	0.544	0.607	0.610	0.521	0.601	0.627
35	0.511	0.560	0.578	0.471	0.562	0.584	0.444	0.480	0.502	0.409	0.488	0.521	0.535	0.588	0.605	0.492	0.588	0.606
36	0.501	0.537	0.569	0.448	0.552	0.561	0.433	0.456	0.491	0.383	0.475	0.492	0.525	0.566	0.597	0.472	0.578	0.584
37	0.491	0.515	0.561	0.428	0.542	0.538	0.422	0.433	0.481	0.360	0.463	0.465	0.516	0.545	0.589	0.453	0.569	0.564
38	0.481	0.495	0.553	0.409	0.532	0.518	0.411	0.411	0.471	0.339	0.452	0.442	0.506	0.525	0.582	0.435	0.561	0.544
39	0.471	0.476	0.546	0.393	0.523	0.499	0.400	0.392	0.463	0.322	0.442	0.421	0.496	0.506	0.575	0.420	0.552	0.527
40	0.460	0.459	0.540	0.380	0.515	0.482	0.388	0.375	0.456	0.308	0.433	0.403	0.486	0.489	0.570	0.407	0.545	0.511
41	0.449	0.443	0.534	0.370	0.508	0.468	0.376	0.360	0.450	0.297	0.425	0.388	0.475	0.474	0.565	0.397	0.538	0.498
42	0.437	0.430	0.530	0.362	0.501	0.457	0.364	0.347	0.445	0.289	0.418	0.376	0.463	0.461	0.561	0.390	0.531	0.487
43	0.430	0.418	0.527	0.358	0.495	0.448	0.357	0.336	0.441	0.285	0.411	0.366	0.456	0.450	0.557	0.386	0.525	0.478
44	0.422	0.409	0.524	0.356	0.489	0.441	0.349	0.327	0.439	0.283	0.406	0.359	0.449	0.440	0.555	0.384	0.520	0.472
45	0.414	0.401	0.523	0.357	0.484	0.437	0.341	0.320	0.437	0.284	0.401	0.355	0.442	0.432	0.553	0.386	0.515	0.467
46	0.406	0.395	0.522	0.361	0.480	0.434	0.333	0.313	0.437	0.286	0.397	0.352	0.434	0.426	0.552	0.389	0.511	0.465
47	0.398	0.389	0.521	0.364	0.476	0.432	0.324	0.308	0.436	0.289	0.393	0.350	0.426	0.420	0.552	0.393	0.506	0.462
48	0.394	0.383	0.521	0.368	0.472	0.430	0.320	0.303	0.436	0.292	0.389	0.348	0.422	0.414	0.552	0.397	0.502	0.460
49	0.390	0.378	0.521	0.372	0.468	0.428	0.316	0.298	0.436	0.296	0.385	0.346	0.419	0.409	0.552	0.402	0.498	0.459
50	0.386	0.373	0.522	0.377	0.464	0.426	0.312	0.294	0.437	0.300	0.381	0.344	0.415	0.405	0.552	0.406	0.495	0.457
51	0.382	0.369	0.522	0.382	0.460	0.425	0.307	0.290	0.438	0.305	0.378	0.343	0.411	0.401	0.553	0.411	0.491	0.456
52	0.378	0.366	0.523	0.387	0.457	0.424	0.303	0.286	0.439	0.309	0.374	0.342	0.407	0.397	0.554	0.417	0.488	0.455
53	0.376	0.363	0.525	0.393	0.454	0.423	0.301	0.283	0.441	0.314	0.371	0.341	0.405	0.394	0.555	0.422	0.485	0.454
54	0.374	0.360	0.527	0.398	0.451	0.423	0.298	0.281	0.442	0.320	0.368	0.340	0.403	0.391	0.557	0.428	0.482	0.454
55	0.371	0.357	0.529	0.405	0.448	0.423	0.296	0.279	0.444	0.325	0.365	0.340	0.400	0.389	0.559	0.435	0.479	0.454
56	0.369	0.356	0.531	0.411	0.445	0.423	0.293	0.277	0.447	0.332	0.363	0.340	0.398	0.387	0.561	0.441	0.476	0.454
57	0.367	0.354	0.533	0.418	0.443	0.423	0.291	0.275	0.449	0.337	0.360	0.340	0.396	0.385	0.563	0.448	0.474	0.455
58	0.365	0.353	0.536	0.424	0.441	0.424	0.289	0.274	0.452	0.343	0.358	0.341	0.395	0.384	0.566	0.454	0.472	0.456
59	0.363	0.352	0.538	0.430	0.439	0.425	0.287	0.273	0.454	0.349	0.356	0.342	0.393	0.383	0.568	0.460	0.470	0.457
60	0.362	0.351	0.541	0.436	0.437	0.427	0.286	0.272	0.457	0.355	0.354	0.343	0.391	0.382	0.571	0.466	0.469	0.459
61	0.360	0.351	0.544	0.442	0.436	0.429	0.284	0.272	0.459	0.361	0.353	0.345	0.390	0.382	0.574	0.472	0.467	0.461
62	0.358	0.351	0.546	0.447	0.435	0.431	0.282	0.272	0.462	0.366	0.352	0.347	0.388	0.382	0.577	0.478	0.466	0.463
63	0.356	0.351	0.549	0.453	0.434	0.434	0.280	0.272	0.465	0.371	0.351	0.349	0.386	0.382	0.579	0.483	0.466	0.466
64	0.354	0.352	0.552	0.458	0.434	0.437	0.278	0.273	0.467	0.376	0.350	0.352	0.383	0.383	0.582	0.488	0.465	0.469
65	0.351	0.353	0.555	0.463	0.434	0.440	0.275	0.273	0.470	0.381	0.350	0.355	0.381	0.384	0.585	0.493	0.465	0.472
66	0.349	0.354	0.558	0.468	0.434	0.444	0.273	0.274	0.473	0.386	0.349	0.358	0.379	0.385	0.588	0.498	0.465	0.476
67	0.347	0.355	0.561	0.473	0.434	0.448	0.271	0.276	0.476	0.391	0.349	0.362	0.377	0.387	0.591	0.503	0.466	0.480
68	0.344	0.357	0.564	0.477	0.434	0.452	0.269	0.277	0.479	0.395	0.350	0.366	0.374	0.389	0.595	0.507	0.467	0.484
69	0.342	0.360	0.568	0.481	0.435	0.457	0.266	0.279	0.482	0.399	0.350	0.370	0.371	0.392	0.598	0.511	0.467	0.489
70	0.339	0.362	0.571	0.485	0.436	0.462	0.264	0.281	0.485	0.403	0.351	0.375	0.369	0.394	0.601	0.515	0.469	0.494
71	0.337	0.365	0.574	0.489	0.438	0.467	0.261	0.284	0.488	0.407	0.352	0.380	0.366	0.397	0.604	0.519	0.470	0.500
72	0.334	0.368	0.577	0.492	0.439	0.473	0.259	0.287	0.491	0.410	0.353	0.385	0.364	0.401	0.608	0.522	0.472	0.506
73	0.331	0.372	0.581	0.495	0.441	0.479	0.257	0.290	0.494	0.414	0.354	0.391	0.361	0.404	0.611	0.525	0.474	0.512
74	0.329	0.376	0.584	0.499	0.443	0.485	0.254	0.293	0.497	0.417	0.356	0.397	0.359	0.408	0.615	0.528	0.476	0.518
75	0.326	0.380	0.587	0.501	0.445	0.492	0.252	0.297	0.500	0.420	0.358	0.404	0.356	0.413	0.618	0.531	0.478	0.525
76	0.324	0.385	0.591	0.504	0.448	0.500	0.250	0.301	0.503	0.422	0.360	0.411	0.353	0.418	0.622	0.533	0.481	0.532
77	0.321	0.390	0.594	0.506	0.451	0.507	0.247	0.305	0.506	0.425	0.362	0.418	0.351	0.423	0.625	0.536	0.484	0.540
78	0.318	0.395	0.598	0.509	0.454	0.515	0.245	0.310	0.510	0.427	0.365	0.426	0.348	0.428	0.629	0.538	0.487	0.548
79	0.316	0.401	0.601	0.511	0.457	0.523	0.242	0.315	0.513	0.430	0.367	0.434	0.345	0.434	0.632	0.540	0.491	0.556

	BRCA1					BRCA2					Population						
Age at diagnosis	White	Black	East Asian	South Asian	Mixed	White	Black	East Asian	South Asian	Mixed	BCAC	White	Black	East Asian	South Asian	Mixed	NA
30	0.654	0.633	0.695	0.754	0.732	0.092	0.084	0.094	0.139	0.121	0.208	0.214	0.199	0.233	0.305	0.277	1.000
31	0.671	0.654	0.688	0.729	0.733	0.100	0.093	0.096	0.128	0.130	0.202	0.223	0.210	0.226	0.275	0.279	1.000
32	0.684	0.672	0.669	0.709	0.728	0.105	0.102	0.097	0.120	0.137	0.196	0.226	0.218	0.212	0.250	0.273	1.000
33	0.692	0.686	0.641	0.695	0.720	0.109	0.109	0.096	0.116	0.140	0.190	0.223	0.221	0.193	0.231	0.261	1.000
34	0.693	0.696	0.614	0.688	0.711	0.110	0.114	0.093	0.113	0.137	0.183	0.215	0.220	0.173	0.216	0.245	1.000
35	0.687	0.702	0.598	0.686	0.702	0.107	0.117	0.088	0.112	0.130	0.177	0.201	0.215	0.157	0.205	0.226	1.000
36	0.677	0.704	0.587	0.687	0.691	0.104	0.119	0.083	0.113	0.123	0.171	0.186	0.208	0.143	0.197	0.207	1.000
37	0.666	0.704	0.577	0.687	0.681	0.103	0.123	0.080	0.116	0.119	0.165	0.172	0.201	0.132	0.190	0.191	1.000
38	0.655	0.705	0.567	0.686	0.671	0.103	0.129	0.080	0.121	0.118	0.159	0.160	0.195	0.122	0.183	0.177	1.000
39	0.644	0.704	0.559	0.684	0.661	0.105	0.136	0.081	0.127	0.118	0.153	0.150	0.190	0.114	0.178	0.165	1.000
40	0.633	0.703	0.551	0.682	0.651	0.107	0.144	0.083	0.133	0.120	0.147	0.141	0.186	0.108	0.172	0.155	1.000
41	0.621	0.702	0.544	0.678	0.642	0.108	0.151	0.085	0.139	0.122	0.141	0.133	0.183	0.103	0.168	0.147	1.000
42	0.611	0.700	0.539	0.674	0.634	0.111	0.159	0.088	0.145	0.124	0.135	0.127	0.180	0.100	0.164	0.141	1.000
43	0.603	0.700	0.538	0.672	0.630	0.113	0.167	0.092	0.151	0.128	0.131	0.122	0.177	0.098	0.160	0.136	1.000
44	0.599	0.702	0.542	0.672	0.628	0.117	0.176	0.097	0.158	0.133	0.127	0.117	0.176	0.097	0.157	0.133	1.000
45	0.596	0.705	0.549	0.672	0.629	0.120	0.185	0.103	0.163	0.138	0.123	0.114	0.175	0.098	0.154	0.131	1.000
46	0.594	0.708	0.557	0.672	0.631	0.122	0.193	0.110	0.168	0.143	0.119	0.111	0.175	0.099	0.152	0.129	1.000
47	0.592	0.711	0.564	0.671	0.632	0.125	0.201	0.117	0.174	0.149	0.116	0.109	0.174	0.100	0.150	0.128	1.000
48	0.589	0.712	0.570	0.670	0.632	0.129	0.211	0.125	0.180	0.155	0.114	0.106	0.174	0.101	0.148	0.127	1.000
49	0.585	0.713	0.576	0.667	0.632	0.134	0.222	0.134	0.187	0.162	0.112	0.104	0.175	0.103	0.146	0.126	1.000
50	0.580	0.713	0.581	0.664	0.630	0.139	0.233	0.143	0.194	0.170	0.110	0.102	0.175	0.105	0.144	0.125	1.000
51	0.575	0.713	0.584	0.660	0.628	0.143	0.243	0.153	0.200	0.176	0.108	0.101	0.175	0.107	0.142	0.125	1.000
52	0.569	0.712	0.588	0.655	0.625	0.147	0.253	0.162	0.206	0.183	0.106	0.099	0.176	0.109	0.140	0.124	1.000
53	0.565	0.712	0.592	0.651	0.623	0.152	0.263	0.172	0.211	0.190	0.105	0.098	0.177	0.111	0.139	0.124	1.000
54	0.561	0.713	0.597	0.647	0.622	0.155	0.273	0.182	0.216	0.196	0.104	0.097	0.178	0.114	0.137	0.124	1.000
55	0.558	0.714	0.603	0.645	0.622	0.158	0.281	0.191	0.219	0.201	0.103	0.096	0.179	0.116	0.136	0.124	1.000
56	0.554	0.714	0.607	0.640	0.620	0.160	0.288	0.199	0.221	0.205	0.102	0.095	0.181	0.119	0.134	0.124	1.000
57	0.547	0.712	0.609	0.633	0.616	0.163	0.295	0.207	0.224	0.209	0.101	0.094	0.182	0.122	0.133	0.124	1.000
58	0.538	0.708	0.608	0.624	0.610	0.166	0.303	0.216	0.227	0.214	0.100	0.094	0.183	0.125	0.132	0.124	1.000
59	0.527	0.703	0.605	0.614	0.602	0.170	0.311	0.226	0.231	0.220	0.099	0.093	0.185	0.128	0.131	0.125	1.000
60	0.516	0.697	0.601	0.603	0.594	0.174	0.320	0.236	0.235	0.227	0.099	0.093	0.187	0.131	0.130	0.125	1.000
61	0.506	0.691	0.598	0.593	0.586	0.178	0.330	0.246	0.240	0.234	0.098	0.093	0.188	0.133	0.130	0.126	1.000
62	0.497	0.686	0.595	0.584	0.580	0.183	0.340	0.257	0.246	0.242	0.097	0.093	0.190	0.136	0.129	0.127	1.000
63	0.490	0.683	0.594	0.576	0.576	0.189	0.350	0.268	0.252	0.251	0.096	0.093	0.192	0.139	0.129	0.128	1.000
64	0.486	0.682	0.595	0.571	0.574	0.195	0.361	0.280	0.258	0.260	0.095	0.093	0.193	0.141	0.128	0.129	1.000
65	0.485	0.683	0.598	0.569	0.576	0.201	0.371	0.290	0.263	0.268	0.094	0.094	0.195	0.144	0.128	0.131	1.000
66	0.487	0.686	0.603	0.570	0.580	0.205	0.378	0.298	0.267	0.275	0.093	0.094	0.197	0.146	0.128	0.132	1.000
67	0.491	0.691	0.610	0.573	0.586	0.209	0.384	0.305	0.270	0.281	0.092	0.095	0.199	0.149	0.128	0.134	1.000
68	0.496	0.696	0.616	0.576	0.593	0.212	0.389	0.311	0.272	0.287	0.091	0.095	0.201	0.151	0.128	0.136	1.000
69	0.500	0.700	0.622	0.578	0.599	0.214	0.394	0.316	0.274	0.292	0.090	0.096	0.203	0.153	0.129	0.139	1.000
70	0.504	0.703	0.626	0.580	0.605	0.217	0.398	0.320	0.276	0.297	0.089	0.097	0.205	0.155	0.129	0.141	1.000
71	0.508	0.707	0.630	0.582	0.611	0.220	0.401	0.325	0.277	0.302	0.088	0.098	0.207	0.157	0.129	0.144	1.000
72	0.513	0.710	0.635	0.585	0.617	0.223	0.406	0.329	0.279	0.308	0.087	0.099	0.209	0.159	0.130	0.146	1.000
73	0.518	0.715	0.639	0.589	0.625	0.227	0.411	0.333	0.282	0.315	0.086	0.101	0.211	0.161	0.131	0.150	1.000
74	0.526	0.720	0.644	0.593	0.634	0.232	0.417	0.339	0.286	0.323	0.085	0.102	0.213	0.163	0.132	0.153	1.000
75	0.534	0.726	0.650	0.599	0.643	0.238	0.423	0.344	0.291	0.332	0.085	0.104	0.215	0.165	0.132	0.156	1.000
76	0.543	0.732	0.656	0.606	0.654	0.245	0.431	0.351	0.297	0.343	0.084	0.106	0.217	0.166	0.134	0.160	1.000
77	0.554	0.739	0.662	0.614	0.665	0.253	0.440	0.358	0.304	0.355	0.083	0.108	0.219	0.168	0.135	0.164	1.000
78	0.565	0.746	0.669	0.622	0.677	0.262	0.449	0.365	0.311	0.368	0.082	0.110	0.222	0.169	0.136	0.169	1.000
79	0.576	0.753	0.675	0.630	0.689	0.271	0.457	0.372	0.319	0.380	0.081	0.112	0.224	0.170	0.137	0.174	1.000
