# Supplementary material for: Adapting the BOADICEA breast and ovarian cancer risk models for the ethnically diverse UK population
Source: Br J Cancer. 2025 Jul 17;133(6):844–55. doi: 10.1038/s41416-025-03117-y (PMC12449465; doi:10.1038/s41416-025-03117-y)
Supplement: Supplementary file 4 — Supplementary Materials - tables S7-S15 [file 41416_2025_3117_MOESM4_ESM.pdf]

Table S7. SNPs and weights included in the construction of the PGS models. There are three surrogates correlated with original SNP from Mavaddat et al. 2019 at r2>0.9). The 307-SNP PGS does not include 22_29203724_C_T and 22_29551872_A_G. The 303-SNP PGS also excludes in addition SNPs 6_27425644_G_C, 15_46680811_C_A, 3_29294845_C_T, and 11_46318032_C_G. [a]SNP variant name; [b]Position based on build 37.

UKB rs number	SNP[a]	Chromosome	Position[b]	Reference Allele	Effect Allele	SNP weight
rs707475	1_7917076_G_A	1	7917076	G	A	-0.0409
rs616488	1_10566215_A_G	1	10566215	A	G	-0.0586
rs2992756	1_18807339_T_C	1	18807339	T	C	-0.0564
rs4233486	1_41380440_C_T	1	41380440	C	T	0.0426
rs114282204	1_41389220_T_C	1	41389220	T	C	0.1550
1.46670206_TC_T	1_46670206_TC_T	1	46670206	TC	T	0.0447
1.51467096_CT_C	1_51467096_CT_C	1	51467096	CT	C	0.0374
rs17426269	1_88156923_G_A	1	88156923	G	A	0.0494
rs2151842	1_88428199_C_A	1	88428199	C	A	-0.0387
rs612683	1_100880328_A_T	1	100880328	A	T	0.0373
1.110198129_CAAA_C	1_110198129_CAAA_C	1	110198129	CAAA	C	0.0458
rs7513707	1_114445880_G_A	1	114445880	G	A	0.0621
rs12406858	1_118141492_A_C	1	118141492	A	C	0.0452
rs637868	1_120257110_T_C	1	120257110	T	C	0.0385
rs11249433	1_121280613_A_G	1	121280613	A	G	0.0881
rs111458676	1_121287994_A_G	1	121287994	A	G	-0.0673
rs143384623	1_145604302_C_CT	1	145604302	C	CT	-0.0399
rs11205303	1_149906413_T_C	1	149906413	T	C	0.0548
rs12091730	1_155556971_G_A	1	155556971	G	A	0.0499
rs761575824	1_168171052_CA_C	1	168171052	CA	C	-0.0680
rs11463354	1_172328767_T_TA	1	172328767	T	TA	-0.0435
rs35383942	1_201437832_C_T	1	201437832	C	T	0.0917
rs6686987	1_202184600_C_T	1	202184600	C	T	-0.0065
rs7514172	1_203770448_T_A	1	203770448	T	A	0.0498
rs11268668	1_204502514_T_TTCTGAAACAGGG	1	204502514	T	TTCTGAAACAGGG	-0.0321
rs2785646	1_208076291_G_A	1	208076291	G	A	-0.0366
rs2576261	1_217053815_T_G	1	217053815	T	G	0.0417
rs11117758	1_217220574_G_A	1	217220574	G	A	-0.0440
rs11118563	1_220671050_C_T	1	220671050	C	T	0.0418
rs72755295	1_242034263_A_G	1	242034263	A	G	0.1428
rs78425380	2_10138983_T_C	2	10138983	T	C	0.0603
rs6743383	2_19315675_T_A	2	19315675	T	A	-0.0331
rs6725517	2_25129473_A_G	2	25129473	A	G	-0.0427
rs12472404	2_29179452_G_C	2	29179452	G	C	-0.0066
rs4322799	2_29615233_T_C	2	29615233	T	C	-0.0427
rs553796823	2_39699510_C_CT	2	39699510	C	CT	-0.0402
rs6756513	2_70172587_G_A	2	70172587	G	A	-0.0412
rs1036759	2_88358825_G_C	2	88358825	G	C	0.0473
rs6746250	2_121058254_A_G	2	121058254	A	G	-0.0334
rs17625845	2_121089731_T_C	2	121089731	T	C	-0.0427
rs10164550	2_121159205_G_A	2	121159205	G	A	-0.0440
rs10179592	2_121246568_T_C	2	121246568	T	C	0.0992
rs17726078	2_172974566_C_G	2	172974566	C	G	-0.0473
rs1550622	2_174212910_A_G	2	174212910	A	G	0.0593
rs2356656	2_192381934_C_T	2	192381934	C	T	0.0316
rs10197246	2_202204741_T_C	2	202204741	T	C	-0.0492
rs4442975	2_217920769_G_T	2	217920769	G	T	-0.1318
2.217955896_GA_G	2_217955896_GA_G	2	217955896	GA	G	-0.2016
rs11693806	2_218292158_C_G	2	218292158	C	G	-0.0757
rs3791977	2_218714845_G_A	2	218714845	G	A	-0.0431
rs4676356	2_241388857_C_A	2	241388857	C	A	-0.1232
rs6762558	3_4742251_A_G	3	4742251	A	G	0.0616
rs552647	3_27353716_C_A	3	27353716	C	A	0.0748
rs62255657	3_27388664_C_G	3	27388664	C	G	0.0502
rs112476261	3_29294845_C_T	3	29294845	C	T	-0.1281
rs17838698	3_30684907_C_T	3	30684907	C	T	0.0592
rs56387622	3_46888198_T_C	3	46888198	T	C	-0.0806
rs371314787	3_49709912_C_CT	3	49709912	C	CT	-0.0367
rs138866686	3_55970777_A_AT	3	55970777	A	AT	-0.1195
rs2886671	3_59373745_C_T	3	59373745	C	T	-0.0394
rs73130576 [c]	3_63887449_T_TTG	3	63887449	T	TTG	0.0648
rs9825432	3_71620370_T_G	3	71620370	T	G	-0.0374
rs13066793	3_87037543_A_G	3	87037543	A	G	-0.0723
rs639355	3_99403877_G_A	3	99403877	G	A	-0.0376
3.141112859_CTT_C	3_141112859_CTT_C	3	141112859	CTT	C	0.0551
rs58058861	3_172285237_G_A	3	172285237	G	A	0.0422
rs9882792	3_189774456_C_T	3	189774456	C	T	-0.0478
rs10012017	4_38784633_G_T	4	38784633	G	T	0.0489
rs17014016	4_89240476_G_A	4	89240476	G	A	0.0352
rs775780062	4_92594859_TTCTTTC_T	4	92594859	TTCTTTC	T	-0.0407
rs62331150	4_106069013_G_T	4	106069013	G	T	0.0471
rs56039025	4_143467195_C_T	4	143467195	C	T	-0.0569
rs745707748	4_151218296_CATATTT_C	4	151218296	CATATTT	C	0.0388
rs28436676	4_175842495_G_A	4	175842495	G	A	-0.0898
rs62334414	4_175847436_C_A	4	175847436	C	A	0.0348
4.187513623 TA T [c]	4_187503758_A_T	4	187503758	A	T	0.0357
rs62641919	5_345109_T_C	5	345109	T	C	0.0840
rs10069690	5_1279790_C_T	5	1279790	C	T	0.0617
rs3215401	5_1296255_A_AG	5	1296255	A	AG	-0.0549
rs62329727	5_1353077_T_C	5	1353077	T	C	0.1552
rs4866496	5_2777029_G_A	5	2777029	G	A	0.0391
rs17611291	5_16231194_G_C	5	16231194	G	C	-0.0426
rs770436441	5_32579616_TCA_T	5	32579616	TCA	T	0.0363
rs138335056	5_44508264_G_GT	5	44508264	G	GT	-0.1177
rs187108781	5_44619502_A_G	5	44619502	A	G	-0.1101
rs4613718	5_44649944_C_T	5	44649944	C	T	0.0492
rs10941679	5_44706498_A_G	5	44706498	A	G	0.0497
rs17343002	5_44853593_G_C	5	44853593	G	C	-0.0336
rs10940312 [c]	5_52679539_C_CA	5	52679539	C	CA	0.0571
rs553874618	5_55662540_C_CT	5	55662540	C	CT	-0.0458
rs889310	5_55965167_C_T	5	55965167	C	T	0.0394
rs16886165	5_56023083_T_G	5	56023083	T	G	0.1366
rs76250845	5_56042972_C_T	5	56042972	C	T	0.0865
rs11949391	5_56045081_T_C	5	56045081	T	C	-0.0564
rs113778879	5_58241712_C_T	5	58241712	C	T	-0.0434
rs3010266	5_71965007_G_A	5	71965007	G	A	-0.0410
rs157557	5_73234583_T_C	5	73234583	T	C	-0.0363
rs767431357	5_77155397_GT_G	5	77155397	GT	G	-0.0408
rs34525310	5_79180995_G_GA	5	79180995	G	GA	0.0328
5.81512947_TA_T	5_81512947_TA_T	5	81512947	TA	T	-0.0598
rs332529	5_90789470_G_A	5	90789470	G	A	-0.0564
rs17157372	5_104300273_G_T	5	104300273	G	T	-0.0487
rs335160	5_122478676_C_A	5	122478676	C	A	-0.0386
rs1428387	5_122705244_C_T	5	122705244	C	T	0.0944
rs6860806	5_131640536_A_G	5	131640536	A	G	0.0392
rs6596100	5_132407058_C_T	5	132407058	C	T	-0.0388
rs1432679	5_158244083_C_T	5	158244083	C	T	-0.0677
rs10074269	5_169591460_T_C	5	169591460	T	C	0.0412
rs6864691	5_173358154_G_A	5	173358154	G	A	0.0365
rs4868701	5_176134882_T_C	5	176134882	T	C	0.0363
rs418053	6_13713366_G_C	6	13713366	G	C	-0.0553
rs3819405	6_16399557_C_T	6	16399557	C	T	-0.0373
rs12211970	6_18783140_G_A	6	18783140	G	A	0.0326
rs769485514	6_20537845_CA_C	6	20537845	CA	C	-0.0391
rs9358466	6_21923810_T_C	6	21923810	T	C	-0.0321
rs34196306	6_27425644_G_C	6	27425644	G	C	-0.0737
rs111342015	6_43227141_G_A	6	43227141	G	A	-0.0640
rs10623112	6_82263549_AAT_A	6	82263549	AAT	A	0.0477
6.85912194_CAA_C	6_85912194_CAA_C	6	85912194	CAA	C	0.0762
rs73754909	6_87803819_T_C	6	87803819	T	C	0.0383
rs55941023	6_130341728_C_CT	6	130341728	C	CT	0.0472
rs2121348	6_149595505_T_C	6	149595505	T	C	-0.0476
rs6913578	6_151949806_A_C	6	151949806	A	C	0.0703
rs60954078	6_151955914_A_G	6	151955914	A	G	0.1449
6.152022664_CAAAAAAA_C	6_152022664_CAAAAAAA_C	6	152022664	CAAAAAAA	C	0.0137
rs851984	6_152023191_G_A	6	152023191	G	A	0.0626
rs6904031	6_152055978_A_T	6	152055978	A	T	0.0740
rs910416	6_152432902_C_T	6	152432902	C	T	0.0649
rs9364472	6_169006947_C_G	6	169006947	C	G	-0.0308
rs6940159	6_170332621_T_C	6	170332621	T	C	0.0373
rs7971	7_21940960_A_G	7	21940960	A	G	-0.0467
rs289997	7_25569548_C_T	7	25569548	C	T	-0.0486
rs74765302	7_28869017_G_A	7	28869017	G	A	-0.0572
rs13244925	7_55192256_A_C	7	55192256	A	C	-0.0349
rs17268829	7_94113799_T_C	7	94113799	T	C	0.0449
rs4439053	7_98005235_G_A	7	98005235	G	A	-0.0467
rs111963714	7_99948655_T_G	7	99948655	T	G	0.0420
rs71559437	7_101552440_G_A	7	101552440	G	A	-0.0568
rs7800548	7_102481842_T_C	7	102481842	T	C	0.0418
rs12706954	7_130656911_C_T	7	130656911	C	T	-0.0476
rs68056147	7_130674481_G_A	7	130674481	G	A	0.0416
rs201664599	7_139943702_CT_C	7	139943702	CT	C	0.0582
rs62485509	7_144048902_G_T	7	144048902	G	T	-0.0563
rs66823261	8_170692_T_C	8	170692	T	C	0.0477
8.17787610_CT_C	8_17787610_CT_C	8	17787610	CT	C	-0.0377
rs1028016	8_23447496_A_G	8	23447496	A	G	-0.0389
rs310295	8_23663653_C_A	8	23663653	C	A	0.0335
rs9693444	8_29509616_A_C	8	29509616	A	C	-0.0601
rs13365225	8_36858483_A_G	8	36858483	A	G	-0.076
rs1511243	8_76230943_A_G	8	76230943	A	G	0.0755
rs72658084	8_76333056_C_T	8	76333056	C	T	0.1129
rs1533366	8_76378165_G_T	8	76378165	G	T	-0.0391
rs62517052	8_102483100_T_C	8	102483100	T	C	0.0593
rs12546444	8_106358620_A_T	8	106358620	A	T	-0.0745
rs13267382	8_117209548_A_G	8	117209548	A	G	-0.0417
rs62526620	8_120862186_A_G	8	120862186	A	G	0.0527
rs35542655	8_124563705_T_C	8	124563705	T	C	0.0477
rs12541094	8_124571581_G_A	8	124571581	G	A	0.0340
rs7842619	8_124739913_T_G	8	124739913	T	G	0.0466
rs35961416	8_128213561_C_CA	8	128213561	C	CA	-0.0430
rs12550713	8_128370949_C_G	8	128370949	C	G	0.0642
rs10096351	8_128372172_A_G	8	128372172	A	G	0.0597
rs1016578	8_129199566_G_A	8	129199566	G	A	0.0615
rs7830152	8_143669254_A_G	8	143669254	A	G	-0.0346
rs10975870	9_6880263_A_G	9	6880263	A	G	0.0348
rs745322029	9_21964882_CAAAA_C	9	21964882	CAAAA	C	0.0550
rs17694493	9_22041998_C_G	9	22041998	C	G	0.0289
rs4880038	9_36928288_T_C	9	36928288	T	C	0.0249
rs665889	9_87782211_T_C	9	87782211	T	C	0.0361
rs10120432	9_98362587_T_C	9	98362587	T	C	0.0576
9.110303808_TAA_T	9_110303808_TAA_T	9	110303808	TAA	T	0.0797
rs10816625	9_110837073_A_G	9	110837073	A	G	0.1158
rs13294895	9_110837176_C_T	9	110837176	C	T	0.0653
rs7848334	9_110849525_G_T	9	110849525	G	T	0.0153
rs630965	9_110885479_C_T	9	110885479	C	T	0.0877
rs1895062	9_119313486_A_G	9	119313486	A	G	-0.0462
rs3861871	9_129424719_A_G	9	129424719	A	G	-0.0382
rs550057	9_136146597_C_T	9	136146597	C	T	0.0400
rs55910451	10_5794652_A_G	10	5794652	A	G	0.0470
rs10796139	10_13892298_G_A	10	13892298	G	A	0.0371
rs7072776	10_22032942_A_G	10	22032942	A	G	-0.0580
rs762131501	10_22477776_ACC_A	10	22477776	ACC	A	0.1687
rs10764337	10_22861490_A_C	10	22861490	A	C	0.0875
rs2384736	10_38523626_C_A	10	38523626	C	A	0.0404
rs10995201	10_64299890_A_G	10	64299890	A	G	-0.1345
rs6479868	10_64819996_G_T	10	64819996	G	T	0.0472
rs111833376	10_71335574_C_T	10	71335574	C	T	-0.0404
rs719338	10_80851257_G_T	10	80851257	G	T	-0.0805
rs4980029	10_80886726_A_G	10	80886726	A	G	0.0762
10.95292187_CAA_C	10_95292187_CAA_C	10	95292187	CAA	C	-0.0512
rs10885405	10_114777670_C_T	10	114777670	C	T	0.0472
rs12250948	10_115128491_T_C	10	115128491	T	C	-0.0592
rs9421410	10_123095209_G_A	10	123095209	G	A	-0.0538
rs45631580	10_123340107_A_G	10	123340107	A	G	0.1508
10.123340431_GC_G	10_123340431_GC_G	10	123340431	GC	G	-0.2408
rs45631563	10_123349324_A_T	10	123349324	A	T	-0.2609
rs7394715	11_433617_T_C	11	433617	T	C	-0.0437
rs6597981	11_803017_A_G	11	803017	A	G	0.0457
rs4980386	11_1895708_C_A	11	1895708	C	A	-0.0762
rs10832963	11_18664241_T_G	11	18664241	T	G	0.0461
rs4472923	11_42844441_C_T	11	42844441	C	T	-0.0336
rs10838267	11_44368892_G_A	11	44368892	G	A	0.0374
rs77047825	11_46318032_C_G	11	46318032	C	G	-0.0748
rs12287832	11_65553492_C_A	11	65553492	C	A	0.0425
rs10896047	11_65572431_G_A	11	65572431	G	A	-0.0347
rs35039974	11_69328130_A_T	11	69328130	A	T	-0.0423
rs661204	11_69330983_G_A	11	69330983	G	A	0.1022
rs78540526	11_69331418_C_T	11	69331418	C	T	0.1782
rs7125780	11_103614438_T_G	11	103614438	T	G	0.0147
rs199504893	11_108267402_C_CA	11	108267402	C	CA	-0.0022
rs610437	11_111696440_T_C	11	111696440	T	C	-0.0396
rs625145	11_116727936_A_T	11	116727936	A	T	-0.0423
rs7121616	11_122966626_A_G	11	122966626	A	G	-0.0383
rs7939702	11_129243417_T_G	11	129243417	T	G	-0.0543
rs11822830	11_129461016_A_G	11	129461016	A	G	0.0453
rs797736	12_293626_A_G	12	293626	A	G	0.0401
rs12422552	12_14413931_G_C	12	14413931	G	C	0.0484
rs788458	12_28149568_C_T	12	28149568	C	T	-0.0620
rs7297051	12_28174817_C_T	12	28174817	C	T	-0.0856
rs11049431	12_28347382_C_T	12	28347382	C	T	-0.0521
rs1027113	12_29140260_G_A	12	29140260	G	A	0.0647
rs2277339	12_57146069_T_G	12	57146069	T	G	-0.0579
rs2870876	12_70798355_A_T	12	70798355	A	T	0.0469
rs111622698	12_83064195_G_GA	12	83064195	G	GA	0.0671
rs10862899	12_85004551_C_T	12	85004551	C	T	0.0348
rs17356907	12_96027759_A_G	12	96027759	A	G	-0.0867
rs7132703	12_103097887_C_T	12	103097887	C	T	0.0546
rs11065822	12_111600134_G_T	12	111600134	G	T	-0.0442
rs1061657	12_115108136_T_C	12	115108136	T	C	0.0465
rs11067551	12_115796577_A_G	12	115796577	A	G	-0.0428
rs2454399	12_115835836_T_C	12	115835836	T	C	-0.0813
rs206966	12_120832146_C_T	12	120832146	C	T	0.0516
rs56404467	13_32839990_G_A	13	32839990	G	A	0.0424
rs11571833	13_32972626_A_T	13	32972626	A	T	0.2687
rs9315973	13_43501356_A_G	13	43501356	A	G	0.0517
rs12870942	13_73806982_T_C	13	73806982	T	C	0.0345
rs2181965	13_73960952_A_G	13	73960952	A	G	0.0399
rs34914085	14_37128564_C_A	14	37128564	C	A	-0.0733
rs2253012	14_37228504_C_T	14	37228504	C	T	0.0390
rs2588809	14_68660428_T_C	14	68660428	T	C	-0.0474
rs11624333	14_68979835_T_C	14	68979835	T	C	-0.0911
14.91751788_TC_T	14_91751788_TC_T	14	91751788	TC	T	0.0380
rs941764	14_91841069_A_G	14	91841069	A	G	0.0513
rs78440108	14_93070286_C_T	14	93070286	C	T	-0.0577
rs4983544	14_105213978_T_G	14	105213978	T	G	0.0399
rs187010898	15_46680811_C_A	15	46680811	C	A	-0.1973
rs4774565	15_50694306_A_G	15	50694306	A	G	-0.0417
rs8042593	15_66630569_G_A	15	66630569	G	A	-0.0369
rs35874463	15_67457698_A_G	15	67457698	A	G	0.0782
rs8035987	15_75750383_T_C	15	75750383	T	C	-0.0413
rs2290202	15_91512267_G_T	15	91512267	G	T	-0.0589
rs144767203	15_100905819_A_C	15	100905819	A	C	-0.0608
16.4008542_CAAAAA_C	16_4008542_CAAAAA_C	16	4008542	CAAAAA	C	-0.0329
rs11076805	16_4106788_C_A	16	4106788	C	A	-0.0300
rs12709163	16_6963972_C_G	16	6963972	C	G	0.0354
rs34872983	16_10706580_G_A	16	10706580	G	A	-0.0740
rs75753503	16_23007047_G_T	16	23007047	G	T	0.1218
rs35668161	16_52538825_C_A	16	52538825	C	A	0.1147
rs4784227	16_52599188_C_T	16	52599188	C	T	0.1070
rs55872725	16_53809123_C_T	16	53809123	C	T	-0.0704
rs6499648	16_53861139_C_T	16	53861139	C	T	-0.0338
rs7184573	16_53861592_G_A	16	53861592	G	A	-0.0337
rs28539243	16_54682064_G_A	16	54682064	G	A	0.0477
rs7500067	16_80648296_A_G	16	80648296	A	G	0.0839
rs9931038	16_85145977_T_C	16	85145977	T	C	-0.0211
rs12449271	16_87086492_T_C	16	87086492	T	C	-0.0469
rs79461387	17_29168077_G_T	17	29168077	G	T	-0.0568
rs150537328	17_39251123_T_C	17	39251123	T	C	0.0799
rs11296	17_40127060_T_C	17	40127060	T	C	0.0174
rs17881320	17_40485239_G_T	17	40485239	G	T	-0.0571
rs149370081	17_40744470_G_A	17	40744470	G	A	0.2017
rs545502941	17_43212339_C_CT	17	43212339	C	CT	0.0438
rs2668667	17_44283858_G_A	17	44283858	G	A	-0.0540
rs2787486	17_53209774_A_C	17	53209774	A	C	-0.0793
rs745570	17_77781725_A_G	17	77781725	A	G	-0.0401
rs16976596	18_11696613_C_T	18	11696613	C	T	-0.0381
rs11665269	18_20634253_C_T	18	20634253	C	T	-0.0415
rs1111207	18_24125857_T_C	18	24125857	T	C	0.0346
rs527616	18_24337424_C_G	18	24337424	C	G	0.0455
18.24518050_AT_A	18_24518050_AT_A	18	24518050	AT	A	-0.0599
rs8092192	18_25407513_C_G	18	25407513	C	G	0.0399
rs72931898	18_29981526_G_A	18	29981526	G	A	-0.1058
rs9954058	18_42411803_G_C	18	42411803	G	C	-0.0877
rs9952980	18_42888797_T_C	18	42888797	T	C	-0.0542
rs117922601	19_13249921_G_T	19	13249921	G	T	0.0956
rs56069439	19_17393925_C_A	19	17393925	C	A	0.0378
rs10164323	19_18569492_C_T	19	18569492	C	T	-0.0719
rs140702307	19_19517054_C_CGGGCG	19	19517054	C	CGGGCG	0.0437
rs56681946	19_44283031_T_C	19	44283031	T	C	0.0619
rs4399645	19_46166073_T_C	19	46166073	T	C	-0.0360
rs1172821	19_55816678_C_T	19	55816678	C	T	-0.0359
rs16991615	20_5948227_G_A	20	5948227	G	A	0.0760
rs1154723	20_11379842_T_C	20	11379842	T	C	0.0844
rs6030585	20_41613706_C_G	20	41613706	C	G	0.0315
rs13039563	20_52296849_G_A	20	52296849	G	A	0.0440
rs2822999	21_16364756_T_G	21	16364756	T	G	0.0646
rs2823130	21_16566350_A_G	21	16566350	A	G	0.0595
rs2403907	21_16574455_C_A	21	16574455	C	A	-0.0707
rs4818836	21_47762932_G_A	21	47762932	G	A	0.0946
rs9798754	22_19766137_C_T	22	19766137	C	T	-0.0367
rs17879961	22_29121087_A_G	22	29121087	A	G	0.1839
rs5997390	22_29135543_G_A	22	29135543	G	A	0.0654
rs34134147	22_29203724_C_T	22	29203724	C	T	0.1405
rs132289	22_29551872_A_G	22	29551872	A	G	-0.1716
rs5750715	22_39343916_T_A	22	39343916	T	A	0.0407
22.40904707_CT_C	22_40904707_CT_C	22	40904707	CT	C	0.1148
rs9611990	22_43433100_C_T	22	43433100	C	T	-0.0600
rs112855987	22_45319953_G_A	22	45319953	G	A	-0.0134
rs28512361	22_46283297_G_A	22	46283297	G	A	0.0736

Table S8. Allele frequencies (EAFs) by genetic ancestry for SNPs included in the PGS models. EAFs were estimated in UK Biobank individuals without a diagnosis of cancer or mastectomy prior to the start of follow-up. Genetic ancestry groupings are according to iadmix, derived as described in the Methods: EUR, European; AFR, African; EAS, East Asian; SAS, South Asian. [a]SNP variant name. ***Subtable S8a***: EAFs on UK Biobank women.

UKB rs number	SNP[a]	EAFs on UK Biobank women				
		EUR	*AFR*	*EAS*	*SAS*	*Mixed*
rs707475	1_7917076_G_A	0.393	0.506	0.223	0.26	0.359
rs616488	1_10566215_A_G	0.345	0.088	0.335	0.235	0.263
rs2992756	1_18807339_T_C	0.501	0.465	0.851	0.448	0.51
rs4233486	1_41380440_C_T	0.657	0.765	0.654	0.645	0.672
rs114282204	1_41389220_T_C	0.018	0.005	0.003	0.015	0.015
1.46670206_TC_T	1_46670206_TC_T	0.302	0.081	0.017	0.098	0.167
1.51467096_CT_C	1_51467096_CT_C	0.492	0.532	0.327	0.453	0.492
rs17426269	1_88156923_G_A	0.153	0.104	0.009	0.121	0.129
rs2151842	1_88428199_C_A	0.238	0.161	0.118	0.254	0.199
rs612683	1_100880328_A_T	0.402	0.236	0.536	0.453	0.394
1.110198129_CAAA_C	1_110198129_CAAA_C	0.788	0.403	0.936	0.786	0.721
rs7513707	1_114445880_G_A	0.174	0.016	0.579	0.144	0.175
rs12406858	1_118141492_A_C	0.255	0.353	0.416	0.345	0.334
rs637868	1_120257110_T_C	0.528	0.54	0.919	0.712	0.61
rs11249433	1_121280613_A_G	0.415	0.076	0.026	0.217	0.278
rs111458676	1_121287994_A_G	0.093	0.032	0.171	0.3	0.153
rs143384623	1_145604302_C_CT	0.356	0.092	0.174	0.271	0.259
rs11205303	1_149906413_T_C	0.407	0.033	0.34	0.324	0.304
rs12091730	1_155556971_G_A	0.231	0.044	0.666	0.276	0.232
rs761575824	1_168171052_CA_C	0.101	0.244	0.004	0.041	0.109
rs11463354	1_172328767_T_TA	0.322	0.496	0.137	0.274	0.321
rs35383942	1_201437832_C_T	0.062	0.003	0	0.005	0.028
rs6686987	1_202184600_C_T	0.402	0.261	0.253	0.289	0.306
rs7514172	1_203770448_T_A	0.275	0.176	0.354	0.295	0.268
rs11268668	1_204502514_T_TTCTGAAACAGGG	0.783	0.978	0.987	0.925	0.871
rs2785646	1_208076291_G_A	0.349	0.087	0.023	0.158	0.216
rs2576261	1_217053815_T_G	0.329	0.839	0.156	0.29	0.416
rs11117758	1_217220574_G_A	0.212	0.136	0.045	0.262	0.211
rs11118563	1_220671050_C_T	0.233	0.022	0.306	0.186	0.201
rs72755295	1_242034263_A_G	0.034	0.002	0.001	0.025	0.019
rs78425380	2_10138983_T_C	0.11	0.094	0.005	0.077	0.09
rs6743383	2_19315675_T_A	0.558	0.448	0.604	0.371	0.478
rs6725517	2_25129473_A_G	0.431	0.814	0.406	0.405	0.476
rs12472404	2_29179452_G_C	0.232	0.168	0.781	0.3	0.287
rs4322799	2_29615233_T_C	0.237	0.285	0.035	0.297	0.255
rs553796823	2_39699510_C_CT	0.462	0.22	0.818	0.607	0.482
rs6756513	2_70172587_G_A	0.282	0.264	0.277	0.275	0.239
rs1036759	2_88358825_G_C	0.305	0.483	0.27	0.282	0.346
rs6746250	2_121058254_A_G	0.691	0.821	0.424	0.608	0.693
rs17625845	2_121089731_T_C	0.196	0.046	0.082	0.11	0.148
rs10164550	2_121159205_G_A	0.356	0.666	0.142	0.393	0.415
rs10179592	2_121246568_T_C	0.9	0.684	0.773	0.789	0.818
rs17726078	2_172974566_C_G	0.48	0.231	0.212	0.365	0.375
rs1550622	2_174212910_A_G	0.832	0.744	0.991	0.88	0.842
rs2356656	2_192381934_C_T	0.873	0.678	0.936	0.936	0.859
rs10197246	2_202204741_T_C	0.732	0.788	0.695	0.833	0.732
rs4442975	2_217920769_G_T	0.512	0.301	0.881	0.472	0.475
2.217955896_GA_G	2_217955896_GA_G	0.036	0.15	0	0.027	0.05
rs11693806	2_218292158_C_G	0.735	0.493	0.39	0.389	0.578
rs3791977	2_218714845_G_A	0.399	0.195	0.26	0.321	0.301
rs4676356	2_241388857_C_A	0.973	0.999	0.999	0.987	0.986
rs6762558	3_4742251_A_G	0.407	0.259	0.089	0.112	0.249
rs552647	3_27353716_C_A	0.525	0.963	0.266	0.513	0.617
rs62255657	3_27388664_C_G	0.255	0.264	0.147	0.318	0.302
rs112476261	3_29294845_C_T	0.015	0.001	0	0.008	0.009
rs17838698	3_30684907_C_T	0.286	0.298	0.701	0.355	0.324
rs56387622	3_46888198_T_C	0.1	0.337	0.139	0.235	0.188
rs371314787	3_49709912_C_CT	0.29	0.239	0.048	0.23	0.245
rs138866686	3_55970777_A_AT	0.025	0.002	0.002	0.007	0.019
rs2886671	3_59373745_C_T	0.42	0.506	0.685	0.469	0.461
rs73130576	3_63887449_T_TTG	0.138	0.021	0.134	0.13	0.121
rs9825432	3_71620370_T_G	0.639	0.593	0.038	0.449	0.52
rs13066793	3_87037543_A_G	0.09	0.036	0.006	0.077	0.063
rs639355	3_99403877_G_A	0.48	0.262	0.48	0.366	0.407
3.141112859_CTT_C	3_141112859_CTT_C	0.428	0.85	0.064	0.203	0.411
rs58058861	3_172285237_G_A	0.22	0.141	0.312	0.237	0.214
rs9882792	3_189774456_C_T	0.221	0.131	0.067	0.193	0.168
rs10012017	4_38784633_G_T	0.204	0.501	0.438	0.411	0.382
rs17014016	4_89240476_G_A	0.444	0.443	0.012	0.273	0.358
rs775780062	4_92594859_TTCTTTC_T	0.452	0.46	0.584	0.337	0.432
rs62331150	4_106069013_G_T	0.205	0.016	0.629	0.294	0.235
rs56039025	4_143467195_C_T	0.113	0.151	0.048	0.121	0.123
rs745707748	4_151218296_CATATTT_C	0.64	0.422	0.238	0.443	0.51
rs28436676	4_175842495_G_A	0.119	0.454	0.251	0.19	0.219
rs62334414	4_175847436_C_A	0.341	0.153	0.026	0.297	0.246
4.187513623_TA_T	4_187503758_A_T	0.461	0.33	0.391	0.34	0.419
rs62641919	5_345109_T_C	0.06	0.626	0.005	0.06	0.167
rs10069690	5_1279790_C_T	0.257	0.646	0.184	0.258	0.332
rs3215401	5_1296255_A_AG	0.315	0.092	0.349	0.56	0.338
rs62329727	5_1353077_T_C	0.012	0.001	0.002	0.002	0.005
rs4866496	5_2777029_G_A	0.42	0.534	0.789	0.469	0.485
rs17611291	5_16231194_G_C	0.553	0.174	0.162	0.41	0.413
rs770436441	5_32579616_TCA_T	0.476	0.138	0.118	0.368	0.374
rs138335056	5_44508264_G_GT	0.116	0.249	0.007	0.119	0.143
rs187108781	5_44619502_A_G	0.158	0.009	0.001	0.046	0.091
rs4613718	5_44649944_C_T	0.607	0.757	0.614	0.677	0.654
rs10941679	5_44706498_A_G	0.252	0.179	0.535	0.358	0.284
rs17343002	5_44853593_G_C	0.313	0.17	0.054	0.128	0.223
rs10940312	5_52679539_C_CA	0.103	0.02	0.197	0.118	0.097
rs553874618	5_55662540_C_CT	0.36	0.265	0.371	0.37	0.361
rs889310	5_55965167_C_T	0.561	0.605	0.551	0.682	0.619
rs16886165	5_56023083_T_G	0.161	0.342	0.369	0.334	0.245
rs76250845	5_56042972_C_T	0.059	0.058	0.111	0.033	0.054
rs11949391	5_56045081_T_C	0.167	0.084	0.045	0.186	0.147
rs113778879	5_58241712_C_T	0.573	0.572	0.678	0.595	0.589
rs3010266	5_71965007_G_A	0.261	0.214	0.123	0.323	0.243
rs157557	5_73234583_T_C	0.33	0.248	0.424	0.404	0.336
rs767431357	5_77155397_GT_G	0.346	0.031	0.193	0.264	0.246
rs34525310	5_79180995_G_GA	0.175	0.109	0.378	0.248	0.167
5.81512947_TA_T	5_81512947_TA_T	0.251	0.097	0.001	0.106	0.171
rs332529	5_90789470_G_A	0.152	0.311	0.492	0.379	0.258
rs17157372	5_104300273_G_T	0.17	0.417	0.062	0.179	0.219
rs335160	5_122478676_C_A	0.751	0.695	0.606	0.62	0.701
rs1428387	5_122705244_C_T	0.023	0.06	0.091	0.111	0.058
rs6860806	5_131640536_A_G	0.55	0.37	0.263	0.203	0.403
rs6596100	5_132407058_C_T	0.236	0.282	0.12	0.254	0.238
rs1432679	5_158244083_C_T	0.553	0.168	0.395	0.668	0.48
rs10074269	5_169591460_T_C	0.343	0.312	0.485	0.439	0.376
rs6864691	5_173358154_G_A	0.422	0.446	0.325	0.532	0.441
rs4868701	5_176134882_T_C	0.541	0.745	0.508	0.556	0.58
rs418053	6_13713366_G_C	0.575	0.594	0.398	0.56	0.567
rs3819405	6_16399557_C_T	0.33	0.473	0.36	0.487	0.399
rs12211970	6_18783140_G_A	0.61	0.476	0.714	0.7	0.594
rs769485514	6_20537845_CA_C	0.493	0.754	0.346	0.364	0.498
rs9358466	6_21923810_T_C	0.433	0.285	0.317	0.398	0.401
rs34196306	6_27425644_G_C	0.107	0.006	0	0	0.032
rs111342015	6_43227141_G_A	0.099	0.007	0.004	0.096	0.065
rs10623112	6_82263549_AAT_A	0.416	0.281	0.384	0.412	0.401
6.85912194_CAA_C	6_85912194_CAA_C	0.065	0.005	0.002	0.016	0.032
rs73754909	6_87803819_T_C	0.277	0.729	0.253	0.279	0.369
rs55941023	6_130341728_C_CT	0.699	0.742	0.91	0.67	0.732
rs2121348	6_149595505_T_C	0.201	0.181	0.435	0.216	0.218
rs6913578	6_151949806_A_C	0.322	0.5	0.331	0.345	0.352
rs60954078	6_151955914_A_G	0.072	0.388	0.295	0.069	0.144
6.152022664_CAAAAAAA_C	6_152022664_CAAAAAAA_C	0.614	0.716	0.399	0.52	0.585
rs851984	6_152023191_G_A	0.391	0.223	0.112	0.236	0.302
rs6904031	6_152055978_A_T	0.057	0.173	0.069	0.049	0.085
rs910416	6_152432902_C_T	0.509	0.527	0.547	0.496	0.495
rs9364472	6_169006947_C_G	0.511	0.474	0.408	0.607	0.562
rs6940159	6_170332621_T_C	0.621	0.573	0.171	0.345	0.504
rs7971	7_21940960_A_G	0.36	0.086	0.191	0.266	0.264
rs289997	7_25569548_C_T	0.147	0.039	0.237	0.154	0.142
rs74765302	7_28869017_G_A	0.11	0.195	0.002	0.084	0.113
rs13244925	7_55192256_A_C	0.565	0.318	0.7	0.499	0.495
rs17268829	7_94113799_T_C	0.298	0.05	0.258	0.368	0.267
rs4439053	7_98005235_G_A	0.171	0.056	0.151	0.107	0.121
rs111963714	7_99948655_T_G	0.213	0.186	0.038	0.135	0.178
rs71559437	7_101552440_G_A	0.135	0.039	0.079	0.084	0.095
rs7800548	7_102481842_T_C	0.339	0.753	0.61	0.481	0.474
rs12706954	7_130656911_C_T	0.385	0.449	0.29	0.406	0.395
rs68056147	7_130674481_G_A	0.305	0.096	0.269	0.346	0.256
rs201664599	7_139943702_CT_C	0.543	0.481	0.542	0.583	0.562
rs62485509	7_144048902_G_T	0.234	0.015	0.043	0.161	0.174
rs66823261	8_170692_T_C	0.219	0.292	0.199	0.243	0.238
8.17787610_CT_C	8_17787610_CT_C	0.633	0.403	0.347	0.498	0.531
rs1028016	8_23447496_A_G	0.626	0.84	0.817	0.572	0.689
rs310295	8_23663653_C_A	0.419	0.477	0.328	0.446	0.433
rs9693444	8_29509616_A_C	0.676	0.617	0.727	0.796	0.683
rs13365225	8_36858483_A_G	0.165	0.441	0.351	0.308	0.278
rs1511243	8_76230943_A_G	0.823	0.913	0.968	0.907	0.884
rs72658084	8_76333056_C_T	0.092	0.061	0.007	0.046	0.076
rs1533366	8_76378165_G_T	0.361	0.135	0.238	0.25	0.284
rs62517052	8_102483100_T_C	0.097	0.272	0.004	0.08	0.117
rs12546444	8_106358620_A_T	0.099	0.025	0.121	0.111	0.092
rs13267382	8_117209548_A_G	0.653	0.049	0.461	0.389	0.436
rs62526620	8_120862186_A_G	0.132	0.077	0.034	0.133	0.126
rs35542655	8_124563705_T_C	0.146	0.332	0.178	0.263	0.196
rs12541094	8_124571581_G_A	0.416	0.424	0.387	0.527	0.428
rs7842619	8_124739913_T_G	0.413	0.274	0.258	0.487	0.378
rs35961416	8_128213561_C_CA	0.416	0.399	0.134	0.202	0.334
rs12550713	8_128370949_C_G	0.401	0.53	0.534	0.504	0.477
rs10096351	8_128372172_A_G	0.544	0.625	0.749	0.696	0.63
rs1016578	8_129199566_G_A	0.179	0.025	0.201	0.236	0.179
rs7830152	8_143669254_A_G	0.338	0.7	0.806	0.522	0.478
rs10975870	9_6880263_A_G	0.293	0.067	0.004	0.076	0.18
rs745322029	9_21964882_CAAAA_C	0.336	0.52	0.228	0.288	0.376
rs17694493	9_22041998_C_G	0.135	0.109	0.03	0.069	0.109
rs4880038	9_36928288_T_C	0.557	0.518	0.74	0.506	0.547
rs665889	9_87782211_T_C	0.509	0.422	0.793	0.596	0.544
rs10120432	9_98362587_T_C	0.093	0.318	0.31	0.186	0.175
9.110303808_TAA_T	9_110303808_TAA_T	0.185	0.16	0.435	0.256	0.253
rs10816625	9_110837073_A_G	0.063	0.006	0.4	0.074	0.086
rs13294895	9_110837176_C_T	0.178	0.018	0.023	0.122	0.108
rs7848334	9_110849525_G_T	0.601	0.942	0.957	0.829	0.769
rs630965	9_110885479_C_T	0.617	0.491	0.927	0.839	0.675
rs1895062	9_119313486_A_G	0.408	0.121	0.303	0.28	0.319
rs3861871	9_129424719_A_G	0.453	0.485	0.565	0.499	0.469
rs550057	9_136146597_C_T	0.255	0.145	0.201	0.174	0.219
rs55910451	10_5794652_A_G	0.222	0.052	0.156	0.119	0.166
rs10796139	10_13892298_G_A	0.448	0.518	0.636	0.524	0.497
rs7072776	10_22032942_A_G	0.722	0.469	0.931	0.793	0.666
rs762131501	10_22477776_ACC_A	0.017	0.465	0.007	0.034	0.118
rs10764337	10_22861490_A_C	0.944	0.99	0.999	0.96	0.959
rs2384736	10_38523626_C_A	0.385	0.401	0.566	0.266	0.391
rs10995201	10_64299890_A_G	0.149	0.196	0.021	0.088	0.13
rs6479868	10_64819996_G_T	0.196	0.306	0.171	0.143	0.204
rs111833376	10_71335574_C_T	0.318	0.363	0.094	0.237	0.27
rs719338	10_80851257_G_T	0.597	0.768	0.622	0.709	0.655
rs4980029	10_80886726_A_G	0.158	0.428	0.413	0.372	0.307
10.95292187_CAA_C	10_95292187_CAA_C	0.811	0.704	0.641	0.753	0.769
rs10885405	10_114777670_C_T	0.465	0.804	0.038	0.394	0.495
rs12250948	10_115128491_T_C	0.789	0.227	0.232	0.557	0.581
rs9421410	10_123095209_G_A	0.317	0.537	0.398	0.28	0.387
rs45631580	10_123340107_A_G	0.06	0.246	0.095	0.116	0.124
10.123340431_GC_G	10_123340431_GC_G	0.596	0.357	0.54	0.594	0.534
rs45631563	10_123349324_A_T	0.045	0.017	0.023	0.09	0.056
rs7394715	11_433617_T_C	0.795	0.986	0.997	0.962	0.901
rs6597981	11_803017_A_G	0.518	0.119	0.248	0.221	0.355
rs4980386	11_1895708_C_A	0.38	0.16	0.694	0.397	0.385
rs10832963	11_18664241_T_G	0.745	0.105	0.494	0.604	0.573
rs4472923	11_42844441_C_T	0.33	0.401	0.24	0.268	0.309
rs10838267	11_44368892_G_A	0.545	0.145	0.332	0.426	0.414
rs77047825	11_46318032_C_G	0.074	0.004	0.001	0.008	0.038
rs12287832	11_65553492_C_A	0.191	0.043	0.125	0.052	0.122
rs10896047	11_65572431_G_A	0.474	0.157	0.205	0.555	0.409
rs35039974	11_69328130_A_T	0.204	0.042	0.232	0.209	0.183
rs661204	11_69330983_G_A	0.12	0.147	0.023	0.157	0.125
rs78540526	11_69331418_C_T	0.07	0.015	0.009	0.106	0.06
rs7125780	11_103614438_T_G	0.644	0.345	0.659	0.634	0.584
rs199504893	11_108267402_C_CA	0.41	0.136	0.399	0.526	0.419
rs610437	11_111696440_T_C	0.621	0.617	0.766	0.639	0.652
rs625145	11_116727936_A_T	0.19	0.649	0.169	0.287	0.296
rs7121616	11_122966626_A_G	0.281	0.703	0.347	0.353	0.395
rs7939702	11_129243417_T_G	0.857	0.832	0.965	0.951	0.881
rs11822830	11_129461016_A_G	0.585	0.834	0.54	0.677	0.646
rs797736	12_293626_A_G	0.368	0.431	0.443	0.475	0.395
rs12422552	12_14413931_G_C	0.267	0.433	0.28	0.356	0.313
rs788458	12_28149568_C_T	0.124	0.015	0.187	0.151	0.103
rs7297051	12_28174817_C_T	0.234	0.118	0.241	0.254	0.226
rs11049431	12_28347382_C_T	0.224	0.014	0.009	0.135	0.125
rs1027113	12_29140260_G_A	0.922	0.979	0.778	0.919	0.926
rs2277339	12_57146069_T_G	0.104	0.222	0.183	0.094	0.138
rs2870876	12_70798355_A_T	0.187	0.067	0.339	0.239	0.176
rs111622698	12_83064195_G_GA	0.106	0.021	0.161	0.103	0.097
rs10862899	12_85004551_C_T	0.511	0.134	0.126	0.396	0.388
rs17356907	12_96027759_A_G	0.305	0.182	0.261	0.281	0.259
rs7132703	12_103097887_C_T	0.117	0.304	0.003	0.066	0.138
rs11065822	12_111600134_G_T	0.355	0.022	0.006	0.084	0.189
rs1061657	12_115108136_T_C	0.258	0.051	0.451	0.203	0.227
rs11067551	12_115796577_A_G	0.196	0.433	0.295	0.327	0.298
rs2454399	12_115835836_T_C	0.424	0.463	0.278	0.576	0.464
rs206966	12_120832146_C_T	0.155	0.171	0.076	0.163	0.152
rs56404467	13_32839990_G_A	0.021	0.004	0.01	0.022	0.017
rs11571833	13_32972626_A_T	0.009	0.001	0.001	0.007	0.007
rs9315973	13_43501356_A_G	0.83	0.719	0.613	0.703	0.775
rs12870942	13_73806982_T_C	0.32	0.023	0.257	0.183	0.208
rs2181965	13_73960952_A_G	0.757	0.657	0.993	0.911	0.785
rs34914085	14_37128564_C_A	0.212	0.023	0.287	0.164	0.161
rs2253012	14_37228504_C_T	0.44	0.509	0.11	0.364	0.411
rs2588809	14_68660428_T_C	0.842	0.677	0.98	0.835	0.788
rs11624333	14_68979835_T_C	0.284	0.02	0.049	0.165	0.16
14.91751788_TC_T	14_91751788_TC_T	0.685	0.595	0.496	0.608	0.629
rs941764	14_91841069_A_G	0.341	0.781	0.128	0.294	0.421
rs78440108	14_93070286_C_T	0.179	0.104	0.008	0.131	0.136
rs4983544	14_105213978_T_G	0.447	0.903	0.651	0.582	0.594
rs187010898	15_46680811_C_A	0.012	0.018	0.001	0.003	0.009
rs4774565	15_50694306_A_G	0.33	0.526	0.508	0.261	0.361
rs8042593	15_66630569_G_A	0.646	0.528	0.799	0.602	0.612
rs35874463	15_67457698_A_G	0.057	0.003	0.001	0.04	0.039
rs8035987	15_75750383_T_C	0.257	0.661	0.362	0.411	0.422
rs2290202	15_91512267_G_T	0.13	0.15	0.506	0.209	0.179
rs144767203	15_100905819_A_C	0.108	0.256	0.226	0.148	0.176
16.4008542_CAAAAA_C	16_4008542_CAAAAA_C	0.823	0.861	0.933	0.868	0.84
rs11076805	16_4106788_C_A	0.266	0.19	0.106	0.253	0.22
rs12709163	16_6963972_C_G	0.805	0.628	0.911	0.808	0.77
rs34872983	16_10706580_G_A	0.063	0.017	0.267	0.134	0.083
rs75753503	16_23007047_G_T	0.023	0.045	0.024	0.009	0.027
rs35668161	16_52538825_C_A	0.25	0.051	0.182	0.19	0.187
rs4784227	16_52599188_C_T	0.238	0.039	0.238	0.203	0.194
rs55872725	16_53809123_C_T	0.403	0.059	0.152	0.339	0.302
rs6499648	16_53861139_C_T	0.772	0.837	0.683	0.767	0.771
rs7184573	16_53861592_G_A	0.377	0.203	0.199	0.352	0.346
rs28539243	16_54682064_G_A	0.488	0.877	0.58	0.637	0.618
rs7500067	16_80648296_A_G	0.223	0.229	0.249	0.128	0.23
rs9931038	16_85145977_T_C	0.488	0.6	0.147	0.504	0.49
rs12449271	16_87086492_T_C	0.256	0.03	0.171	0.164	0.179
rs79461387	17_29168077_G_T	0.265	0.034	0.103	0.12	0.161
rs150537328	17_39251123_T_C	0.068	0.005	0.003	0.043	0.042
rs11296	17_40127060_T_C	0.057	0.008	0.002	0.01	0.032
rs17881320	17_40485239_G_T	0.09	0.014	0.009	0.054	0.051
rs149370081	17_40744470_G_A	0.015	0.001	0	0.003	0.007
rs545502941	17_43212339_C_CT	0.219	0.235	0.127	0.173	0.216
rs2668667	17_44283858_G_A	0.171	0.011	0.002	0.059	0.089
rs2787486	17_53209774_A_C	0.315	0.438	0.309	0.211	0.311
rs745570	17_77781725_A_G	0.515	0.823	0.417	0.58	0.591
rs16976596	18_11696613_C_T	0.132	0.754	0.03	0.113	0.256
rs11665269	18_20634253_C_T	0.629	0.087	0.469	0.556	0.492
rs1111207	18_24125857_T_C	0.43	0.357	0.654	0.411	0.429
rs527616	18_24337424_C_G	0.641	0.878	0.704	0.713	0.718
18.24518050_AT_A	18_24518050_AT_A	0.288	0.173	0.479	0.199	0.261
rs8092192	18_25407513_C_G	0.703	0.389	0.462	0.565	0.608
rs72931898	18_29981526_G_A	0.045	0.177	0.015	0.044	0.062
rs9954058	18_42411803_G_C	0.079	0.344	0.133	0.103	0.147
rs9952980	18_42888797_T_C	0.358	0.914	0.29	0.381	0.467
rs117922601	19_13249921_G_T	0.056	0.006	0	0.007	0.021
rs56069439	19_17393925_C_A	0.294	0.227	0.004	0.159	0.208
rs10164323	19_18569492_C_T	0.354	0.808	0.216	0.426	0.436
rs140702307	19_19517054_C_CGGGCG	0.355	0.839	0.377	0.615	0.522
rs56681946	19_44283031_T_C	0.375	0.221	0.132	0.437	0.322
rs4399645	19_46166073_T_C	0.592	0.64	0.401	0.689	0.629
rs1172821	19_55816678_C_T	0.372	0.438	0.101	0.489	0.39
rs16991615	20_5948227_G_A	0.065	0.003	0	0.026	0.038
rs1154723	20_11379842_T_C	0.949	0.998	0.999	0.991	0.979
rs6030585	20_41613706_C_G	0.795	0.981	0.847	0.908	0.87
rs13039563	20_52296849_G_A	0.242	0.054	0.324	0.317	0.236
rs2822999	21_16364756_T_G	0.18	0.019	0.15	0.143	0.13
rs2823130	21_16566350_A_G	0.087	0.219	0.1	0.099	0.105
rs2403907	21_16574455_C_A	0.316	0.436	0.109	0.334	0.355
rs4818836	21_47762932_G_A	0.032	0.002	0.015	0.032	0.029
rs9798754	22_19766137_C_T	0.369	0.465	0.554	0.588	0.468
rs17879961	22_29121087_A_G	0.001	0	0	0	0.001
rs5997390	22_29135543_G_A	0.08	0.066	0.08	0.102	0.07
rs34134147	22_29203724_C_T	0.022	0.002	0	0.01	0.013
rs132289	22_29551872_A_G	0.978	0.998	1	0.996	0.991
rs5750715	22_39343916_T_A	0.251	0.169	0.503	0.364	0.264
22.40904707_CT_C	22_40904707_CT_C	0.094	0.131	0.213	0.122	0.117
rs9611990	22_43433100_C_T	0.121	0.011	0.002	0.078	0.078
rs112855987	22_45319953_G_A	0.419	0.254	0.245	0.285	0.332
rs28512361	22_46283297_G_A	0.107	0.302	0.037	0.091	0.126

Table S8. Allele frequencies (EAFs) by genetic ancestry for SNPs included in the PGS models. EAFs were estimated in UK Biobank individuals without a diagnosis of cancer or mastectomy prior to the start of follow-up. Genetic ancestry groupings are according to iadmix, derived as described in the Methods: EUR, European; AFR, African; EAS, East Asian; SAS, South Asian. [a]SNP variant name. ***Subtable S8b***: EAFs on UK Biobank men.

UKB rs number	SNP[a]	EAFs on UK Biobank men				
		EUR	*AFR*	*EAS*	*SAS*	*Mixed*
rs707475	1_7917076_G_A	0.392	0.514	0.199	0.265	0.368
rs616488	1_10566215_A_G	0.345	0.09	0.325	0.22	0.247
rs2992756	1_18807339_T_C	0.5	0.458	0.858	0.442	0.511
rs4233486	1_41380440_C_T	0.659	0.755	0.689	0.652	0.65
rs114282204	1_41389220_T_C	0.018	0.004	0.002	0.017	0.017
1.46670206_TC_T	1_46670206_TC_T	0.303	0.075	0.019	0.098	0.177
1.51467096_CT_C	1_51467096_CT_C	0.491	0.538	0.298	0.438	0.513
rs17426269	1_88156923_G_A	0.154	0.097	0.007	0.118	0.139
rs2151842	1_88428199_C_A	0.24	0.151	0.1	0.251	0.205
rs612683	1_100880328_A_T	0.403	0.234	0.55	0.462	0.396
1.110198129_CAAA_C	1_110198129_CAAA_C	0.787	0.396	0.933	0.794	0.731
rs7513707	1_114445880_G_A	0.174	0.015	0.6	0.14	0.17
rs12406858	1_118141492_A_C	0.256	0.35	0.411	0.353	0.333
rs637868	1_120257110_T_C	0.528	0.541	0.92	0.711	0.606
rs11249433	1_121280613_A_G	0.417	0.065	0.032	0.205	0.311
rs111458676	1_121287994_A_G	0.093	0.035	0.17	0.295	0.177
rs143384623	1_145604302_C_CT	0.355	0.094	0.172	0.275	0.245
rs11205303	1_149906413_T_C	0.406	0.025	0.316	0.341	0.304
rs12091730	1_155556971_G_A	0.23	0.042	0.658	0.263	0.219
rs761575824	1_168171052_CA_C	0.102	0.239	0.004	0.037	0.114
rs11463354	1_172328767_T_TA	0.323	0.5	0.128	0.273	0.331
rs35383942	1_201437832_C_T	0.063	0.003	0.001	0.007	0.028
rs6686987	1_202184600_C_T	0.404	0.26	0.229	0.285	0.312
rs7514172	1_203770448_T_A	0.276	0.183	0.349	0.305	0.259
rs11268668	1_204502514_T_TTCTGAAACAGGG	0.782	0.979	0.986	0.927	0.864
rs2785646	1_208076291_G_A	0.351	0.091	0.019	0.155	0.207
rs2576261	1_217053815_T_G	0.328	0.859	0.174	0.286	0.405
rs11117758	1_217220574_G_A	0.211	0.149	0.051	0.26	0.213
rs11118563	1_220671050_C_T	0.234	0.013	0.329	0.186	0.217
rs72755295	1_242034263_A_G	0.033	0.001	0.001	0.022	0.019
rs78425380	2_10138983_T_C	0.109	0.095	0.008	0.07	0.09
rs6743383	2_19315675_T_A	0.558	0.454	0.583	0.355	0.464
rs6725517	2_25129473_A_G	0.432	0.821	0.387	0.404	0.462
rs12472404	2_29179452_G_C	0.23	0.165	0.793	0.297	0.275
rs4322799	2_29615233_T_C	0.238	0.296	0.033	0.294	0.264
rs553796823	2_39699510_C_CT	0.463	0.232	0.828	0.599	0.502
rs6756513	2_70172587_G_A	0.28	0.271	0.276	0.278	0.222
rs1036759	2_88358825_G_C	0.306	0.469	0.276	0.279	0.337
rs6746250	2_121058254_A_G	0.69	0.828	0.399	0.607	0.693
rs17625845	2_121089731_T_C	0.194	0.043	0.082	0.109	0.148
rs10164550	2_121159205_G_A	0.358	0.685	0.131	0.391	0.408
rs10179592	2_121246568_T_C	0.899	0.674	0.748	0.79	0.825
rs17726078	2_172974566_C_G	0.478	0.224	0.213	0.36	0.355
rs1550622	2_174212910_A_G	0.832	0.749	0.99	0.879	0.846
rs2356656	2_192381934_C_T	0.873	0.676	0.937	0.941	0.872
rs10197246	2_202204741_T_C	0.731	0.799	0.703	0.833	0.727
rs4442975	2_217920769_G_T	0.511	0.307	0.902	0.467	0.463
2.217955896_GA_G	2_217955896_GA_G	0.036	0.158	0	0.032	0.044
rs11693806	2_218292158_C_G	0.735	0.499	0.344	0.406	0.569
rs3791977	2_218714845_G_A	0.399	0.202	0.26	0.323	0.3
rs4676356	2_241388857_C_A	0.973	0.999	0.998	0.986	0.984
rs6762558	3_4742251_A_G	0.409	0.263	0.091	0.119	0.231
rs552647	3_27353716_C_A	0.526	0.972	0.256	0.502	0.618
rs62255657	3_27388664_C_G	0.257	0.265	0.149	0.303	0.33
rs112476261	3_29294845_C_T	0.016	0.001	0.001	0.009	0.01
rs17838698	3_30684907_C_T	0.287	0.299	0.662	0.353	0.338
rs56387622	3_46888198_T_C	0.1	0.329	0.157	0.227	0.185
rs371314787	3_49709912_C_CT	0.287	0.243	0.055	0.234	0.259
rs138866686	3_55970777_A_AT	0.025	0.002	0.003	0.007	0.02
rs2886671	3_59373745_C_T	0.421	0.509	0.689	0.458	0.456
rs73130576	3_63887449_T_TTG	0.138	0.019	0.156	0.133	0.123
rs9825432	3_71620370_T_G	0.637	0.597	0.039	0.435	0.534
rs13066793	3_87037543_A_G	0.09	0.031	0.007	0.073	0.063
rs639355	3_99403877_G_A	0.481	0.255	0.49	0.372	0.408
3.141112859_CTT_C	3_141112859_CTT_C	0.428	0.852	0.073	0.202	0.377
rs58058861	3_172285237_G_A	0.22	0.141	0.332	0.238	0.208
rs9882792	3_189774456_C_T	0.222	0.128	0.092	0.198	0.172
rs10012017	4_38784633_G_T	0.204	0.51	0.442	0.411	0.408
rs17014016	4_89240476_G_A	0.443	0.443	0.018	0.268	0.352
rs775780062	4_92594859_TTCTTTC_T	0.454	0.458	0.602	0.342	0.433
rs62331150	4_106069013_G_T	0.205	0.013	0.629	0.299	0.24
rs56039025	4_143467195_C_T	0.112	0.156	0.038	0.116	0.113
rs745707748	4_151218296_CATATTT_C	0.64	0.429	0.225	0.454	0.51
rs28436676	4_175842495_G_A	0.119	0.466	0.276	0.191	0.2
rs62334414	4_175847436_C_A	0.341	0.141	0.028	0.294	0.25
4.187513623_TA_T	4_187503758_A_T	0.461	0.344	0.389	0.331	0.431
rs62641919	5_345109_T_C	0.059	0.64	0.007	0.061	0.15
rs10069690	5_1279790_C_T	0.259	0.66	0.172	0.252	0.306
rs3215401	5_1296255_A_AG	0.314	0.099	0.36	0.549	0.361
rs62329727	5_1353077_T_C	0.012	0.001	0.004	0.002	0.004
rs4866496	5_2777029_G_A	0.422	0.53	0.791	0.465	0.471
rs17611291	5_16231194_G_C	0.552	0.174	0.169	0.417	0.419
rs770436441	5_32579616_TCA_T	0.475	0.125	0.109	0.368	0.393
rs138335056	5_44508264_G_GT	0.115	0.253	0.006	0.112	0.156
rs187108781	5_44619502_A_G	0.157	0.007	0.003	0.047	0.087
rs4613718	5_44649944_C_T	0.605	0.749	0.579	0.666	0.656
rs10941679	5_44706498_A_G	0.252	0.176	0.512	0.351	0.28
rs17343002	5_44853593_G_C	0.314	0.165	0.053	0.128	0.221
rs10940312	5_52679539_C_CA	0.103	0.02	0.207	0.112	0.104
rs553874618	5_55662540_C_CT	0.361	0.265	0.375	0.37	0.381
rs889310	5_55965167_C_T	0.561	0.618	0.582	0.684	0.618
rs16886165	5_56023083_T_G	0.162	0.349	0.366	0.326	0.233
rs76250845	5_56042972_C_T	0.06	0.054	0.11	0.032	0.046
rs11949391	5_56045081_T_C	0.165	0.084	0.043	0.175	0.143
rs113778879	5_58241712_C_T	0.574	0.57	0.68	0.609	0.586
rs3010266	5_71965007_G_A	0.262	0.203	0.106	0.319	0.231
rs157557	5_73234583_T_C	0.329	0.255	0.426	0.413	0.343
rs767431357	5_77155397_GT_G	0.346	0.026	0.193	0.27	0.263
rs34525310	5_79180995_G_GA	0.175	0.106	0.371	0.243	0.165
5.81512947_TA_T	5_81512947_TA_T	0.25	0.101	0.003	0.1	0.183
rs332529	5_90789470_G_A	0.152	0.312	0.483	0.388	0.255
rs17157372	5_104300273_G_T	0.17	0.411	0.048	0.184	0.216
rs335160	5_122478676_C_A	0.75	0.703	0.618	0.611	0.707
rs1428387	5_122705244_C_T	0.024	0.06	0.088	0.105	0.051
rs6860806	5_131640536_A_G	0.551	0.374	0.265	0.209	0.402
rs6596100	5_132407058_C_T	0.236	0.284	0.13	0.244	0.244
rs1432679	5_158244083_C_T	0.554	0.167	0.377	0.674	0.51
rs10074269	5_169591460_T_C	0.345	0.313	0.471	0.435	0.389
rs6864691	5_173358154_G_A	0.422	0.45	0.298	0.526	0.461
rs4868701	5_176134882_T_C	0.54	0.748	0.512	0.56	0.584
rs418053	6_13713366_G_C	0.574	0.587	0.404	0.551	0.568
rs3819405	6_16399557_C_T	0.331	0.474	0.362	0.489	0.401
rs12211970	6_18783140_G_A	0.611	0.465	0.713	0.703	0.612
rs769485514	6_20537845_CA_C	0.491	0.757	0.37	0.368	0.495
rs9358466	6_21923810_T_C	0.433	0.282	0.322	0.381	0.414
rs34196306	6_27425644_G_C	0.107	0.006	0	0	0.024
rs111342015	6_43227141_G_A	0.099	0.006	0.006	0.085	0.066
rs10623112	6_82263549_AAT_A	0.416	0.281	0.393	0.42	0.41
6.85912194_CAA_C	6_85912194_CAA_C	0.065	0.004	0.001	0.014	0.033
rs73754909	6_87803819_T_C	0.276	0.732	0.268	0.275	0.363
rs55941023	6_130341728_C_CT	0.699	0.742	0.914	0.679	0.752
rs2121348	6_149595505_T_C	0.202	0.177	0.42	0.205	0.23
rs6913578	6_151949806_A_C	0.324	0.51	0.354	0.348	0.372
rs60954078	6_151955914_A_G	0.072	0.378	0.319	0.066	0.151
6.152022664_CAAAAAAA_C	6_152022664_CAAAAAAA_C	0.615	0.721	0.428	0.515	0.576
rs851984	6_152023191_G_A	0.391	0.229	0.142	0.222	0.297
rs6904031	6_152055978_A_T	0.057	0.181	0.087	0.046	0.074
rs910416	6_152432902_C_T	0.508	0.528	0.562	0.49	0.467
rs9364472	6_169006947_C_G	0.509	0.473	0.413	0.615	0.579
rs6940159	6_170332621_T_C	0.621	0.57	0.152	0.34	0.49
rs7971	7_21940960_A_G	0.358	0.09	0.185	0.256	0.275
rs289997	7_25569548_C_T	0.148	0.041	0.227	0.153	0.156
rs74765302	7_28869017_G_A	0.109	0.189	0.002	0.079	0.112
rs13244925	7_55192256_A_C	0.565	0.323	0.71	0.506	0.486
rs17268829	7_94113799_T_C	0.298	0.044	0.255	0.374	0.265
rs4439053	7_98005235_G_A	0.171	0.047	0.153	0.102	0.112
rs111963714	7_99948655_T_G	0.216	0.196	0.032	0.142	0.182
rs71559437	7_101552440_G_A	0.135	0.035	0.056	0.089	0.087
rs7800548	7_102481842_T_C	0.338	0.761	0.613	0.485	0.46
rs12706954	7_130656911_C_T	0.385	0.449	0.313	0.401	0.381
rs68056147	7_130674481_G_A	0.305	0.095	0.255	0.335	0.255
rs201664599	7_139943702_CT_C	0.542	0.49	0.518	0.589	0.567
rs62485509	7_144048902_G_T	0.236	0.012	0.05	0.16	0.179
rs66823261	8_170692_T_C	0.219	0.288	0.193	0.234	0.231
8.17787610_CT_C	8_17787610_CT_C	0.633	0.396	0.354	0.495	0.547
rs1028016	8_23447496_A_G	0.626	0.85	0.839	0.586	0.664
rs310295	8_23663653_C_A	0.42	0.469	0.352	0.451	0.417
rs9693444	8_29509616_A_C	0.675	0.624	0.731	0.79	0.654
rs13365225	8_36858483_A_G	0.164	0.444	0.362	0.287	0.275
rs1511243	8_76230943_A_G	0.823	0.913	0.962	0.909	0.877
rs72658084	8_76333056_C_T	0.093	0.062	0.005	0.044	0.074
rs1533366	8_76378165_G_T	0.363	0.127	0.235	0.235	0.307
rs62517052	8_102483100_T_C	0.098	0.274	0.003	0.075	0.112
rs12546444	8_106358620_A_T	0.1	0.028	0.111	0.11	0.091
rs13267382	8_117209548_A_G	0.654	0.042	0.446	0.389	0.454
rs62526620	8_120862186_A_G	0.131	0.08	0.032	0.137	0.147
rs35542655	8_124563705_T_C	0.146	0.333	0.184	0.268	0.199
rs12541094	8_124571581_G_A	0.417	0.434	0.404	0.532	0.426
rs7842619	8_124739913_T_G	0.415	0.27	0.277	0.474	0.386
rs35961416	8_128213561_C_CA	0.415	0.395	0.155	0.207	0.333
rs12550713	8_128370949_C_G	0.402	0.529	0.494	0.513	0.482
rs10096351	8_128372172_A_G	0.545	0.628	0.758	0.698	0.641
rs1016578	8_129199566_G_A	0.179	0.021	0.201	0.234	0.197
rs7830152	8_143669254_A_G	0.339	0.709	0.832	0.519	0.446
rs10975870	9_6880263_A_G	0.294	0.062	0.003	0.074	0.193
rs745322029	9_21964882_CAAAA_C	0.336	0.528	0.219	0.287	0.358
rs17694493	9_22041998_C_G	0.135	0.108	0.03	0.07	0.107
rs4880038	9_36928288_T_C	0.556	0.516	0.713	0.51	0.535
rs665889	9_87782211_T_C	0.509	0.416	0.777	0.603	0.523
rs10120432	9_98362587_T_C	0.094	0.317	0.313	0.188	0.156
9.110303808_TAA_T	9_110303808_TAA_T	0.185	0.163	0.432	0.264	0.277
rs10816625	9_110837073_A_G	0.065	0.007	0.39	0.084	0.09
rs13294895	9_110837176_C_T	0.178	0.016	0.023	0.115	0.108
rs7848334	9_110849525_G_T	0.601	0.941	0.962	0.829	0.756
rs630965	9_110885479_C_T	0.615	0.485	0.909	0.84	0.676
rs1895062	9_119313486_A_G	0.407	0.127	0.298	0.276	0.324
rs3861871	9_129424719_A_G	0.455	0.476	0.573	0.496	0.461
rs550057	9_136146597_C_T	0.254	0.147	0.188	0.17	0.206
rs55910451	10_5794652_A_G	0.221	0.05	0.168	0.118	0.145
rs10796139	10_13892298_G_A	0.448	0.526	0.606	0.523	0.502
rs7072776	10_22032942_A_G	0.722	0.451	0.937	0.792	0.662
rs762131501	10_22477776_ACC_A	0.017	0.47	0.009	0.032	0.103
rs10764337	10_22861490_A_C	0.944	0.99	0.999	0.96	0.951
rs2384736	10_38523626_C_A	0.385	0.393	0.549	0.268	0.381
rs10995201	10_64299890_A_G	0.147	0.193	0.018	0.089	0.137
rs6479868	10_64819996_G_T	0.196	0.296	0.175	0.148	0.186
rs111833376	10_71335574_C_T	0.318	0.37	0.088	0.239	0.262
rs719338	10_80851257_G_T	0.595	0.76	0.634	0.711	0.664
rs4980029	10_80886726_A_G	0.159	0.444	0.405	0.373	0.289
10.95292187_CAA_C	10_95292187_CAA_C	0.81	0.706	0.643	0.741	0.77
rs10885405	10_114777670_C_T	0.467	0.809	0.028	0.392	0.501
rs12250948	10_115128491_T_C	0.787	0.217	0.239	0.555	0.613
rs9421410	10_123095209_G_A	0.318	0.547	0.405	0.283	0.379
rs45631580	10_123340107_A_G	0.059	0.255	0.103	0.108	0.119
10.123340431_GC_G	10_123340431_GC_G	0.593	0.357	0.557	0.583	0.526
rs45631563	10_123349324_A_T	0.044	0.022	0.022	0.082	0.059
rs7394715	11_433617_T_C	0.793	0.989	0.999	0.96	0.915
rs6597981	11_803017_A_G	0.519	0.116	0.233	0.23	0.367
rs4980386	11_1895708_C_A	0.378	0.159	0.695	0.395	0.395
rs10832963	11_18664241_T_G	0.745	0.094	0.503	0.619	0.597
rs4472923	11_42844441_C_T	0.329	0.396	0.23	0.279	0.308
rs10838267	11_44368892_G_A	0.546	0.142	0.33	0.427	0.43
rs77047825	11_46318032_C_G	0.073	0.003	0.001	0.007	0.039
rs12287832	11_65553492_C_A	0.192	0.051	0.117	0.062	0.133
rs10896047	11_65572431_G_A	0.472	0.155	0.225	0.559	0.44
rs35039974	11_69328130_A_T	0.204	0.041	0.236	0.198	0.181
rs661204	11_69330983_G_A	0.121	0.142	0.014	0.159	0.126
rs78540526	11_69331418_C_T	0.071	0.018	0.006	0.107	0.064
rs7125780	11_103614438_T_G	0.643	0.327	0.64	0.635	0.594
rs199504893	11_108267402_C_CA	0.41	0.146	0.413	0.517	0.442
rs610437	11_111696440_T_C	0.622	0.618	0.744	0.636	0.663
rs625145	11_116727936_A_T	0.188	0.645	0.17	0.292	0.269
rs7121616	11_122966626_A_G	0.282	0.705	0.36	0.36	0.365
rs7939702	11_129243417_T_G	0.855	0.828	0.962	0.951	0.894
rs11822830	11_129461016_A_G	0.588	0.833	0.531	0.672	0.644
rs797736	12_293626_A_G	0.369	0.436	0.437	0.481	0.391
rs12422552	12_14413931_G_C	0.269	0.435	0.303	0.361	0.312
rs788458	12_28149568_C_T	0.122	0.017	0.194	0.146	0.108
rs7297051	12_28174817_C_T	0.232	0.117	0.247	0.255	0.23
rs11049431	12_28347382_C_T	0.225	0.009	0.013	0.134	0.137
rs1027113	12_29140260_G_A	0.922	0.98	0.774	0.921	0.916
rs2277339	12_57146069_T_G	0.104	0.224	0.215	0.101	0.148
rs2870876	12_70798355_A_T	0.187	0.06	0.335	0.239	0.184
rs111622698	12_83064195_G_GA	0.106	0.019	0.15	0.104	0.101
rs10862899	12_85004551_C_T	0.511	0.122	0.129	0.384	0.414
rs17356907	12_96027759_A_G	0.303	0.175	0.256	0.276	0.263
rs7132703	12_103097887_C_T	0.117	0.307	0.001	0.069	0.14
rs11065822	12_111600134_G_T	0.356	0.018	0.005	0.088	0.192
rs1061657	12_115108136_T_C	0.259	0.049	0.432	0.207	0.23
rs11067551	12_115796577_A_G	0.196	0.435	0.298	0.335	0.294
rs2454399	12_115835836_T_C	0.423	0.449	0.285	0.565	0.469
rs206966	12_120832146_C_T	0.153	0.176	0.075	0.157	0.151
rs56404467	13_32839990_G_A	0.021	0.004	0.011	0.021	0.018
rs11571833	13_32972626_A_T	0.009	0	0.001	0.009	0.006
rs9315973	13_43501356_A_G	0.831	0.707	0.619	0.7	0.781
rs12870942	13_73806982_T_C	0.321	0.015	0.258	0.179	0.204
rs2181965	13_73960952_A_G	0.759	0.656	0.991	0.913	0.786
rs34914085	14_37128564_C_A	0.212	0.02	0.28	0.163	0.159
rs2253012	14_37228504_C_T	0.441	0.509	0.107	0.359	0.408
rs2588809	14_68660428_T_C	0.843	0.676	0.976	0.832	0.78
rs11624333	14_68979835_T_C	0.284	0.016	0.053	0.168	0.159
14.91751788_TC_T	14_91751788_TC_T	0.686	0.588	0.477	0.615	0.643
rs941764	14_91841069_A_G	0.344	0.79	0.148	0.301	0.402
rs78440108	14_93070286_C_T	0.179	0.11	0.009	0.133	0.144
rs4983544	14_105213978_T_G	0.447	0.909	0.661	0.58	0.571
rs187010898	15_46680811_C_A	0.012	0.019	0.001	0.003	0.011
rs4774565	15_50694306_A_G	0.332	0.522	0.471	0.268	0.344
rs8042593	15_66630569_G_A	0.645	0.534	0.789	0.594	0.605
rs35874463	15_67457698_A_G	0.058	0.002	0	0.041	0.041
rs8035987	15_75750383_T_C	0.256	0.673	0.357	0.415	0.428
rs2290202	15_91512267_G_T	0.13	0.157	0.49	0.205	0.178
rs144767203	15_100905819_A_C	0.108	0.244	0.227	0.146	0.172
16.4008542_CAAAAA_C	16_4008542_CAAAAA_C	0.823	0.865	0.921	0.867	0.838
rs11076805	16_4106788_C_A	0.267	0.188	0.112	0.252	0.213
rs12709163	16_6963972_C_G	0.805	0.627	0.913	0.804	0.774
rs34872983	16_10706580_G_A	0.063	0.017	0.271	0.139	0.085
rs75753503	16_23007047_G_T	0.023	0.045	0.033	0.007	0.027
rs35668161	16_52538825_C_A	0.252	0.051	0.186	0.194	0.205
rs4784227	16_52599188_C_T	0.24	0.038	0.239	0.211	0.207
rs55872725	16_53809123_C_T	0.407	0.056	0.157	0.339	0.315
rs6499648	16_53861139_C_T	0.77	0.838	0.663	0.78	0.759
rs7184573	16_53861592_G_A	0.376	0.21	0.175	0.35	0.347
rs28539243	16_54682064_G_A	0.49	0.895	0.608	0.647	0.598
rs7500067	16_80648296_A_G	0.222	0.227	0.269	0.133	0.23
rs9931038	16_85145977_T_C	0.489	0.615	0.146	0.511	0.506
rs12449271	16_87086492_T_C	0.255	0.026	0.188	0.169	0.192
rs79461387	17_29168077_G_T	0.263	0.03	0.119	0.131	0.183
rs150537328	17_39251123_T_C	0.068	0.003	0.002	0.044	0.045
rs11296	17_40127060_T_C	0.057	0.006	0.003	0.011	0.037
rs17881320	17_40485239_G_T	0.089	0.015	0.008	0.056	0.055
rs149370081	17_40744470_G_A	0.015	0.001	0	0.003	0.007
rs545502941	17_43212339_C_CT	0.217	0.242	0.12	0.177	0.23
rs2668667	17_44283858_G_A	0.17	0.007	0.003	0.056	0.097
rs2787486	17_53209774_A_C	0.313	0.447	0.304	0.206	0.297
rs745570	17_77781725_A_G	0.513	0.829	0.398	0.591	0.579
rs16976596	18_11696613_C_T	0.131	0.762	0.026	0.117	0.246
rs11665269	18_20634253_C_T	0.631	0.078	0.476	0.56	0.529
rs1111207	18_24125857_T_C	0.432	0.366	0.646	0.419	0.433
rs527616	18_24337424_C_G	0.642	0.882	0.707	0.709	0.705
18.24518050_AT_A	18_24518050_AT_A	0.287	0.183	0.478	0.208	0.249
rs8092192	18_25407513_C_G	0.702	0.399	0.492	0.585	0.617
rs72931898	18_29981526_G_A	0.045	0.187	0.018	0.044	0.053
rs9954058	18_42411803_G_C	0.08	0.347	0.102	0.101	0.141
rs9952980	18_42888797_T_C	0.36	0.925	0.302	0.393	0.449
rs117922601	19_13249921_G_T	0.056	0.006	0	0.008	0.021
rs56069439	19_17393925_C_A	0.295	0.243	0.007	0.152	0.212
rs10164323	19_18569492_C_T	0.354	0.807	0.214	0.424	0.42
rs140702307	19_19517054_C_CGGGCG	0.356	0.836	0.379	0.616	0.527
rs56681946	19_44283031_T_C	0.376	0.22	0.149	0.447	0.337
rs4399645	19_46166073_T_C	0.592	0.65	0.4	0.688	0.629
rs1172821	19_55816678_C_T	0.373	0.458	0.1	0.499	0.392
rs16991615	20_5948227_G_A	0.066	0.001	0	0.025	0.039
rs1154723	20_11379842_T_C	0.95	0.999	0.999	0.991	0.983
rs6030585	20_41613706_C_G	0.795	0.986	0.845	0.905	0.863
rs13039563	20_52296849_G_A	0.242	0.053	0.326	0.321	0.257
rs2822999	21_16364756_T_G	0.181	0.016	0.168	0.13	0.129
rs2823130	21_16566350_A_G	0.086	0.219	0.093	0.108	0.088
rs2403907	21_16574455_C_A	0.315	0.439	0.121	0.34	0.368
rs4818836	21_47762932_G_A	0.032	0.001	0.015	0.035	0.027
rs9798754	22_19766137_C_T	0.372	0.453	0.571	0.591	0.494
rs17879961	22_29121087_A_G	0.001	0	0	0	0.001
rs5997390	22_29135543_G_A	0.081	0.067	0.09	0.106	0.069
rs34134147	22_29203724_C_T	0.022	0.001	0.001	0.011	0.019
rs132289	22_29551872_A_G	0.979	0.998	1	0.996	0.992
rs5750715	22_39343916_T_A	0.251	0.169	0.494	0.36	0.269
22.40904707_CT_C	22_40904707_CT_C	0.095	0.138	0.214	0.121	0.128
rs9611990	22_43433100_C_T	0.121	0.011	0.004	0.08	0.071
rs112855987	22_45319953_G_A	0.42	0.257	0.251	0.288	0.32
rs28512361	22_46283297_G_A	0.108	0.298	0.04	0.083	0.123

Table S8. Allele frequencies (EAFs) by genetic ancestry for SNPs included in the PGS models. EAFs were estimated in UK Biobank individuals without a diagnosis of cancer or mastectomy prior to the start of follow-up. Genetic ancestry groupings are according to iadmix, derived as described in the Methods: EUR, European; AFR, African; EAS, East Asian; SAS, South Asian. [a]SNP variant name. ***Subtable S8c***: EAFs on all UK Biobank individuals.

UKB rs number	SNP[a]	EAFs on UK Biobank men and women				
		EUR	*AFR*	*EAS*	*SAS*	*Mixed*
rs707475	1_7917076_G_A	0.392	0.509	0.215	0.263	0.363
rs616488	1_10566215_A_G	0.345	0.089	0.332	0.227	0.255
rs2992756	1_18807339_T_C	0.5	0.462	0.853	0.445	0.511
rs4233486	1_41380440_C_T	0.658	0.761	0.666	0.649	0.662
rs114282204	1_41389220_T_C	0.018	0.004	0.003	0.016	0.016
1.46670206_TC_T	1_46670206_TC_T	0.302	0.079	0.018	0.098	0.172
1.51467096_CT_C	1_51467096_CT_C	0.491	0.534	0.317	0.445	0.502
rs17426269	1_88156923_G_A	0.154	0.101	0.008	0.119	0.134
rs2151842	1_88428199_C_A	0.239	0.157	0.112	0.252	0.201
rs612683	1_100880328_A_T	0.403	0.235	0.541	0.458	0.395
1.110198129_CAAA_C	1_110198129_CAAA_C	0.788	0.4	0.935	0.791	0.726
rs7513707	1_114445880_G_A	0.174	0.016	0.586	0.142	0.173
rs12406858	1_118141492_A_C	0.255	0.352	0.414	0.349	0.334
rs637868	1_120257110_T_C	0.528	0.54	0.919	0.712	0.608
rs11249433	1_121280613_A_G	0.416	0.071	0.028	0.21	0.294
rs111458676	1_121287994_A_G	0.093	0.033	0.171	0.298	0.165
rs143384623	1_145604302_C_CT	0.355	0.093	0.173	0.273	0.252
rs11205303	1_149906413_T_C	0.406	0.03	0.332	0.333	0.304
rs12091730	1_155556971_G_A	0.23	0.043	0.664	0.269	0.226
rs761575824	1_168171052_CA_C	0.101	0.242	0.004	0.039	0.112
rs11463354	1_172328767_T_TA	0.323	0.498	0.134	0.273	0.326
rs35383942	1_201437832_C_T	0.062	0.003	0	0.007	0.028
rs6686987	1_202184600_C_T	0.403	0.261	0.245	0.287	0.309
rs7514172	1_203770448_T_A	0.275	0.179	0.352	0.301	0.263
rs11268668	1_204502514_T_TTCTGAAACAGGG	0.782	0.979	0.987	0.926	0.868
rs2785646	1_208076291_G_A	0.35	0.089	0.022	0.156	0.212
rs2576261	1_217053815_T_G	0.329	0.847	0.162	0.288	0.411
rs11117758	1_217220574_G_A	0.211	0.142	0.047	0.261	0.212
rs11118563	1_220671050_C_T	0.233	0.018	0.314	0.186	0.208
rs72755295	1_242034263_A_G	0.033	0.002	0.001	0.023	0.019
rs78425380	2_10138983_T_C	0.11	0.095	0.006	0.073	0.09
rs6743383	2_19315675_T_A	0.558	0.451	0.597	0.363	0.471
rs6725517	2_25129473_A_G	0.432	0.817	0.399	0.405	0.469
rs12472404	2_29179452_G_C	0.231	0.166	0.785	0.298	0.281
rs4322799	2_29615233_T_C	0.238	0.29	0.034	0.296	0.259
rs553796823	2_39699510_C_CT	0.462	0.225	0.822	0.603	0.491
rs6756513	2_70172587_G_A	0.281	0.267	0.277	0.276	0.231
rs1036759	2_88358825_G_C	0.305	0.477	0.272	0.281	0.342
rs6746250	2_121058254_A_G	0.691	0.824	0.415	0.608	0.693
rs17625845	2_121089731_T_C	0.195	0.045	0.082	0.109	0.148
rs10164550	2_121159205_G_A	0.357	0.674	0.138	0.392	0.412
rs10179592	2_121246568_T_C	0.9	0.68	0.765	0.79	0.821
rs17726078	2_172974566_C_G	0.479	0.228	0.212	0.362	0.365
rs1550622	2_174212910_A_G	0.832	0.746	0.99	0.879	0.844
rs2356656	2_192381934_C_T	0.873	0.677	0.936	0.939	0.865
rs10197246	2_202204741_T_C	0.732	0.792	0.698	0.833	0.729
rs4442975	2_217920769_G_T	0.511	0.304	0.888	0.469	0.469
2.217955896_GA_G	2_217955896_GA_G	0.036	0.154	0	0.03	0.047
rs11693806	2_218292158_C_G	0.735	0.495	0.374	0.399	0.574
rs3791977	2_218714845_G_A	0.399	0.198	0.26	0.322	0.3
rs4676356	2_241388857_C_A	0.973	0.999	0.999	0.987	0.985
rs6762558	3_4742251_A_G	0.408	0.26	0.09	0.116	0.241
rs552647	3_27353716_C_A	0.525	0.967	0.262	0.507	0.617
rs62255657	3_27388664_C_G	0.256	0.265	0.147	0.309	0.315
rs112476261	3_29294845_C_T	0.015	0.001	0	0.008	0.01
rs17838698	3_30684907_C_T	0.287	0.298	0.688	0.354	0.33
rs56387622	3_46888198_T_C	0.1	0.334	0.145	0.231	0.187
rs371314787	3_49709912_C_CT	0.289	0.241	0.05	0.232	0.252
rs138866686	3_55970777_A_AT	0.025	0.002	0.002	0.007	0.019
rs2886671	3_59373745_C_T	0.421	0.507	0.687	0.463	0.459
rs73130576	3_63887449_T_TTG	0.138	0.02	0.141	0.132	0.122
rs9825432	3_71620370_T_G	0.638	0.595	0.038	0.441	0.526
rs13066793	3_87037543_A_G	0.09	0.034	0.006	0.075	0.063
rs639355	3_99403877_G_A	0.481	0.259	0.483	0.369	0.407
3.141112859_CTT_C	3_141112859_CTT_C	0.428	0.85	0.067	0.202	0.395
rs58058861	3_172285237_G_A	0.22	0.141	0.318	0.238	0.211
rs9882792	3_189774456_C_T	0.221	0.13	0.075	0.196	0.17
rs10012017	4_38784633_G_T	0.204	0.505	0.439	0.411	0.394
rs17014016	4_89240476_G_A	0.444	0.443	0.014	0.27	0.355
rs775780062	4_92594859_TTCTTTC_T	0.453	0.459	0.59	0.34	0.432
rs62331150	4_106069013_G_T	0.205	0.015	0.629	0.297	0.238
rs56039025	4_143467195_C_T	0.113	0.153	0.045	0.119	0.118
rs745707748	4_151218296_CATATTT_C	0.64	0.425	0.234	0.449	0.51
rs28436676	4_175842495_G_A	0.119	0.459	0.259	0.19	0.21
rs62334414	4_175847436_C_A	0.341	0.148	0.027	0.295	0.248
4.187513623_TA_T	4_187503758_A_T	0.461	0.336	0.39	0.335	0.425
rs62641919	5_345109_T_C	0.06	0.632	0.006	0.061	0.159
rs10069690	5_1279790_C_T	0.258	0.652	0.18	0.255	0.32
rs3215401	5_1296255_A_AG	0.314	0.095	0.352	0.554	0.349
rs62329727	5_1353077_T_C	0.012	0.001	0.002	0.002	0.005
rs4866496	5_2777029_G_A	0.421	0.532	0.79	0.467	0.478
rs17611291	5_16231194_G_C	0.552	0.174	0.165	0.414	0.416
rs770436441	5_32579616_TCA_T	0.475	0.133	0.115	0.368	0.383
rs138335056	5_44508264_G_GT	0.115	0.251	0.006	0.115	0.149
rs187108781	5_44619502_A_G	0.157	0.008	0.002	0.047	0.089
rs4613718	5_44649944_C_T	0.606	0.754	0.603	0.671	0.655
rs10941679	5_44706498_A_G	0.252	0.177	0.528	0.354	0.282
rs17343002	5_44853593_G_C	0.314	0.168	0.054	0.128	0.222
rs10940312	5_52679539_C_CA	0.103	0.02	0.2	0.114	0.101
rs553874618	5_55662540_C_CT	0.36	0.265	0.372	0.37	0.371
rs889310	5_55965167_C_T	0.561	0.61	0.561	0.683	0.619
rs16886165	5_56023083_T_G	0.161	0.345	0.368	0.329	0.24
rs76250845	5_56042972_C_T	0.059	0.057	0.111	0.033	0.05
rs11949391	5_56045081_T_C	0.166	0.084	0.044	0.18	0.145
rs113778879	5_58241712_C_T	0.573	0.571	0.678	0.603	0.588
rs3010266	5_71965007_G_A	0.261	0.209	0.117	0.32	0.237
rs157557	5_73234583_T_C	0.33	0.251	0.425	0.409	0.339
rs767431357	5_77155397_GT_G	0.346	0.029	0.193	0.267	0.254
rs34525310	5_79180995_G_GA	0.175	0.108	0.376	0.245	0.166
5.81512947_TA_T	5_81512947_TA_T	0.251	0.099	0.002	0.103	0.177
rs332529	5_90789470_G_A	0.152	0.311	0.489	0.384	0.257
rs17157372	5_104300273_G_T	0.17	0.414	0.057	0.182	0.218
rs335160	5_122478676_C_A	0.75	0.698	0.61	0.615	0.704
rs1428387	5_122705244_C_T	0.023	0.06	0.09	0.108	0.055
rs6860806	5_131640536_A_G	0.551	0.372	0.263	0.206	0.402
rs6596100	5_132407058_C_T	0.236	0.283	0.123	0.249	0.241
rs1432679	5_158244083_C_T	0.554	0.168	0.389	0.672	0.494
rs10074269	5_169591460_T_C	0.344	0.313	0.48	0.437	0.382
rs6864691	5_173358154_G_A	0.422	0.448	0.316	0.528	0.451
rs4868701	5_176134882_T_C	0.54	0.746	0.509	0.558	0.582
rs418053	6_13713366_G_C	0.574	0.591	0.4	0.555	0.567
rs3819405	6_16399557_C_T	0.33	0.474	0.361	0.488	0.4
rs12211970	6_18783140_G_A	0.61	0.471	0.714	0.702	0.603
rs769485514	6_20537845_CA_C	0.492	0.755	0.354	0.366	0.496
rs9358466	6_21923810_T_C	0.433	0.284	0.318	0.389	0.407
rs34196306	6_27425644_G_C	0.107	0.006	0	0	0.028
rs111342015	6_43227141_G_A	0.099	0.006	0.005	0.09	0.065
rs10623112	6_82263549_AAT_A	0.416	0.281	0.387	0.416	0.406
6.85912194_CAA_C	6_85912194_CAA_C	0.065	0.005	0.001	0.015	0.032
rs73754909	6_87803819_T_C	0.277	0.73	0.258	0.277	0.366
rs55941023	6_130341728_C_CT	0.699	0.742	0.912	0.675	0.742
rs2121348	6_149595505_T_C	0.202	0.179	0.43	0.21	0.224
rs6913578	6_151949806_A_C	0.323	0.504	0.339	0.346	0.362
rs60954078	6_151955914_A_G	0.072	0.384	0.303	0.068	0.147
6.152022664_CAAAAAAA_C	6_152022664_CAAAAAAA_C	0.614	0.718	0.408	0.517	0.581
rs851984	6_152023191_G_A	0.391	0.225	0.122	0.228	0.299
rs6904031	6_152055978_A_T	0.057	0.177	0.075	0.047	0.08
rs910416	6_152432902_C_T	0.509	0.527	0.552	0.493	0.482
rs9364472	6_169006947_C_G	0.51	0.473	0.41	0.612	0.57
rs6940159	6_170332621_T_C	0.621	0.572	0.165	0.342	0.497
rs7971	7_21940960_A_G	0.359	0.088	0.189	0.26	0.269
rs289997	7_25569548_C_T	0.147	0.04	0.233	0.154	0.149
rs74765302	7_28869017_G_A	0.109	0.192	0.002	0.082	0.113
rs13244925	7_55192256_A_C	0.565	0.321	0.704	0.503	0.491
rs17268829	7_94113799_T_C	0.298	0.048	0.257	0.371	0.266
rs4439053	7_98005235_G_A	0.171	0.052	0.152	0.104	0.117
rs111963714	7_99948655_T_G	0.214	0.19	0.036	0.139	0.18
rs71559437	7_101552440_G_A	0.135	0.037	0.071	0.087	0.091
rs7800548	7_102481842_T_C	0.338	0.756	0.611	0.483	0.468
rs12706954	7_130656911_C_T	0.385	0.449	0.298	0.403	0.389
rs68056147	7_130674481_G_A	0.305	0.096	0.265	0.34	0.256
rs201664599	7_139943702_CT_C	0.542	0.485	0.534	0.586	0.564
rs62485509	7_144048902_G_T	0.235	0.014	0.045	0.161	0.176
rs66823261	8_170692_T_C	0.219	0.29	0.197	0.238	0.235
8.17787610_CT_C	8_17787610_CT_C	0.633	0.4	0.349	0.497	0.539
rs1028016	8_23447496_A_G	0.626	0.844	0.824	0.579	0.677
rs310295	8_23663653_C_A	0.42	0.474	0.336	0.449	0.426
rs9693444	8_29509616_A_C	0.676	0.62	0.728	0.793	0.669
rs13365225	8_36858483_A_G	0.164	0.443	0.355	0.297	0.277
rs1511243	8_76230943_A_G	0.823	0.913	0.966	0.908	0.881
rs72658084	8_76333056_C_T	0.093	0.061	0.006	0.045	0.075
rs1533366	8_76378165_G_T	0.362	0.131	0.237	0.242	0.295
rs62517052	8_102483100_T_C	0.098	0.273	0.004	0.077	0.115
rs12546444	8_106358620_A_T	0.099	0.026	0.117	0.111	0.092
rs13267382	8_117209548_A_G	0.653	0.046	0.456	0.389	0.445
rs62526620	8_120862186_A_G	0.132	0.078	0.034	0.135	0.136
rs35542655	8_124563705_T_C	0.146	0.332	0.18	0.266	0.198
rs12541094	8_124571581_G_A	0.416	0.428	0.393	0.53	0.427
rs7842619	8_124739913_T_G	0.414	0.272	0.265	0.48	0.382
rs35961416	8_128213561_C_CA	0.415	0.397	0.141	0.205	0.334
rs12550713	8_128370949_C_G	0.402	0.53	0.52	0.509	0.479
rs10096351	8_128372172_A_G	0.544	0.626	0.752	0.697	0.635
rs1016578	8_129199566_G_A	0.179	0.023	0.201	0.235	0.188
rs7830152	8_143669254_A_G	0.338	0.704	0.815	0.52	0.463
rs10975870	9_6880263_A_G	0.293	0.065	0.003	0.075	0.186
rs745322029	9_21964882_CAAAA_C	0.336	0.524	0.225	0.287	0.368
rs17694493	9_22041998_C_G	0.135	0.109	0.03	0.069	0.108
rs4880038	9_36928288_T_C	0.557	0.517	0.731	0.508	0.541
rs665889	9_87782211_T_C	0.509	0.42	0.788	0.6	0.534
rs10120432	9_98362587_T_C	0.094	0.317	0.311	0.187	0.166
9.110303808_TAA_T	9_110303808_TAA_T	0.185	0.161	0.434	0.26	0.264
rs10816625	9_110837073_A_G	0.064	0.007	0.396	0.079	0.088
rs13294895	9_110837176_C_T	0.178	0.017	0.023	0.119	0.108
rs7848334	9_110849525_G_T	0.601	0.942	0.959	0.829	0.762
rs630965	9_110885479_C_T	0.616	0.489	0.921	0.84	0.676
rs1895062	9_119313486_A_G	0.407	0.124	0.302	0.278	0.321
rs3861871	9_129424719_A_G	0.454	0.481	0.567	0.498	0.465
rs550057	9_136146597_C_T	0.255	0.146	0.197	0.172	0.213
rs55910451	10_5794652_A_G	0.222	0.051	0.16	0.119	0.156
rs10796139	10_13892298_G_A	0.448	0.521	0.626	0.524	0.499
rs7072776	10_22032942_A_G	0.722	0.461	0.933	0.792	0.664
rs762131501	10_22477776_ACC_A	0.017	0.467	0.008	0.033	0.111
rs10764337	10_22861490_A_C	0.944	0.99	0.999	0.96	0.955
rs2384736	10_38523626_C_A	0.385	0.397	0.56	0.267	0.386
rs10995201	10_64299890_A_G	0.148	0.195	0.02	0.089	0.134
rs6479868	10_64819996_G_T	0.196	0.302	0.172	0.146	0.195
rs111833376	10_71335574_C_T	0.318	0.366	0.092	0.238	0.267
rs719338	10_80851257_G_T	0.596	0.765	0.626	0.71	0.659
rs4980029	10_80886726_A_G	0.159	0.435	0.41	0.373	0.298
10.95292187_CAA_C	10_95292187_CAA_C	0.81	0.705	0.642	0.747	0.77
rs10885405	10_114777670_C_T	0.466	0.806	0.035	0.393	0.498
rs12250948	10_115128491_T_C	0.788	0.222	0.234	0.556	0.596
rs9421410	10_123095209_G_A	0.317	0.541	0.4	0.282	0.383
rs45631580	10_123340107_A_G	0.059	0.25	0.098	0.111	0.122
10.123340431_GC_G	10_123340431_GC_G	0.595	0.357	0.545	0.588	0.53
rs45631563	10_123349324_A_T	0.045	0.019	0.022	0.085	0.057
rs7394715	11_433617_T_C	0.794	0.988	0.998	0.961	0.908
rs6597981	11_803017_A_G	0.518	0.118	0.243	0.226	0.361
rs4980386	11_1895708_C_A	0.379	0.159	0.694	0.396	0.389
rs10832963	11_18664241_T_G	0.745	0.101	0.497	0.612	0.585
rs4472923	11_42844441_C_T	0.33	0.399	0.236	0.274	0.309
rs10838267	11_44368892_G_A	0.546	0.144	0.331	0.426	0.421
rs77047825	11_46318032_C_G	0.073	0.003	0.001	0.007	0.038
rs12287832	11_65553492_C_A	0.191	0.046	0.123	0.058	0.127
rs10896047	11_65572431_G_A	0.473	0.156	0.212	0.557	0.424
rs35039974	11_69328130_A_T	0.204	0.042	0.234	0.203	0.182
rs661204	11_69330983_G_A	0.121	0.145	0.02	0.158	0.126
rs78540526	11_69331418_C_T	0.071	0.016	0.008	0.106	0.062
rs7125780	11_103614438_T_G	0.644	0.338	0.653	0.635	0.589
rs199504893	11_108267402_C_CA	0.41	0.14	0.404	0.521	0.43
rs610437	11_111696440_T_C	0.621	0.617	0.759	0.638	0.657
rs625145	11_116727936_A_T	0.189	0.647	0.169	0.289	0.283
rs7121616	11_122966626_A_G	0.281	0.704	0.351	0.357	0.381
rs7939702	11_129243417_T_G	0.856	0.83	0.964	0.951	0.887
rs11822830	11_129461016_A_G	0.587	0.833	0.537	0.674	0.645
rs797736	12_293626_A_G	0.368	0.433	0.441	0.478	0.393
rs12422552	12_14413931_G_C	0.268	0.434	0.287	0.359	0.313
rs788458	12_28149568_C_T	0.123	0.016	0.189	0.148	0.105
rs7297051	12_28174817_C_T	0.233	0.118	0.243	0.255	0.228
rs11049431	12_28347382_C_T	0.225	0.012	0.01	0.135	0.131
rs1027113	12_29140260_G_A	0.922	0.98	0.777	0.92	0.921
rs2277339	12_57146069_T_G	0.104	0.223	0.194	0.098	0.143
rs2870876	12_70798355_A_T	0.187	0.064	0.338	0.239	0.18
rs111622698	12_83064195_G_GA	0.106	0.02	0.158	0.104	0.099
rs10862899	12_85004551_C_T	0.511	0.129	0.127	0.389	0.4
rs17356907	12_96027759_A_G	0.304	0.179	0.26	0.279	0.261
rs7132703	12_103097887_C_T	0.117	0.305	0.003	0.067	0.139
rs11065822	12_111600134_G_T	0.355	0.02	0.006	0.086	0.19
rs1061657	12_115108136_T_C	0.258	0.05	0.445	0.205	0.229
rs11067551	12_115796577_A_G	0.196	0.434	0.296	0.332	0.296
rs2454399	12_115835836_T_C	0.423	0.457	0.28	0.57	0.466
rs206966	12_120832146_C_T	0.154	0.173	0.076	0.16	0.151
rs56404467	13_32839990_G_A	0.021	0.004	0.01	0.021	0.018
rs11571833	13_32972626_A_T	0.009	0.001	0.001	0.008	0.006
rs9315973	13_43501356_A_G	0.831	0.714	0.615	0.702	0.778
rs12870942	13_73806982_T_C	0.321	0.02	0.257	0.181	0.206
rs2181965	13_73960952_A_G	0.758	0.657	0.992	0.912	0.786
rs34914085	14_37128564_C_A	0.212	0.022	0.285	0.163	0.16
rs2253012	14_37228504_C_T	0.441	0.509	0.109	0.361	0.41
rs2588809	14_68660428_T_C	0.842	0.677	0.978	0.833	0.784
rs11624333	14_68979835_T_C	0.284	0.018	0.051	0.166	0.159
14.91751788_TC_T	14_91751788_TC_T	0.685	0.592	0.49	0.612	0.636
rs941764	14_91841069_A_G	0.342	0.785	0.135	0.298	0.412
rs78440108	14_93070286_C_T	0.179	0.107	0.008	0.132	0.14
rs4983544	14_105213978_T_G	0.447	0.905	0.655	0.581	0.583
rs187010898	15_46680811_C_A	0.012	0.018	0.001	0.003	0.01
rs4774565	15_50694306_A_G	0.331	0.524	0.496	0.265	0.353
rs8042593	15_66630569_G_A	0.646	0.53	0.796	0.597	0.609
rs35874463	15_67457698_A_G	0.057	0.003	0	0.041	0.04
rs8035987	15_75750383_T_C	0.257	0.666	0.36	0.413	0.425
rs2290202	15_91512267_G_T	0.13	0.153	0.501	0.207	0.179
rs144767203	15_100905819_A_C	0.108	0.251	0.226	0.147	0.174
16.4008542_CAAAAA_C	16_4008542_CAAAAA_C	0.823	0.863	0.929	0.867	0.839
rs11076805	16_4106788_C_A	0.267	0.189	0.108	0.253	0.217
rs12709163	16_6963972_C_G	0.805	0.628	0.912	0.806	0.772
rs34872983	16_10706580_G_A	0.063	0.017	0.268	0.137	0.084
rs75753503	16_23007047_G_T	0.023	0.045	0.027	0.008	0.027
rs35668161	16_52538825_C_A	0.251	0.051	0.184	0.192	0.196
rs4784227	16_52599188_C_T	0.239	0.038	0.239	0.207	0.2
rs55872725	16_53809123_C_T	0.405	0.058	0.154	0.339	0.308
rs6499648	16_53861139_C_T	0.771	0.838	0.676	0.774	0.765
rs7184573	16_53861592_G_A	0.377	0.206	0.191	0.351	0.346
rs28539243	16_54682064_G_A	0.489	0.884	0.589	0.643	0.609
rs7500067	16_80648296_A_G	0.223	0.229	0.256	0.131	0.23
rs9931038	16_85145977_T_C	0.489	0.606	0.147	0.508	0.498
rs12449271	16_87086492_T_C	0.255	0.028	0.176	0.166	0.185
rs79461387	17_29168077_G_T	0.264	0.032	0.108	0.126	0.171
rs150537328	17_39251123_T_C	0.068	0.004	0.002	0.043	0.043
rs11296	17_40127060_T_C	0.057	0.007	0.002	0.011	0.034
rs17881320	17_40485239_G_T	0.09	0.014	0.009	0.055	0.053
rs149370081	17_40744470_G_A	0.015	0.001	0	0.003	0.007
rs545502941	17_43212339_C_CT	0.218	0.238	0.125	0.175	0.223
rs2668667	17_44283858_G_A	0.171	0.01	0.002	0.057	0.093
rs2787486	17_53209774_A_C	0.314	0.442	0.308	0.208	0.304
rs745570	17_77781725_A_G	0.514	0.826	0.411	0.586	0.585
rs16976596	18_11696613_C_T	0.132	0.757	0.029	0.115	0.251
rs11665269	18_20634253_C_T	0.63	0.083	0.471	0.558	0.509
rs1111207	18_24125857_T_C	0.431	0.361	0.651	0.415	0.431
rs527616	18_24337424_C_G	0.641	0.88	0.705	0.711	0.712
18.24518050_AT_A	18_24518050_AT_A	0.287	0.178	0.479	0.204	0.255
rs8092192	18_25407513_C_G	0.703	0.393	0.472	0.576	0.612
rs72931898	18_29981526_G_A	0.045	0.181	0.016	0.044	0.057
rs9954058	18_42411803_G_C	0.079	0.345	0.123	0.102	0.144
rs9952980	18_42888797_T_C	0.359	0.919	0.294	0.387	0.459
rs117922601	19_13249921_G_T	0.056	0.006	0	0.008	0.021
rs56069439	19_17393925_C_A	0.294	0.234	0.005	0.155	0.21
rs10164323	19_18569492_C_T	0.354	0.807	0.215	0.425	0.429
rs140702307	19_19517054_C_CGGGCG	0.355	0.838	0.378	0.615	0.524
rs56681946	19_44283031_T_C	0.376	0.22	0.137	0.442	0.329
rs4399645	19_46166073_T_C	0.592	0.644	0.401	0.688	0.629
rs1172821	19_55816678_C_T	0.372	0.446	0.101	0.494	0.391
rs16991615	20_5948227_G_A	0.065	0.002	0	0.025	0.039
rs1154723	20_11379842_T_C	0.95	0.998	0.999	0.991	0.981
rs6030585	20_41613706_C_G	0.795	0.983	0.846	0.906	0.867
rs13039563	20_52296849_G_A	0.242	0.053	0.325	0.319	0.246
rs2822999	21_16364756_T_G	0.18	0.018	0.156	0.136	0.129
rs2823130	21_16566350_A_G	0.087	0.219	0.098	0.104	0.097
rs2403907	21_16574455_C_A	0.315	0.437	0.113	0.337	0.361
rs4818836	21_47762932_G_A	0.032	0.002	0.015	0.033	0.028
rs9798754	22_19766137_C_T	0.371	0.46	0.56	0.59	0.481
rs17879961	22_29121087_A_G	0.001	0	0	0	0.001
rs5997390	22_29135543_G_A	0.08	0.066	0.083	0.105	0.07
rs34134147	22_29203724_C_T	0.022	0.002	0	0.01	0.016
rs132289	22_29551872_A_G	0.978	0.998	1	0.996	0.991
rs5750715	22_39343916_T_A	0.251	0.169	0.5	0.362	0.267
22.40904707_CT_C	22_40904707_CT_C	0.095	0.134	0.214	0.122	0.122
rs9611990	22_43433100_C_T	0.121	0.011	0.003	0.079	0.075
rs112855987	22_45319953_G_A	0.42	0.256	0.247	0.287	0.326
rs28512361	22_46283297_G_A	0.107	0.3	0.038	0.087	0.125

Table S9. Mean and standard deviation (SD) of the 309-SNP BC PGS in UK Biobank individuals without a diagnosis of cancer or mastectomy prior to the start of follow-up. Genetic ancestry groupings are according to iadmix, derived as described in the Methods; self-reported ethnicity groupings are as described in the Methods.

	**Women**				
**Genetic ancestry**	*N*	*mean*	*L95%CI*	*U95%CI*	*SD*
European	212880	-0.341	-0.344	-0.338	0.613
African	4166	0.294	0.277	0.310	0.547
East Asian	1524	0.272	0.245	0.300	0.552
South Asian	3771	-0.172	-0.191	-0.153	0.593
Mixed	3999	-0.067	-0.086	-0.048	0.623
**Self-reported ethnicity**					
White	212256	-0.341	-0.343	-0.338	0.613
Black	4146	0.270	0.253	0.287	0.553
East Asian	881	0.261	0.226	0.297	0.536
South Asian	3366	-0.171	-0.192	-0.151	0.597
Mixed	1629	-0.119	-0.149	-0.089	0.616
Don't know	718	-0.213	-0.261	-0.166	0.643
Other	3344	0.014	-0.007	0.036	0.638
	**Men**				
**Genetic ancestry**	*N*	*mean*	*L95%CI*	*U95%CI*	*SD*
European	188228	-0.327	-0.330	-0.325	0.617
African	3013	0.289	0.269	0.308	0.547
East Asian	760	0.260	0.220	0.300	0.563
South Asian	4469	-0.150	-0.168	-0.132	0.609
Mixed	3603	-0.065	-0.085	-0.045	0.615
**Self-reported ethnicity**					
White	187427	-0.327	-0.33	-0.324	0.617
Black	3125	0.265	0.245	0.285	0.561
East Asian	549	0.252	0.205	0.299	0.558
South Asian	4006	-0.155	-0.174	-0.136	0.615
Mixed	1026	-0.110	-0.148	-0.072	0.619
Don't know	936	-0.217	-0.258	-0.176	0.636
Other	3004	-0.054	-0.076	-0.032	0.615
	**Men and Women**				
**Genetic ancestry**	*N*	*mean*	*L95%CI*	*U95%CI*	*SD*
European	401108	-0.335	-0.336	-0.333	0.615
African	7179	0.292	0.279	0.304	0.547
East Asian	2284	0.268	0.246	0.291	0.555
South Asian	8240	-0.160	-0.173	-0.147	0.602
Mixed	7602	-0.066	-0.08	-0.052	0.619
**Self-reported ethnicity**					
White	399683	-0.334	-0.336	-0.333	0.615
Black	7271	0.268	0.255	0.28	0.557
East Asian	1430	0.258	0.229	0.286	0.545
South Asian	7372	-0.163	-0.176	-0.149	0.607
Mixed	2655	-0.116	-0.139	-0.092	0.617
Don't know	1654	-0.216	-0.246	-0.185	0.639
Other	6348	-0.018	-0.034	-0.003	0.628

Table S10. Mean and standard deviation (SD) of the 307-SNP BC PGS in UK Biobank individuals without a diagnosis of cancer or mastectomy prior to the start of follow-up. Genetic ancestry groupings are according to iadmix, derived as described in the Methods; self-reported ethnicity groupings are as described in the Methods. The 307-SNP PGS does not include 22_29203724_C_T and 22_29551872_A_G.

	**Women**				
**Genetic ancestry**	*N*	*mean*	*L95%CI*	*U95%CI*	*SD*
European	212880	-0.011	-0.014	-0.009	0.611
African	4166	0.636	0.619	0.652	0.547
East Asian	1524	0.615	0.588	0.643	0.552
South Asian	3771	0.167	0.148	0.186	0.592
Mixed	3999	0.269	0.25	0.289	0.623
**Self-reported ethnicity**					
White	212256	-0.011	-0.014	-0.009	0.611
Black	4146	0.612	0.595	0.629	0.553
East Asian	881	0.604	0.569	0.64	0.537
South Asian	3366	0.167	0.147	0.187	0.596
Mixed	1629	0.216	0.186	0.246	0.617
Don't know	718	0.121	0.073	0.168	0.644
Other	3344	0.353	0.331	0.374	0.639

	**Men**				
**Genetic ancestry**	*N*	*mean*	*L95%CI*	*U95%CI*	*SD*
European	188228	0.002	0.000	0.005	0.615
African	3013	0.631	0.611	0.65	0.547
East Asian	760	0.603	0.563	0.643	0.563
South Asian	4469	0.188	0.171	0.206	0.609
Mixed	3603	0.270	0.250	0.29	0.615
**Self-reported ethnicity**					
White	187427	0.003	0.000	0.005	0.615
Black	3125	0.606	0.587	0.626	0.561
East Asian	549	0.594	0.548	0.641	0.558
South Asian	4006	0.183	0.164	0.202	0.614
Mixed	1026	0.223	0.185	0.261	0.619
Don't know	936	0.117	0.076	0.158	0.634
Other	3004	0.283	0.261	0.305	0.616

	**Men and Women**				
**Genetic ancestry**	*N*	*mean*	*L95%CI*	*U95%CI*	*SD*
European	401108	-0.005	-0.007	-0.003	0.613
African	7179	0.634	0.621	0.646	0.547
East Asian	2284	0.611	0.589	0.634	0.555
South Asian	8240	0.179	0.166	0.192	0.601
Mixed	7602	0.270	0.256	0.284	0.619
**Self-reported ethnicity**					
White	399683	-0.005	-0.007	-0.003	0.613
Black	7271	0.609	0.597	0.622	0.557
East Asian	1430	0.600	0.572	0.629	0.545
South Asian	7372	0.176	0.162	0.19	0.606
Mixed	2655	0.219	0.195	0.242	0.617
Don't know	1654	0.118	0.088	0.149	0.638
Other	6348	0.319	0.304	0.335	0.629

Table S11. Self-reported vs genetic ancestry (iadmix) in UK Biobank for individuals without a diagnosis of cancer. Genetic ancestry groupings are according to iadmix, derived as described in the Methods: EUR, European; AFR, African; EAS, East Asian; SAS, South Asian. Self-reported ethnicity groupings are as described in the Methods. In total, 226340 women, 200073 men and 426413 men and women were included in each analysis.

**Women**					
**Ethnicity** \ **Ancestry**	*EUR*	*AFR*	*EAS*	*SAS*	*Mixed*
White	211584	2	2	3	665
Black	2	3629	0	15	500
East Asian	0	0	860	0	21
South Asian	2	0	0	2991	373
Mixed	276	17	5	28	1303
Don't know	455	85	21	45	112
Other	561	433	636	689	1025
Total	212880	4166	1524	3771	3999
Grand total					226340

**Men**					
**Ethnicity** \ **Ancestry**	*EUR*	*AFR*	*EAS*	*SAS*	*Mixed*
White	186848	1	0	5	573
Black	2	2673	0	12	438
East Asian	1	0	528	0	20
South Asian	3	0	0	3585	418
Mixed	187	11	2	15	811
Don't know	724	40	6	58	108
Other	463	288	224	794	1235
Total	188228	3013	760	4469	3603
Grand total					200073

**Men and women**					
**Ethnicity** \ **Ancestry**	*EUR*	*AFR*	*EAS*	*SAS*	*Mixed*
White	398432	3	2	8	1238
Black	4	6302	0	27	938
East Asian	1	0	1388	0	41
South Asian	5	0	0	6576	791
Mixed	463	28	7	43	2114
Don't know	1179	125	27	103	220
Other	1024	721	860	1483	2260
Total	401108	7179	2284	8240	7602
Grand total					426413

Table S12. SNPs, weights and allele frequencies (EAFs) by genetic ancestry included in the construction of the PGS model. EAFs were estimated in UK Biobank individuals without a diagnosis of cancer prior to the start of follow-up. SNPs log-OR were derived from OCAC published data. Genetic ancestry groupings are according to iadmix, derived as described in the Methods. [a]SNP variant name; [b]Position based on build 37

UKB rs number	SNP[a]	Chromosome	Position[b]	Reference Allele	Effect Allele	Effective allele frequency					SNP weight
						African	*East Asian*	*South Asian*	*European*	*Mixed*	
rs3820282	1_22468215_C_T	1	22468215	C	T	0.0129725	0.469395	0.218498	0.156408	0.166575	0.0808
rs12039431	1_38082122_G_A	1	38082122	G	A	0.0607647	0.285711	0.236216	0.261604	0.228389	0.0835
rs2165109	2_111818658_A_C	2	111818658	A	C	0.192274	0.439792	0.244416	0.262752	0.243606	0.0642
rs1470053	2_111915946_G_T	2	111915946	G	T	0.0535411	0.175901	0.124161	0.180358	0.130004	-0.0118
rs895412	2_113973964_T_C	2	113973964	T	C	0.220152	0.651806	0.515047	0.482094	0.480873	0.0518424
rs72831810	2_113979364_G_A	2	113979364	G	A	0.0138644	0.00326739	0.0318729	0.153851	0.0671076	0.0322626
rs1318778	2_177037831_C_G	2	177037831	C	G	0.921555	0.76886	0.854569	0.682241	0.800083	-0.1005
rs62276623	3_156402487_C_T	3	156402487	C	T	0.00618478	0.00213481	0.0104764	0.0512207	0.020229	0.3647
rs9869209	3_190531882_G_A	3	190531882	G	A	0.740272	0.540635	0.48322	0.287069	0.431405	-0.0668
rs34902361	4_70577859_G_A	4	70577859	G	A	0.0780033	0.25857	0.457768	0.359567	0.32834	-0.0574
rs10069690	5_1279790_C_T	5	1279790	C	T	0.65211	0.180385	0.254794	0.258192	0.31985	0.03511348
rs7705526	5_1285974_C_A	5	1285974	C	A	0.163759	0.388902	0.428613	0.326165	0.35161	0.05896018
rs2853677	5_1287194_G_A	5	1287194	G	A	0.740841	0.613398	0.424393	0.576878	0.542686	-0.0849278
rs2853669	5_1295349_A_G	5	1295349	A	G	0.0940012	0.348523	0.556485	0.314165	0.34821	-0.0797175
rs336126	5_54476556_G_A	5	54476556	G	A	0.564295	0.563656	0.689375	0.750894	0.698271	-0.0666
rs11782652	8_82653644_A_G	8	82653644	A	G	0.0751111	0.00201483	0.0752669	0.0701401	0.0730232	0.1294
rs9886651	8_128817883_A_G	8	128817883	A	G	0.117223	0.244756	0.373898	0.459623	0.372957	0.0759846
rs35916594	8_129069820_G_A	8	129069820	G	A	0.0907151	0.206246	0.262053	0.378383	0.289616	-0.0692237
rs6470611	8_129217984_G_C	8	129217984	G	C	0.720654	0.491205	0.518911	0.493026	0.568904	0.048478
rs10088755	8_129551633_G_A	8	129551633	G	A	0.201264	0.0368994	0.110264	0.130342	0.133607	-0.175939
rs62543619	9_16914716_G_A	9	16914716	G	A	0.0812363	0.00195378	0.0666319	0.197697	0.119724	-0.139195
rs10810671	9_16914835_A_C	9	16914835	A	C	0.598611	0.270901	0.41738	0.310832	0.423272	-0.101013
rs9406757	9_19044489_G_A	9	19044489	G	A	0.0886935	0.03476	0.136716	0.144173	0.122031	0.0788
rs10739885	9_106912892_G_A	9	106912892	G	A	0.784112	0.190829	0.433384	0.54919	0.549239	0.0581903
rs635634	9_136155000_C_T	9	136155000	C	T	0.091099	0.191112	0.142354	0.184616	0.158971	0.0931
rs7084454	10_21821274_G_A	10	21821274	G	A	0.385152	0.0197673	0.204676	0.322603	0.320934	0.0799
rs71479294	10_112011084_A_G	10	112011084	A	G	0.0184052	0.0853133	0.10912	0.156067	0.117628	0.0752
rs7139079	12_121415293_G_A	12	121415293	G	A	0.632772	0.423751	0.417992	0.592103	0.505022	-0.0593
rs76119208	15_91535329_G_T	15	91535329	G	T	0.149026	0.499368	0.203145	0.129374	0.176189	-0.080407
rs11657964	17_36100767_A_G	17	36100767	A	G	0.454542	0.750089	0.680961	0.60141	0.60422	-0.0582
rs169201	17_44790203_A_G	17	44790203	A	G	0.0147726	0.00306822	0.0644193	0.214643	0.115613	0.102556
rs12946636	17_46472432_C_G	17	46472432	C	G	0.526045	0.179061	0.190257	0.275425	0.294508	0.118166
rs10853591	18_21425852_T_C	18	21425852	T	C	0.653454	0.22575	0.545356	0.643269	0.600205	-0.0283
rs4808075	19_17390291_T_C	19	17390291	T	C	0.222741	0.00558947	0.15496	0.293903	0.206913	0.0796744
rs12982058	19_17409380_C_T	19	17409380	C	T	0.381481	0.322385	0.475558	0.518361	0.467587	-0.0612838
rs2070368	21_36080398_T_C	21	36080398	T	C	0.19369	0.602671	0.49375	0.399723	0.405025	-0.0599

Table S13. Mean, standard deviation (SD) and alpha parameters of the 36-SNP OC PGS. Mean and SD estimated from UK Biobank individuals without a diagnosis of cancer prior to the start of follow-up. α parameter estimated using SNPs frequencies from UK Biobank and SNPs log-OR from OCAC published data. Genetic ancestry groupings are according to iadmix, derived as described in the Methods.

	Women (N=226340)				
**Genetic ancestry**	*N*	*mean*	*L95%CI*	*U95%CI*	*SD*
European	212880	-0.249	-0.251	-0.248	0.322
African	4166	-0.407	-0.415	-0.399	0.255
East Asian	1524	-0.420	-0.432	-0.409	0.231
South Asian	3771	-0.431	-0.44	-0.422	0.278
Mixed	3999	-0.335	-0.344	-0.326	0.291

	Men (N=200073)				
**Genetic ancestry**	*N*	*mean*	*L95%CI*	*U95%CI*	*SD*
European	188228	-0.249	-0.251	-0.248	0.322
African	3013	-0.411	-0.42	-0.402	0.254
East Asian	760	-0.412	-0.43	-0.395	0.242
South Asian	4469	-0.442	-0.45	-0.434	0.278
Mixed	3603	-0.336	-0.346	-0.326	0.278

	Men and Women (N=426413)					
**Genetic ancestry**	*N*	*mean*	*L95%CI*	*U95%CI*	*SD*	*alpha*
European	401108	-0.250	-0.25	-0.248	0.322	0.223
African	7179	-0.409	-0.415	-0.403	0.254	0.178
East Asian	2284	-0.418	-0.427	-0.408	0.235	0.171
South Asian	8240	-0.437	-0.443	-0.431	0.278	0.194
Mixed	7602	-0.336	-0.342	-0.329	0.292	NA

Table S14. Mean, standard deviation (SD) and alpha parameters for BC PGS models. For East and South Asians, parameters estimated using data from the MyBrCa and SGBCC studies. For Europeans, parameters based on the prospective and validation sets from Mavaddat et al., 2019, and the published procedure from Mavaddat et al., 2023.

[a] Number of SNPs included in PGS models: all 313 SNPs from Mavaddat et al. (313-SNP PGS), UKB SNPs (309-SNP PGS), UKB SNPs excluding two CHEK2 SNPs (307-SNP PGS), no CHEK2 variants and imputation accuracy r2>0.5 (303-SNP PGS).

[b] αGLM is based on logistic regression adjusted for country in which studies were conducted and principal components.

[c] αRL is based on retrospective likelihood method as outlined in the Methods.

[d] Results based on 6394 controls and 6150 cases (MyBrCa and SGBCC studies) among Chinese and Malay women classified as 'East Asian' according to genetic data, with known age and age less than 80.

[e] Results based on 1000 controls and 568 cases (MyBrCa and SGBCC studies) among Indian women classified as 'South Asian' according to genetic data, with known age and age less than 80.

[f] Results in Europeans based on 22767 controls and 16151 cases. European women with known age and age less than 80.

PGS model[a]	Mean controls	Mean cases	SD controls	SD cases	Log OR per 1 SD	95%CI	α_{GLM}[b]	95%CI	α_{RL}[c]
**Chinese/Malay (East Asian)[d]**									
SNP313	0.137	0.358	0.550	0.553	0.398	0.362 - 0.435	0.294	0.267 - 0.322	0.327
SNP309	0.239	0.458	0.548	0.550	0.398	0.361 - 0.434	0.294	0.266 - 0.321	0.326
SNP307	0.582	0.801	0.548	0.549	0.398	0.361 - 0.434	0.294	0.266 - 0.322	0.326
SNP303	0.582	0.801	0.548	0.550	0.398	0.361 - 0.434	0.294	0.266 - 0.321	0.326
**Indian (South Asian)[e]**									
SNP313	-0.261	-0.017	0.620	0.589	0.418	0.307 - 0.528	0.329	0.243 - 0.416	0.327
SNP309	-0.168	0.079	0.616	0.589	0.422	0.311 - 0.532	0.331	0.245 - 0.417	0.331
SNP307	0.173	0.418	0.616	0.589	0.419	0.309 - 0.529	0.329	0.243 - 0.415	0.329
SNP303	0.175	0.421	0.615	0.587	0.421	0.311 - 0.532	0.330	0.244 - 0.416	0.330
**European[f]**									
SNP313	-0.424	-0.114	0.611	0.619	0.497	0.476 - 0.519	0.397	0.378 - 0.415	0.501
SNP309	-0.339	-0.032	0.608	0.617	0.495	0.474 -0.517	0.395	0.377 - 0.414	0.499
SNP307	-0.007	0.298	0.606	0.615	0.494	0.472 - 0.515	0.393	0.375 - 0.412	0.497

Table S15. Statistics on the MyBrCa and SGBCC studies; for cases and controls, number of individuals and mean/standard deviation (SD) age at diagnosis/interview (years).

Country	Study	Ethnicity	controls			cases		
			N	*mean age*	*SD*	*N*	*mean*	*SD*
**East Asian**								
Malaysia	MyBrCa	Chinese	1867	54.2	8.53	2294	51.7	10.8
Malaysia	MyBrCa	Malay	722	52.7	7.91	522	47.9	10.4
Singapore	SGBCC	Chinese	3252	50.3	10	2841	53.6	10.3
Singapore	SGBCC	Malay	553	48.7	8.98	493	51.3	9.41
**South Asian**								
Malaysia	MyBrCa	Indian	793	54.9	8.28	404	52.7	10.5
Singapore	SGBCC	Indian	207	51	9.84	164	54	9.92
